# Transduction of Systemically Administered Adeno-Associated Virus in the Colonic Enteric Nervous System and c-Kit Cells of Adult Mice

**DOI:** 10.3389/fnana.2022.884280

**Published:** 2022-06-06

**Authors:** Lixin Wang, Pu-Qing Yuan, Collin Challis, Sripriya Ravindra Kumar, Yvette Taché

**Affiliations:** ^1^Vatche and Tamar Manoukian Division of Digestive Diseases, Department of Medicine, CURE/Digestive Diseases Research Center, David Geffen School of Medicine, University of California, Los Angeles, Los Angeles, CA, United States; ^2^Veterans Affairs Greater Los Angeles Healthcare System, Los Angeles, CA, United States; ^3^Division of Biology and Biological Engineering, California Institute of Technology, Pasadena, CA, United States

**Keywords:** adeno-associated virus, colon segments, enteric nervous system, interstitial cells of Cajal (ICCs), immunohistochemistry, mouse, neuronal tracing

## Abstract

Systemic delivery of adeno-associated virus (AAV) vectors transduces the enteric nervous system. However, less is known on the mapping and morphological and neurochemical characterization in the adult mouse colon. We used AAV9-CAG-GFP (AAV9) and AAV-PHP.S-hSyn1-tdTomato farnesylated (PHP.S-tdTf) to investigate the segmental distribution, morphologies and neurochemical coding of the transduction. The vectors were retro-orbitally injected in male and female adult mice, and 3 weeks later, the colon was prepared for microcopy with or without immunohistochemistry for neuronal and non-neuronal markers. In contrast to the distributions in neonatal and juvenile rodents, the AAV transduction in neurons and/or nerve fibers was the highest in the proximal colon, decreased gradually in the transverse, and was sparse in the distal colon without difference between sexes. In the proximal colon, the AAV9-transduced myenteric neurons were unevenly distributed. The majority of enteric neurons did not have AAV9 expression in their processes, except those with big soma with or without variously shaped dendrites, and a long axon. Immunolabeling demonstrated that about 31% neurons were transduced by AAV9, and the transduction was in 50, 28, and 31% of cholinergic, nitrergic, and calbindin-positive myenteric neurons, respectively. The nerve fiber markers, calcitonin gene-related peptide alpha, tyrosine hydroxylase or vasoactive intestinal polypeptide co-localized with AAV9 or PHP.S-tdTf in the mucosa, and rarely in the myenteric plexus. Unexpectedly, AAV9 expression appeared also in a few c-Kit immunoreactive cells among the heavily populated interstitial cells of Cajal (ICC). In the distal colon, the AAV transduction appeared in a few nerve fibers mostly the interganglionic strands. Other types of AAV9 and AAV-PHP vectors induced a similar colonic segmental difference which is not colon specific since neurons were transduced in the small intestine and gastric antrum, while little in the gastric corpus and none in the lower esophagus.

**Conclusion:**

These findings demonstrate that in adult mice colon that there is a rostro-caudal decrease in the transduction of systemic delivery of AAV9 and its variants independent of sex. The characterization of AAV transduction in the proximal colon in cholinergic and nitrergic myenteric neurons along with a few ICC suggests implications in circuitries regulating motility.

## Introduction

Adeno-associated virus (AAV) vectors are commonly used for gene delivery in human gene therapy and functional studies in experimental research (Wang et al., 2019; Haggerty et al., 2020; Abulimiti et al., 2021). Additionally, AAV gained attention as a tool for neuronal tracing (Betley and Sternson, 2011; Lerner et al., 2016; Luchicchi et al., 2021). Systemic delivery of AAV provides a non-invasive approach for gene delivery to the peripheral nervous system (Foust et al., 2009; Chan et al., 2017). In particular, AAV8 and/or AAV9 injected intravenously was reported to transduce the enteric nervous system in the gastrointestinal (GI) tract of neonatal and juvenile mice and guinea pigs (Gombash et al., 2014, 2017; Schuster et al., 2014; Buckinx et al., 2016). AAV8 and 9 showed higher transduction efficiency than AAV1, 5, and 6 in the mouse enteric nervous system (Gombash et al., 2014). In adult mice, we performed iv injection of a multicolor AAV vector system for sparse neural tracing, and found that the transduction was prominent in enteric neurons of the proximal colon, while few in the distal colon (Wang et al., 2020). Another study using tail vein injection of AAV2/9 at low doses to label sparsely the colonic enteric neurons in adult mice addressed the projection orientation and length of individual neurons and did not present evidence of different segmental distributions of AAV2/9-transduced neurons (Li et al., 2019). However, so far, the capability of AAV to transduce enteric neurons along the colonic segments of the adult mouse colon and the characterization of transduced cells have not been thoroughly investigated.

The novel serotype AAV-PHP.S was derived from AAV9 and has been reported to be more effective to transduce neurons in the periphery than in the brain (Chan et al., 2017). In addition, farnesylation is a posttranslational modification of proteins (Maltese, 1990) that enhances membrane binding signal of fluorescent protein in tracers and viral vectors, and can be leveraged to promote labeling of nerve fibers (Fagoe et al., 2014; Yip et al., 2015; Kadala et al., 2018). A previous study demonstrated the utility of AAVs to deliver farnesylated fluorophores to label neuronal processes in the brain (Challis et al., 2019), and it is yet to be established in nerve fibers of the enteric nervous system.

The objectives of the present study were to map and characterize the morphology and neurochemical identity of transduced cells in the colon of adult male and female mice following systemic administration of AAV9 and AAV-PHP.S-hSyn1-tdTomato farnesylated (PHP.S-tdTf). AAV9 was used to assess the transduction in the adult mice complementary to previous studies performed in neonatal and juvenile rodents (Gombash et al., 2014, 2017; Buckinx et al., 2016). We also used PHP.S-tdTf, an engineered AAV9 variant vector with farnesylated fluorophore as a new tool to profile the distribution of transduced nerve fibers. The focus on the colon was to gain insights into the profile of neurons responsive to systemic AAV vectors for potential development of neuromodulation and drug therapies to treat colonic diseases, such as inflammatory bowel and colon cancer (Zygulska et al., 2018; Mogilevski et al., 2019; Schiller et al., 2021). Based on the segmental difference in the transduction of the colon, we assessed whether such a feature was specific to the colon or also found in other parts of the GI tract from the esophagus to ileum. Then, we focused on the proximal colon which displayed the densest transduction to characterize further the AAV-transduced neurons and nerves by immunofluorescent staining of neuronal and non-neuronal markers. We examined the relationship of neural structures, and between neuronal and non-neuronal cells, using the pan-neuronal markers: HuC/D, protein gene product (PGP) 9.5, and NeuN; the markers of the most representative excitatory and inhibitory motor and sensory neurotransmitters: choline acetyltransferase (ChAT), neuronal nitric oxide synthase (nNOS) and calbindin; nerve fibers: calcitonin gene related peptide alpha (αCGRP), tyrosine hydroxylase (TH), and vasoactive intestinal peptide (VIP); glial cells: glial fibrillary acidic protein (GFAP); macrophage: ionized calcium binding adaptor molecule 1 (Iba1) and the interstitial cells of Cajal (ICC): c-Kit (a proto-oncogene encoding the receptor tyrosine kinase protein, a.k.a. CD117). Lastly, we determined whether the selectivity of AAV transduction in the proximal colon was serotype specific using different AAV9 vector variants and mice strain including the common mouse strain, C57BL/6J as well as in ChAT-Cre and nNOS-Cre mice which are mouse lines selective for the two important neurotransmitters involved in the colonic motility under physiological and pathological conditions (Broad et al., 2019; Gould et al., 2019).

## Materials and Methods

### Animals

The following 8–12 weeks old male and female mice from Jackson Laboratories (Sacramento, CA) were used: C57BL/6J (000664), ChAT-IRES-Cre mice (028861), and Nos1-Cre (017526). They were maintained 2 per cage under standard conditions. Animal care and experimental procedures followed institutional ethic guidelines and conformed to the requirements of federal regulations for animal research conduct. All procedures were approved by the Animal Research Committee at Veterans Affairs Greater Los Angeles Healthcare System (animal protocol #07013-17).

### Retro-Orbital Injection of AAV

The AAV types, suppliers, and doses used are detailed in Table 1. Mice were anesthetized with 2.5% isoflurane in oxygen and injected unilaterally into the retro-orbital venous sinus with the vectors diluted in phosphate-buffer saline (PBS) (60 μl/mouse) as in our previous study (Wang et al., 2020). The injection was performed using a BD Veo Insulin Syringe with BD Ultra-Fine 6 mm × 31 G needle (324911, Becton, Dickinson and Company, Franklin Lakes, NJ). The highest dose was 1 × 10^12^ Genome Copy (GC)/mouse to avoid toxicity (Chan et al., 2017; Challis et al., 2019).

**Table 1:** AAV types, doses and suppliers.

**AAV type**	**Supplier**	**Doses**	**Reference**
		**(GC/mouse)**	
AAV9-CAG-EGFP	Addgene	3.3 ×10^11^, 1 ×10^12^	37825-AAV9, addgene.com
AAV-PHP.S-hSyn-tdTomato-farnesylate	Dr. V. Gradinaru Lab, Caltech	1 ×10^12^	https://www.addgene.org/viral-service/aav-prep/gradinaru-php/ https://clover.caltech.edu/
AAV-PHP.S-CAG-NLS-eGFP		3.3 ×10^11^, 1 ×10^12^	
AAV-PHP.S-CAG-DIO-EYFP		3.3 ×10^11^	
AAV9-icap-CAG-NLS-GFP		3.3 ×10^11^	

*All vectors were single-stranded. For the designs and constructions of the AAV vectors please refer to the website of Addgene and Caltech Clover Center and publications (Chan et al., 2017; Deverman et al., 2018; Challis et al., 2019). CAG: a promoter constructed from gene sequences of cytomegalovirus, chicken beta-actin and rabbit beta-globin (Niwa et al., 1991); EGFP, enhanced green fluorescent protein; hSyn, human synapsin 1 gene promoter*.

### Tissue Processing

Tissues were harvested from mice euthanized by an overdose of isoflurane. The whole colon was removed from ileocecal junction to the end of distal colon at the level of pelvic brim where runs the iliac artery. The colon was flat-pinned on a Sylgard™ 184 silicone elastomer (Electron Microscopy Science, Hatfield, PA), and fixed in 4% paraformaldehyde in 0.1M phosphate buffer. To compare the labeling of the proximal colon with other GI segments, we also collected the esophagus, gastric corpus, gastric antrum, duodenum, jejunum, and ileum; as well as the nodose ganglia of the vagus, dorsal root ganglia at L1 and L6, celiac-superior mesenteric ganglion, and pelvic ganglia from some mice as previously (Wang et al., 2020). For 3D imaging, the colon with the whole thickness was cleared using passive CLARITY with the hydrogel containing 4% acrylamide without paraformaldehyde (A4F0) (Yang et al., 2014), or pretreated with 2% Triton X 100–0.01M phosphate buffered saline (TPBS) to enhance penetration of the primary antibodies. Because the regions of the proximal colon with mucosal folds were poorly immunolabeled, three other methods were used: (1) flat colonic wall with removal of the mucosa; (2) whole mount preparations of the submucosal plexus (SMP) and myenteric plexus (MP); and (3) transverse frozen sections at 14 or 200 μm thickness.

### Immunohistochemistry

The colonic samples were incubated according to the following steps: (1) 10% normal donkey serum in 0.3% Triton-X 100-PBS for 3 h at room temperature (RT); (2) primary antibodies detailed in Table 2 in 0.3% Triton-X 100-PBS 2 h at RT, then 4°C 2–5 days; (3) secondary antibody for 3 h at RT for whole mount and sections, or overnight at 4°C for thick tissues; and (4) mount and coverslip in Vectashield (Vector Laboratories, Burlingame CA) for whole mount and thin sections, or RIMS (refractive index matching solution) (Yang et al., 2014) for thick colon walls and sections at 200 μm which were sealed by glass coverslip on a frame (iSpacer, Sunjin Lab, Taiwan, R.O.C.) (Yuan et al., 2021).

**Table 2:** Primary antibodies.

**Antibody**	**Species**	RRID^*^	**Source**	**Catalog number**	**Dilution**
ChAT	Goat	AB_2079751	Millipore	144p	1:200
nNOS	Rabbit	AB_2152469	Abcam	ab76067	1:1,000–1:2,000
Calbindin	Rabbit	AB_10000340	Swart	CB38	1:2,000
PGP9.5	Rabbit	AB_10891773	Abcam	ab108986	1:1,000
HuC/D	Mouse	AB_221448	Life Technologies	A-21271	1:200
NeuN	Rabbit	AB_2532109	Abcam	ab177487	1:1,000
αCGRP	Rabbit	AB_518147	Peninsula	T-4032	1:2,000
VIP	Rabbit	AB_2890602	CURE/UCLA	ab7913	1:1,000
TH	Rabbit	AB_390204	Millipore	AB152	1:1,000–1:2,000
GFAP	Rabbit	AB_305808	Abcam	ab7260	1:2,000–1:4,000
c-Kit	Goat	AB_354750	R&D System	AF1356	1:200
Iba1	Rabbit	AB_839504	WAKO	019-19741	1:1,000

* *: RRID, Research Resource Identifiers*.

Microscopic images were acquired in Zeiss confocal microscopes (LSM 710 and 880), and low magnification images were stitched by Keyence microscope (BZ-X710). The image segmentation, quantitation and visualization were performed using Imaris 9.6 and 9.7 for neuroscientists (Bitplane Inc., Concord, MA). AAV-transduced and immunolabeled neurons in the myenteric plexus were counted in 5–7 images/mouse using “Spots” module of Imaris, and calculated in a standardized ganglionic area (0.25 mm^2^) defined by regions of interest. Due to the irregular shapes, and many discontinued cellular parts or connected cells, the volume of Iba1-ir cells was measured using the “Surface” module. The density was calculated and expressed as percentage in the volume of image region. AAV9/c-Kit cells were manually counted in a fluorescent microscope because the small numbers of double-labeled cells were embedded the dense networks c-Kit positive cells. The cells were counted in each MP whole mounts (~6–9 mm^2^) with a 20 × objective.

### Statistical Analysis

Statistical analysis was performed using SigmaPlot 14 (Systat Software, Inc., San Jose, CA, USA). Data are presented as mean ± SEM. Comparisons between male and female mice and between naïve and AAV9 injected mice were performed using Student's *t*-tests. *P* < 0.05 were considered significant. No outliers were excluded based on Grubbs' test (https://www.graphpad.com/quickcalcs/Grubbs1.cfm, Graph Pad).

## Results

### Mouse Proximal Colon Divisions

**Figure 1:**
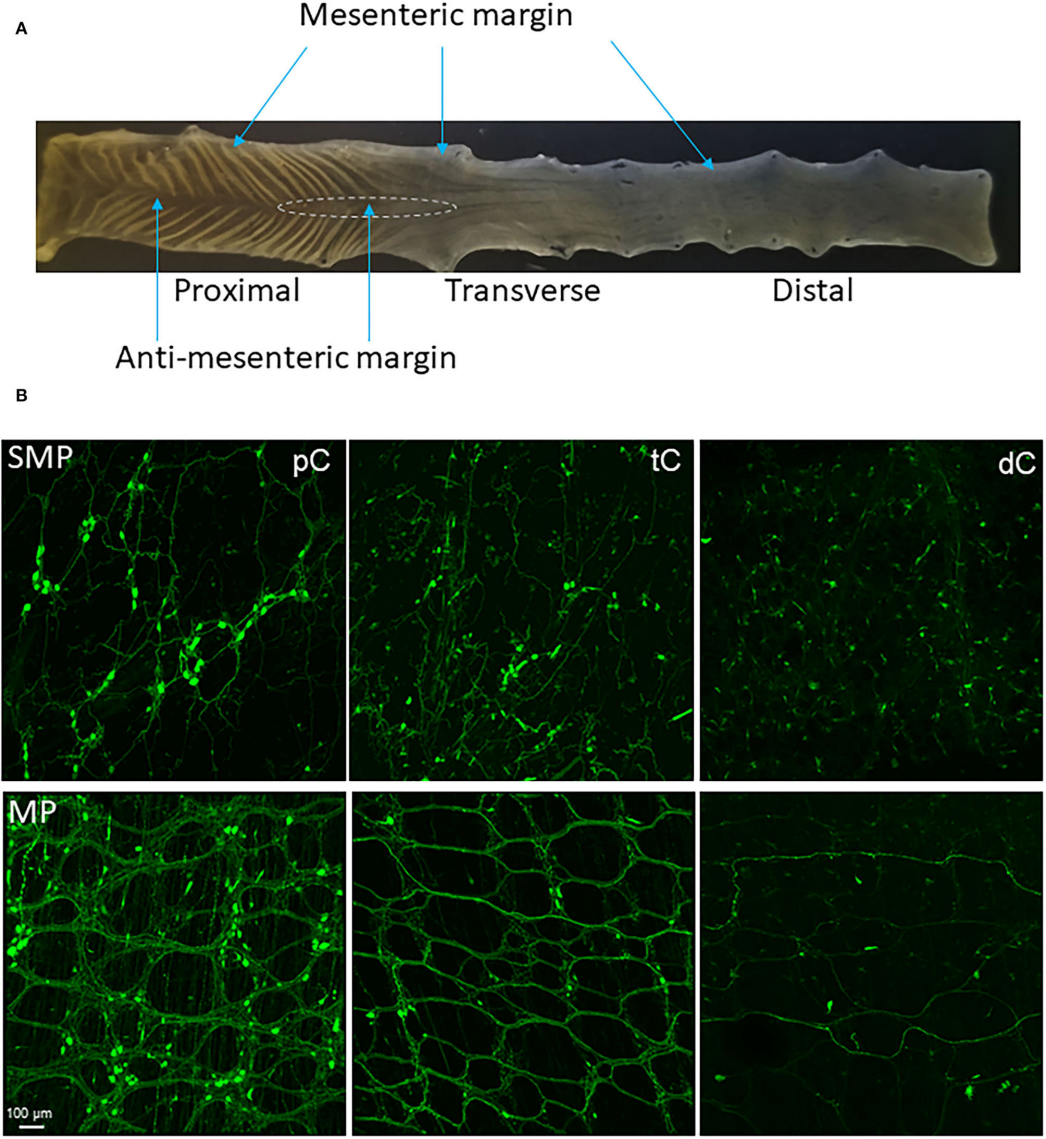
**(A)** Segments and regions of an adult mouse colon. The specimen was opened along mesenteric margin with mucosa on the top. The proximal colon had mucosa folds aligned diagonally on each side and each fold ended at the antimesenteric margin. The proximal and transverse colon could be divided by the disappearance of the mucosa folds. There is no clear landmark between the transverse and distal colon. About 1.5 cm caudal the proximal colon could be the transverse colon. The area marked by a dashed line is a hypoganglionic region in the antimesenteric margin located at the aboral end of the proximal colon and oral segment of the transverse colon. **(B)** Segmental difference: representative photomicrographs of AAV9 transduction in the mouse colon. AAV9-CAG-GFP was retro-orbitally injected at 3.3 x 10^11^ GC/mouse 3 weeks before. AAV9-labeled the enteric neurons and nerve fibers were abundant in the proximal colon (pC), reduced in the transverse colon (tC) and only a few nerve fibers in the distal colon (dC). Upper row: submucosal plexus (SMP); lower row: myenteric plexus (MP). Scale: 100 μm, same for all panels.

**Figure 2:**
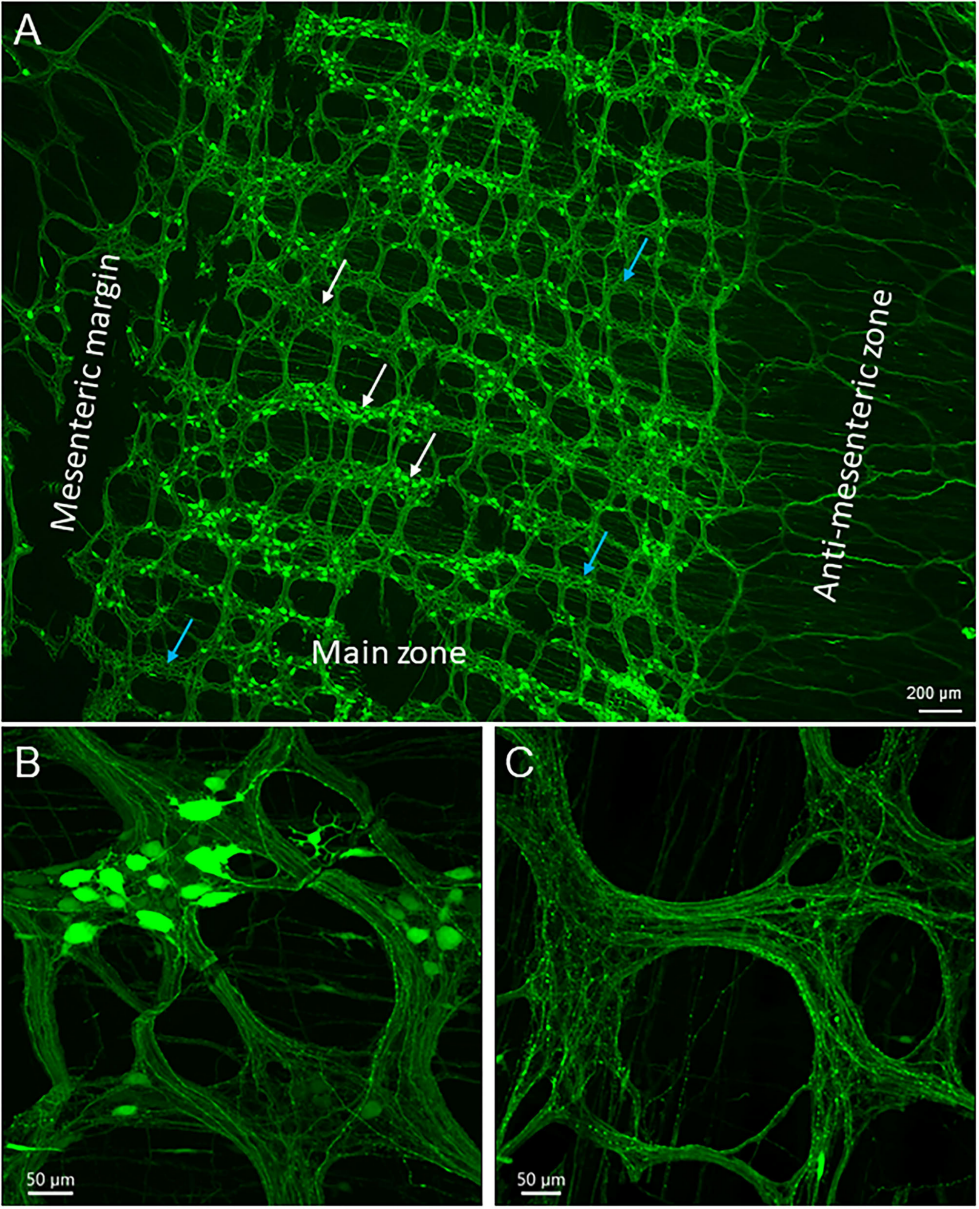
Uneven distribution of AAV9 transduced neurons in the myenteric plexus in the mouse proximal colon by retro-orbital injection of the vector. The two doses of 3.3 x 10^11^ and 1 x 10^12^ GC/mouse result in the same pattern. Numbers of AAV9-labeled neurons vary largely from area to area among the ganglia: numerous to none. Labeled nerve fibers are shown in all areas. **(A)** Low magnification stitch image of one side of the colonic wall in the segment about 2–3 cm from the ileocolonic junction. Blue arrows in A indicate areas with ganglia lacking labeled neurons. In many rows, the myenteric ganglia fused together circumferentially (white arrows, with or without AAV9 transduced neurons). The circumferentially projecting nerves are parts of the intramuscular arrays in the circular muscle layer. **(B,C)** images demonstrate myenteric ganglia with many, a few, and none AAV9-transduced neurons. Scales as indicated in each image.

Based on the structures and morphology of the proximal colon and observations of the myenteric plexus with AAV transduction and immunostaining, we divided the mouse proximal colon into 4 longitudinal zones (Figures 1, 2A): two main zones that have diagonal mucosal folds on each site, and underneath a dense network of nerve fibers and large ganglia in the myenteric plexus; one mesenteric margin attached by the mesentery, and one anti-mesenteric margin that was obvious in the proximal colon with the formation of mucosal folds on each side. The mesenteric margin contained the myenteric plexus that was sparse with small ganglia whereas there are dense circumferential nerve fibers including the extrinsic fibers entering or exiting the colon and those in the circular muscular layer. In a caudal segment of the proximal colon and the adjacent transverse colon (Figure 1), the anti-mesenteric margin mainly contained the circular muscles, circumferential nerve fibers and scattered neurons.

### Distributions of Neurons and Nerve Fibers Transduced by AAV9-CAG-GFP (Addgene)

AAV9-CAG-GFP was tested first at 3.3 × 10^11^ (male 5 and female 4) and 1 × 10^12^ (male 5 and female 1) GC/mouse *via* retro-orbital injection. The transduction in myenteric ganglia of the proximal colon was similar in male vs. female mice (49.2 ± 10.2 vs. 45.5 ± 4.3 cells/0.25 mm^2^; *p* > 0.05) at 3.3 × 10^11^ GC/mouse. AAV9 at 1 x 10^12^ GC/mouse transduced higher numbers of neurons than those observed at 3.3 × 10^11^ GC/mouse (62.3 ± 4.1 vs. 48.1 ± 6.4 cells/0.25 mm^2^; *p* < 0.05). Studies combined with immunohistochemistry were performed in samples from the male and female mice injected with 3.3 × 10^11^ GC/mouse, since there was no sex difference.

The AAV9-transduced neurons and nerve fibers were abundant in both submucosal and myenteric plexuses of the proximal colon and reduced in the transverse colon while only sparse nerve fibers were observed in the distal colon (Figure 1B). The AAV9 transduced neurons were not evenly distributed in the myenteric ganglia of the proximal colon (Figure 2A), as shown by some ganglia containing numerous neurons (Figure 2B) and others with only a few or none (Figure 2C). In the main zones, some myenteric ganglia were fused together in the circular rows as shown by neurons labeled by AAV9 or HuC/D (Figure 2A and Supplementary Figure 1). In the submucosal plexus of the proximal colon, AAV9-transduced neurons and nerve fibers were distributed mostly along the mucosa folds (Supplementary Figure 2A).

**Figure 3:**
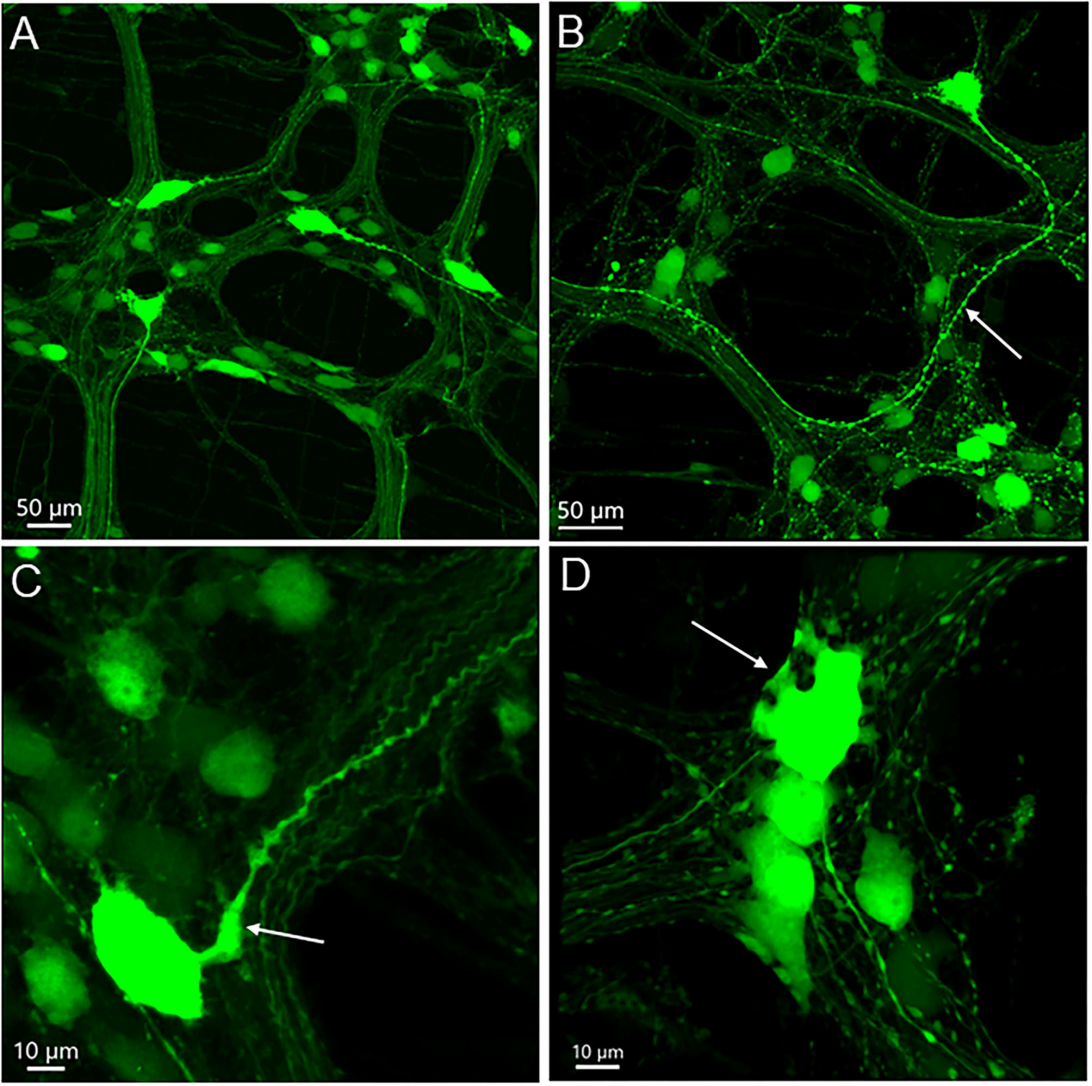
AAV9-transduced neurons in the myenteric plexus in whole mount preparations of the mouse proximal colon. AAV9-CAG-GFP was retro-orbitally injected at 3.3 x 10^11^ GC/mouse 3 weeks before. **(A)** Axons of a few large neurons project out of the ganglia in different directions, and many neurons are small and medium in size without labeled processes. **(B)** One neuron has a large soma and a thick varicose axon (arrow). **(C)** The axon of a big neuron has enlarged initial part (arrow) and varicosities *en route*. **(D)** The arrow points to some short thick dendrites of one neuron at the edge of one ganglion. Scales as indicated in each image.

The majority of AAV9-labeled neurons in the myenteric ganglia had round and oval shaped somas and were of small to medium sizes, and many did not have labeled processes (Figures 2B, 3). The fluorescence was localized in the neuronal cytoplasm and nuclei, with a wide range of brightness. Neurons bearing oversaturated fluorescence were often observed, and they were in various shapes and sizes (Figure 2B and Supplementary Figure 3). Some neurons displayed distinct morphology: a large soma and a long varicose axon. The somas were either smooth or had short chubby dendrites. Some of their axons had enlarged nodes in the initial segment and some neurons had multiple irregular dendrites. Often, neurons of that type were located in the outer zone of the myenteric ganglia, and the dendritic “feet” aligned along the edge (Figure 3D and Supplementary Figure 3). Multipolar neurons were rarely found.

**Figure 4:**
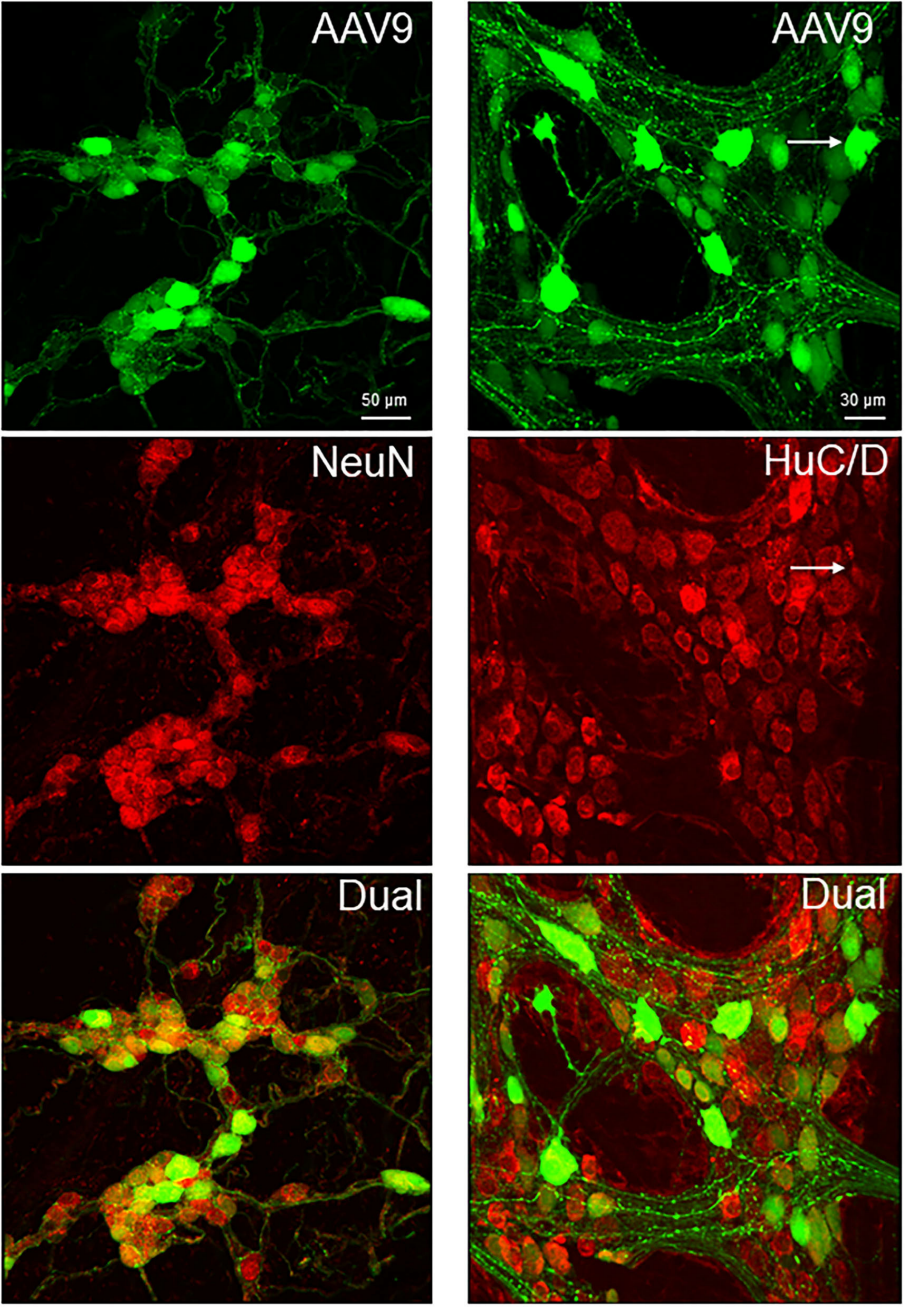
Dual labeling of AAV9 transduction and immunofluorescence of pan-neuronal markers, NeuN in the submucosal plexus (left panels) and HuC/D myenteric plexus (right panels) in whole mount preparations of the mouse proximal colon. AAV9-CAG-GFP was retro-orbitally injected at 3.3 x 10^11^ GC/mouse 3 weeks before. The submucosal neurons were labeled by NeuN, due to a lot of non-specific labeling by the HuC/D antibody.

**Figure 5:**
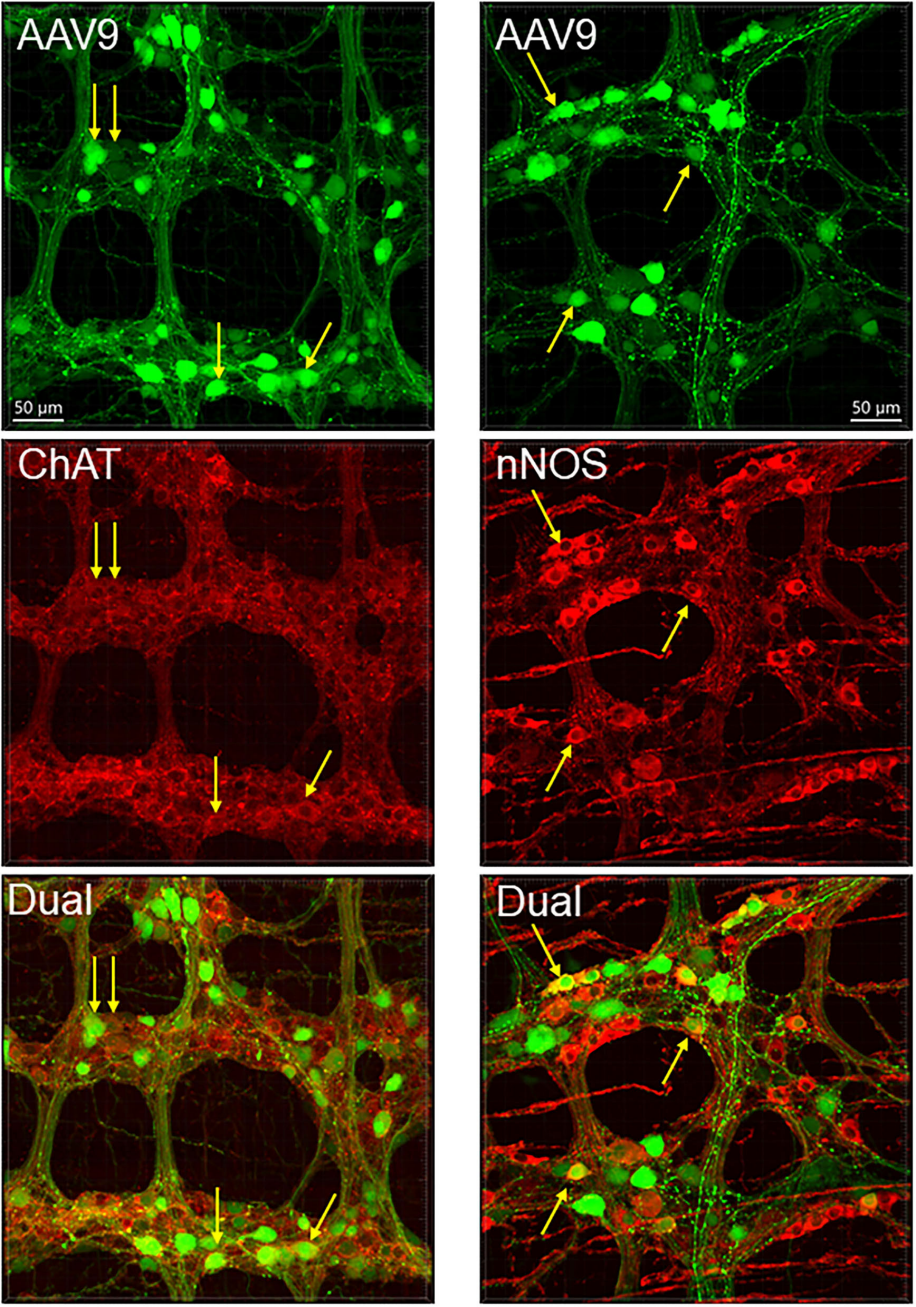
AAV9 transduction in the myenteric plexus whole mount preparations of the mouse proximal colon combined with immunofluorescence of choline acetyl transferase (ChAT) and neuronal nitric oxide synthetase (nNOS). AAV9-CAG-GFP was retro-orbitally injected at 3.3 x 10^11^ GC/mouse 3 weeks before. Arrows indicate some of the dual labeled neurons.

**Figure 6:**
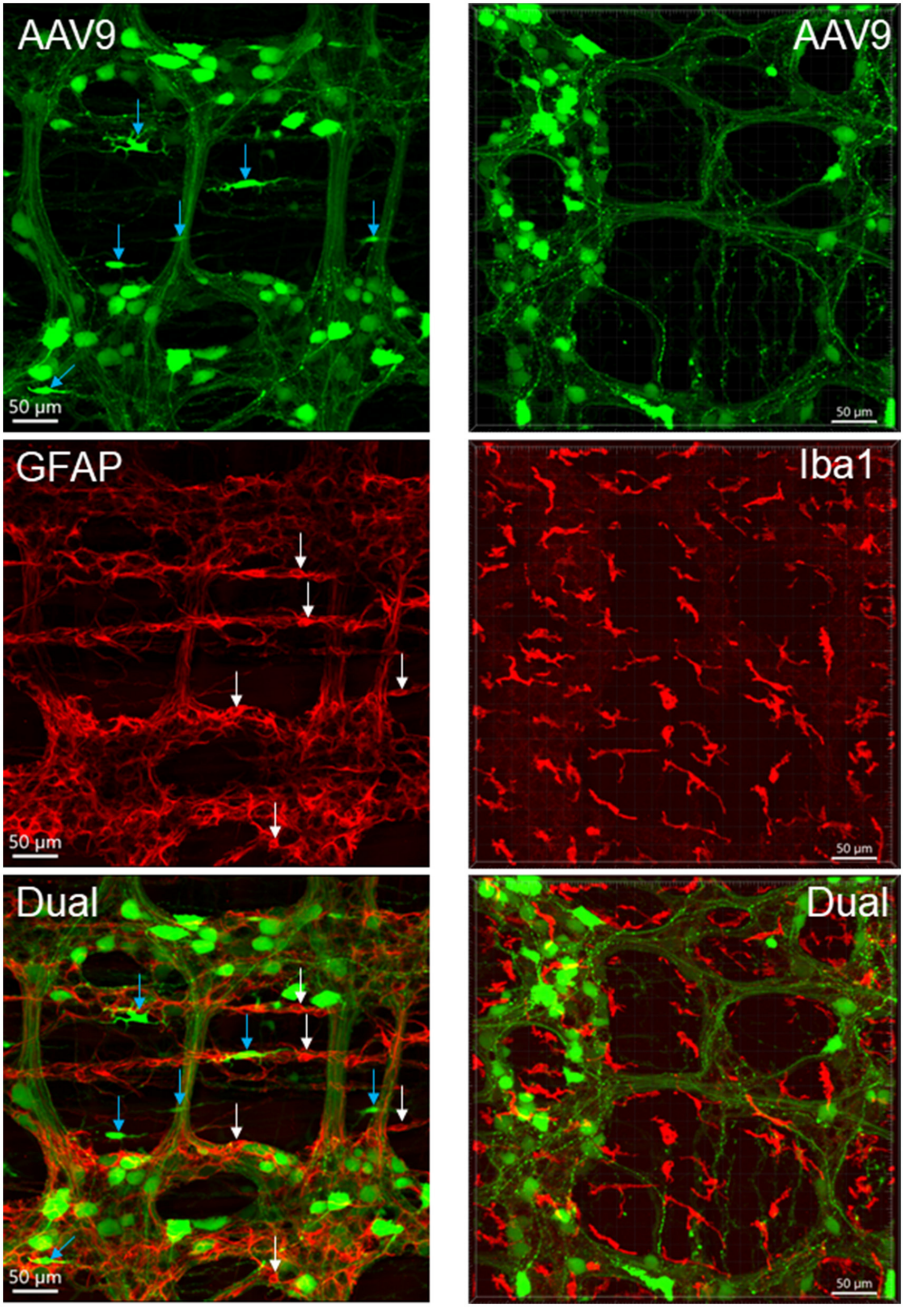
AAV9 transduction in the myenteric plexus whole mount preparations of the mouse proximal colon combined with immunofluorescence of glial fibric acid protein (GFAP) for glial cells (left panels) and ionized calcium binding adaptor molecule 1 (Iba1) for macrophages (right panels). AAV9-CAG-GFP was retro-orbitally injected at 3.3 x 10^11^ GC/mouse 3 weeks before. No AAV9-transduced cells were GFAP-ir or Iba1-ir. White arrow indicate GFAP-ir glial cell bodies, and blue ones indicate AAV9 transduced non-neuronal cells which were not GFAP-ir. Many Iba1-ir cells were located near or inside the ganglia and interganglionic strands (bottom-right panel).

The AAV9-labeled cells in the enteric ganglia were neurons as identified by the colocalization with the immunofluorescence of HuC/D, NeuN, ChAT, nNOS, calbindin, and VIP in the myenteric and/or submucosal ganglia (Figures 4, 5, Supplementary Figures 4–7), but not with that of GFAP (Figure 6, Supplementary Figure 8). Quantitative analyses using Imaris showed that AAV9 transduced about 30.5% of myenteric neurons in the proximal colon, of which 36, 27, and 6% were ChAT-, nNOS-, and calbindin-immunoreactive (ir), respectively (Table 3). In the differential neuronal populations, 50, 28, and 31% of cholinergic, nitrergic, and calbindin neurons were transduced by AAV9 (Table 3). In the submucosal plexus, the majority of AAV9-tranduced neurons were calbindin-ir, to less extent ChAT-ir and VIP-ir, and with few nNOS-ir (Supplementary Figures 4–7).

**Table 3:** Percentage of neurons transduced by iv AAV9 (of HuC/D immunolabeled neurons), and percentage of AAV9-transduced neurons immunolabeled by selective neurotransmitter markers.

	**Immunolabeling of**	**AAV9 transduction in**
	**AAV9 neurons (%)**	**immunolabeled neurons (%)**
HuC/D	/	30.5 ± 2.7
ChAT	35.9 ± 1.9	50.1 ± 2.0
nNOS	26.8 ± 2.0	28.0 ± 1.7
Calbindin	5.7 ± 1.5	31.0 ± 3.8

*Data are mean ± SEM, n = 5–8 mice*.

**Figure 7:**
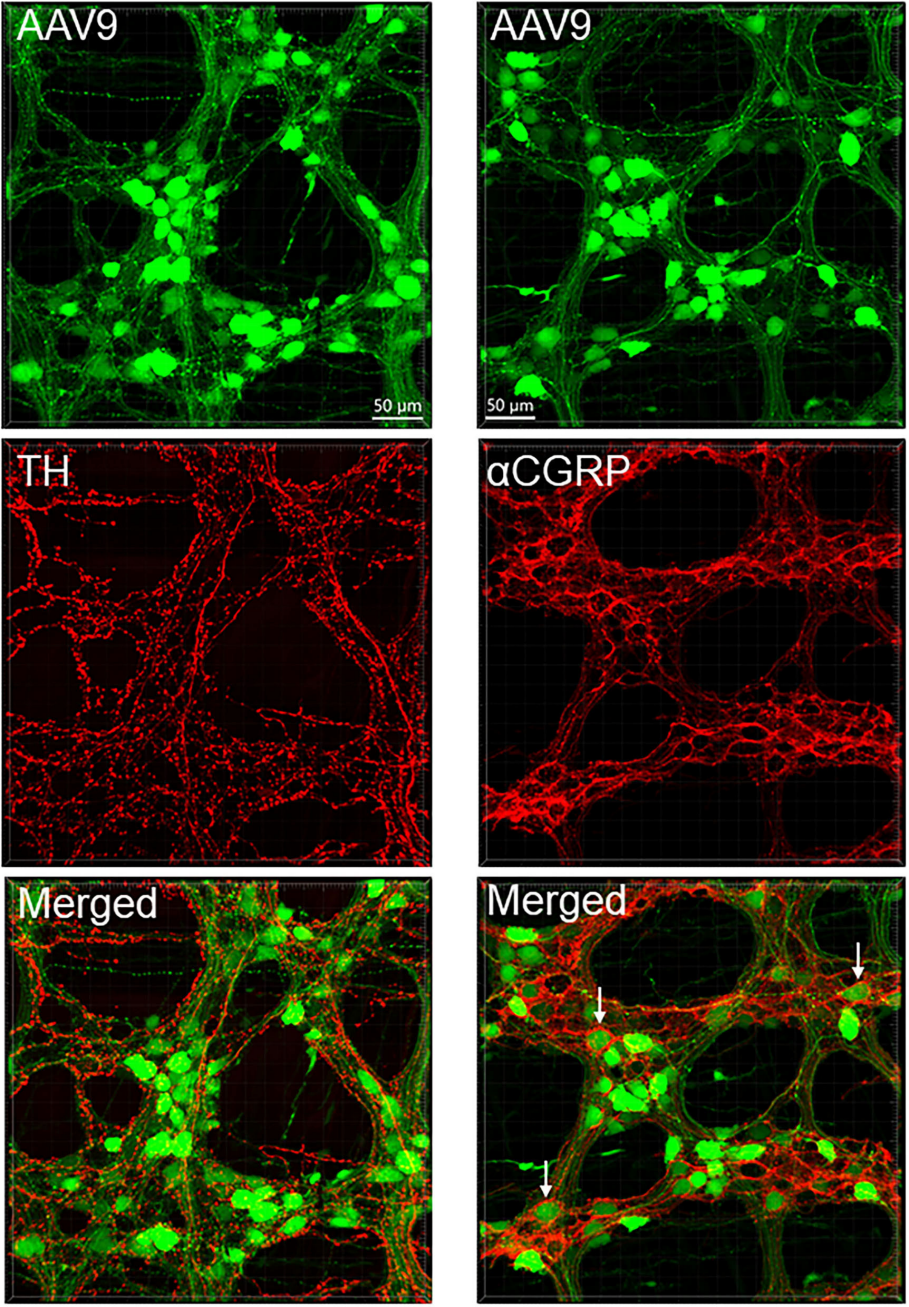
AAV9 transduction in the myenteric plexus whole mount preparations of the mouse proximal colon combined with immunofluorescence of tyrosine hydroxylase (TH, left panels) and calcitonin gene related peptide-alpha (αCGRP, right panels). AAV9-CAG-GFP was retro-orbitally injected at 3.3 x 10^11^ GC/mouse 3 weeks before. TH and αCGRP immunofluorescence (red) barely co-existed with AAV9 (green). Notably, αCGRP immunofluorescent nerve fibers surrounded neuronal somas more frequently (arrows) than TH.

The AAV9-tranduced nerve fibers were located in the myenteric ganglia, interganglionic strands, and intramuscular arrays in the circular muscle layer (Figure 2A and Supplementary Figure 9). Longitudinal interganglionic strands were prominent (Figure 2A and Supplementary Figures 9A,B), and some fibers could be traced aborally in a long distance from the caudal proximal colon to the distal colon. In contrast, the circumferential interganglionic strands were not well-formed, and instead, were scattered along the circular direction of the ganglia and small bundles crossing spaces among the ganglia (Figure 2A, Supplementary Figure 10). A few fibers with AAV9 transduction projected along longitudinal muscles (Supplementary Figure 9G). Occasionally, thick beaded intraganglionic nerve endings were observed in the submucosal and myenteric plexus (Supplementary Figures 9H,I). Notably, TH, CGRP, and VIP immunoreactivity rarely co-localized with AAV-9 in nerve fibers in the myenteric ganglia (Figure 7, Supplementary Figures 7, 11).

In the mesenteric margin, nerve fibers entered or existed the colon. The nearby myenteric plexus was less well-lined up with smaller ganglia compared with those in the main zone (Figure 2A and Supplementary Figures 9D,E). The myenteric plexus in the antimesenteric margin of the rostral half of the proximal colon was similar as in the main zones, while in the aboral segment and the beginning of the transverse colon, there were scattered neurons and circumferential fiber bundles connecting the ganglia in the two main zones (Figure 2A and Supplementary Figures 9E,F). As the AAV9-transduced neurons started to disappear from the mid portion of the transverse colon to the distal colon, AAV9-labeled nerve fibers remained in small longitudinal bundles with crossing to terminate in or passing the myenteric ganglia, and a few circular nerve fibers and terminals were also observed in the ganglia (Supplementary Figure 12).

### Macrophages and Interstitial Cells of Cajal in the Proximal Colon of Mice Injected With iv AAV9-CAG-GFP

The macrophages labeled by Iba1 were similar quantitatively in the proximal colon of AAV-9-injected mice compared with naïve mice (2.3 ± 0.2 vs. 2.4 ± 0.2% volume of the regions measured in the myenteric plexus preparations). The distribution and morphologies of Iba1-ir cells were similar in the vector-injected and naïve mice (Supplementary Figure 13). The Iba1-ir cells in the submucosal layer were more abundant than in the myenteric plexus and muscular layers, and the morphologies were also different as many cells had round bodies, while those close to the myenteric plexus had irregular shape with processes and were distributed near the myenteric network and inside the ganglia (Figure 6). Iba1-positive cells were found closely adjacent to AAV9-transduced neurons and nerve fibers (Supplementary Figures 13E,F).

**Figure 8:**
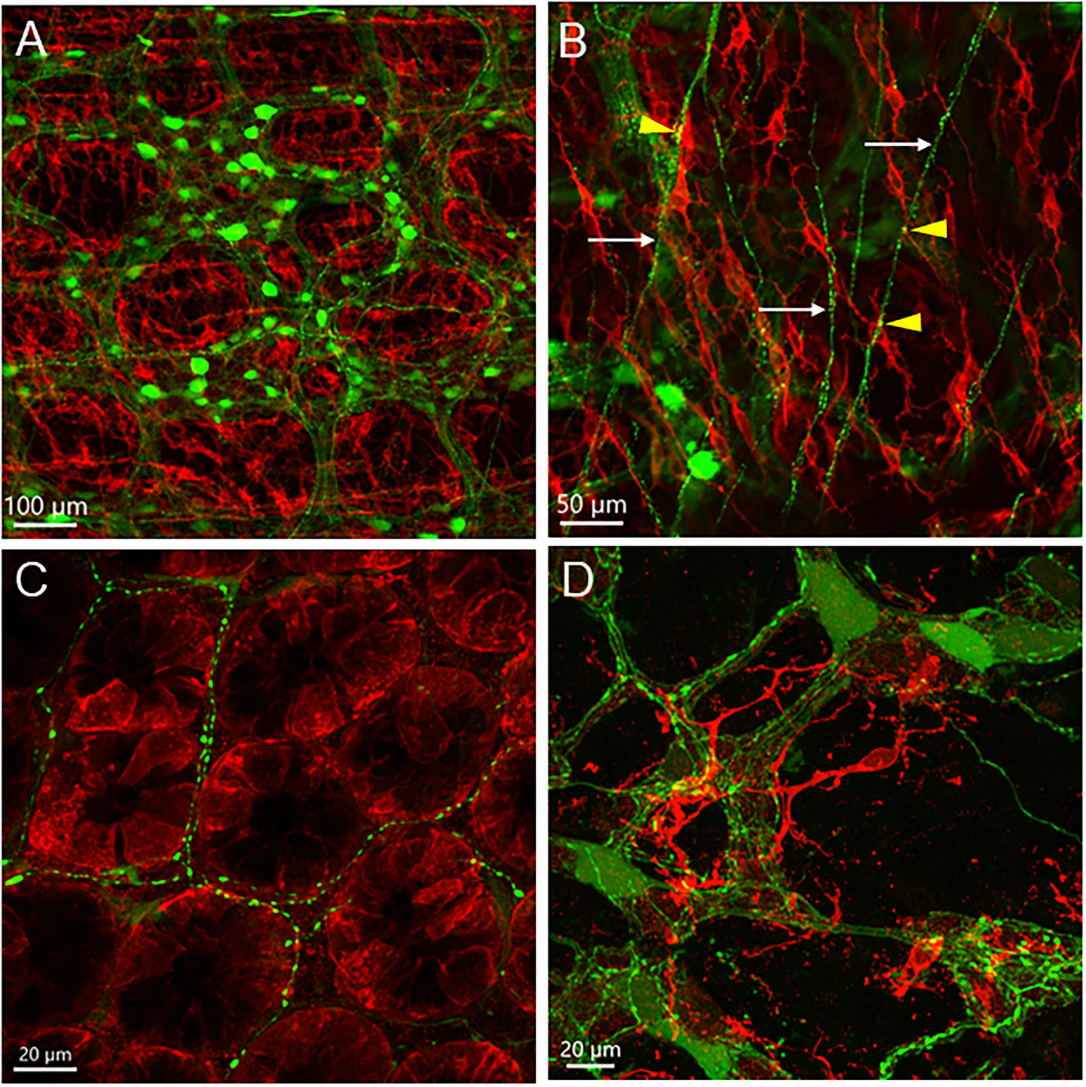
c-Kit-immunofluorescent cells (red) in the mouse proximal colon with AAV9 transduction (green). AAV9-CAG-GFP was retro-orbitally injected at 3.3 x 10^11^ GC/mouse 3 weeks before. **(A)** c-Kit-ir cells located near the myenteric plexus transduced by AAV9; **(B)** In the longitudinal muscle layer, not all of the c-Kit-ir elongated cells with processes were aligned parallelly to the projection of AAV9-transduced nerves (arrows); a few varicosities closely located to c-Kit-ir cells (yellow dots marked by arrowheads); **(C)** AAV9-transduced nerve fibers were distributed among c-Kit-ir cells in the base of the mucosal crypts; **(D)** c-Kit cells closely located to the submucosal plexus. Scales as indicated in each image.

**Figure 9:**
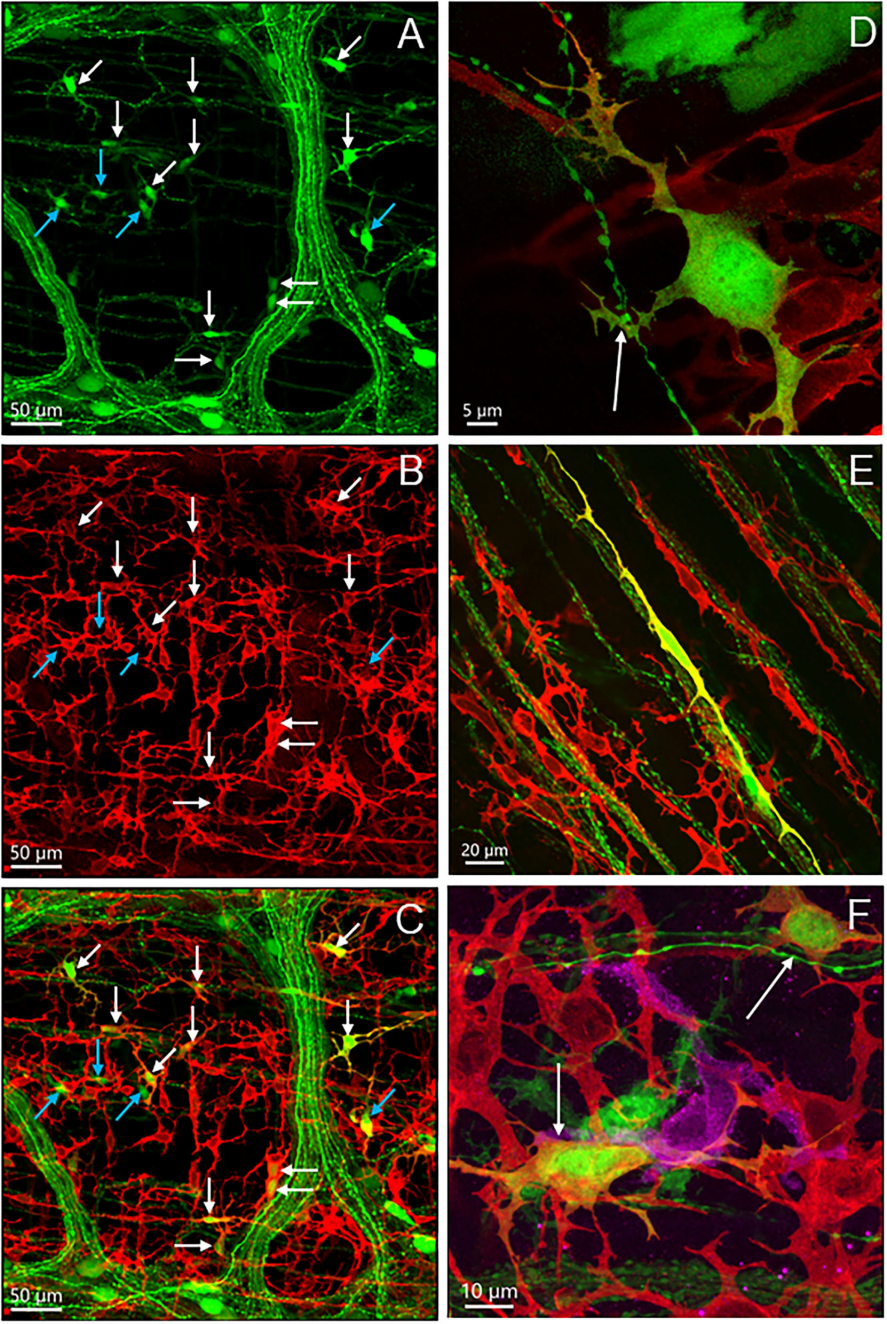
Photomicrographs of c-Kit-ir cells transduced by iv AAV9. **(A–C)** The same photo displayed by separate fluorescence of AAV9 (green) and c-Kit (red), and dual labeling of an area with many AAV9+c-Kit-ir cells (white arrows). Some AAV9-transduced cells with similar morphology were not c-Kit-ir (blue arrows). **(D)** Processes of an AAV9+c-Kit cell closely adjacent to a varicose axon (arrow). **(E)** A long slender dual labeled cells in the circular muscle layer. **(F)** Close proximity of an Iba1-ir macrophage cell (magenta), AAV9+c-kit cells (arrows), and an AAV9 cell in the net of c-kit ICC cells near myenteric plexus.

The mouse colon contained numerous c-Kit-ir ICC in different layers (Figure 8). In the proximal colon, c-Kit-ir ICC were in proximity to several AAV9-transduced neuronal elements, in particular, dense c-Kit-ir cells were nested close to the myenteric plexus (Figure 8A). In the longitudinal muscle layer, not all the varicose axon branches projected in parallel to the c-Kit-ir ICC, and some fibers crossed diagonally a couple of ICC rows (Figure 8B). In the mucosal layer, some c-Kit-ir cells were distributed along varicose axons innervating the crypts in the mucosa layer (Figure 8C) and also near the submucosal ganglia (Figure 8D). Among the abundant c-Kit-ir ICC, a few were noticeable as they contained AAV9 expression (Figure 9 and Supplementary Figure 14). The AAV9/c-Kit cells were located in c-Kit-ir ICC nest around the myenteric ganglia and interganglionic strand, mixed with the neuronal structures in the spaces among the ganglia, and in the circular muscle layer, sometimes along the nerves (Figure 9E and Supplementary Figure 14F). Occasionally, a couple of AAV9/c-Kit cells were observed in the longitudinal ICC, and closely adjacent to varicose nerve fibers (Supplementary Figure 14). The AAV9/c-Kit cells were polymorphic, such as multipolar, bipolar, fusiform, or long slender with a big nucleus surrounded by a thin cytoplasm. Their processes had enlargements and small branches, which were distinguishable from neurons. In each binocular field through a 20 × objective, 0–15 cells were AAV9 transduced. AAV9/c-Kit cells were 71.4 ± 2.1% of AAV9 expressing ICC. The c-kit negative cells with AAV9 transduction have the same morphologies and locations as AAV9/c-Kit cells, and none of them was co-localized with immunoreactivity of Iba1 (Figure 9F) and GFAP (Figure 6).

### Distribution of Nerve Fibers Transduced by AAV-PHP.S-hSyn-tdTomato Farnesylated, and Relationship With Glia, Macrophage and ICC

**Figure 10:**
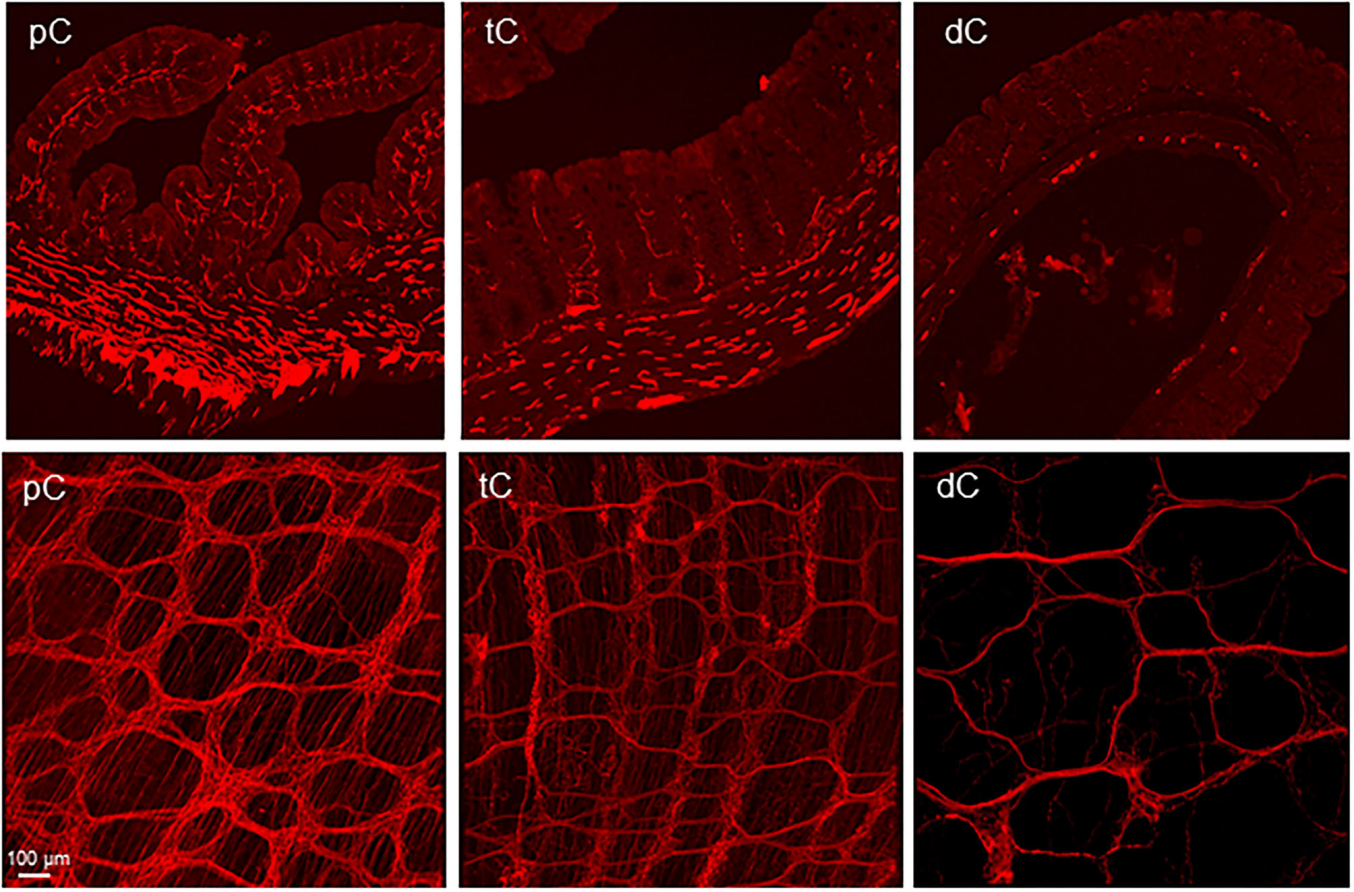
Representative photomicrographs of the transduction by AAV-PHP.S-hSyn1-tdTomato farnesylated (PHP.S-tdTf) in the mouse colon. PHP.S-tdTf (1 x 10^12^ GC/mouse) was retro-orbitally injected 3 weeks before. The AAV-labeled nerves were abundant in the proximal colon (pC). **Upper panel**: transverse sections showing all layers of the colon wall. The iv PHP.S-tdTf-transduced nerve terminal fibers are well-demonstrated in the mucosa, and the nerves in muscular layers are very dense. **Lower panel**: flattened colon wall. The longitudinal axis of all samples is placed along the left-right direction of the photomicrographs. Images show the myenteric plexus and the nerves innervating the circular muscles in the pC and transverse colon (tC). The longitudinal interganglionic strands were labeled in all the segments. Scale: 100 μm same for all panels.

**Figure 11:**
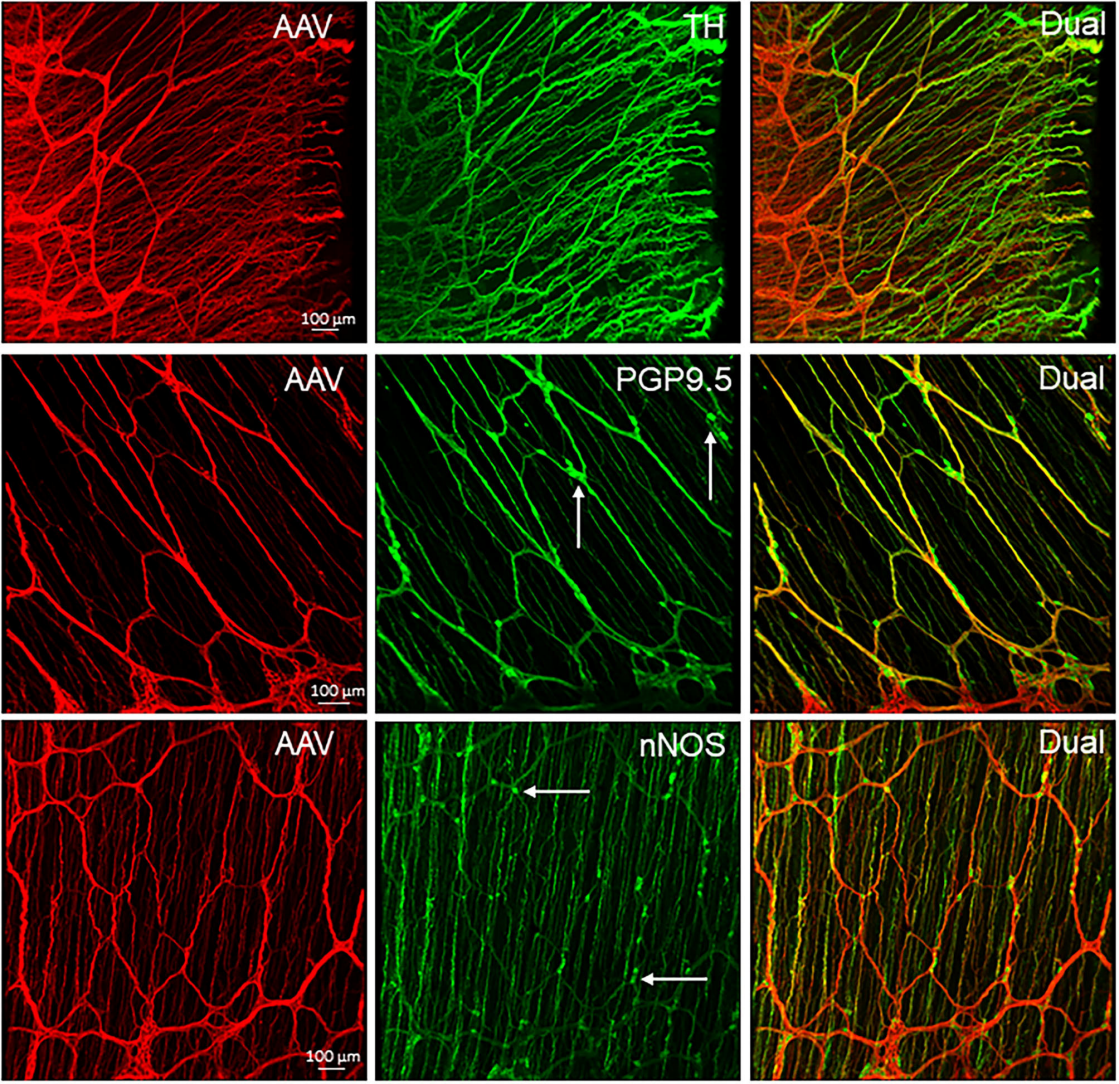
PHP.S-tdTf transduced nerve fibers in the margins of mouse proximal colon combined with immunofluorescent labeling. PHP.S-tdTf (1 x 10^12^ GC/mouse) was retro-orbitally injected 3 weeks before. **Upper row**: the mesenteric margin enriched by nerve fibers, and many dual-labeled by PHP.S-tdTf and TH. **Middle row** and **lower row**: the antimesenteric margin consisted of small circumferential nerve bundles and scattered neurons immunostained by PGP9.5 or nNOS (arrows). Scales 100 μm for each row.

**Figure 12:**
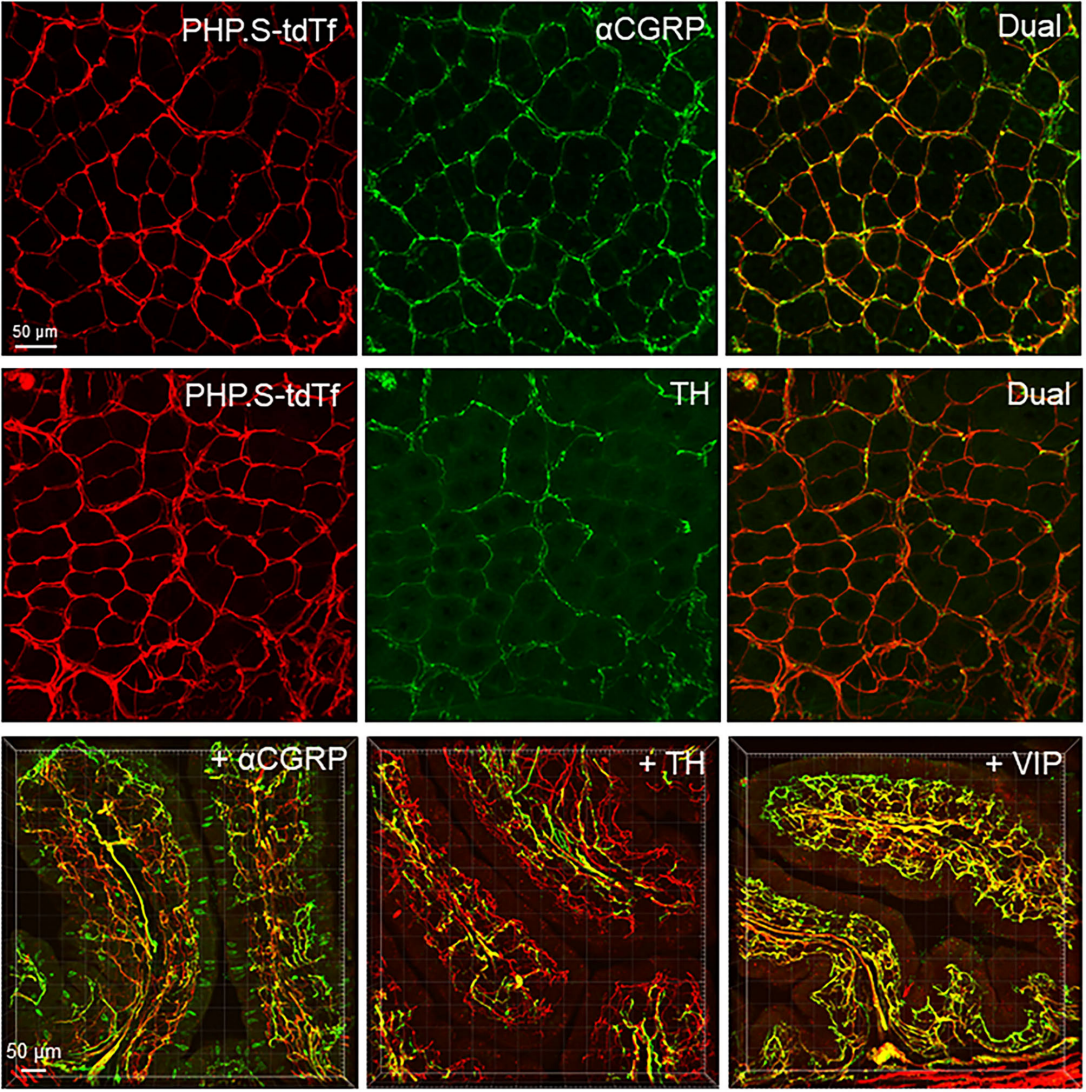
PHP.S-tdTf (red) transduction in the mouse proximal colon mucosa combined with immunofluorescence (green) of calcitonin gene related peptide-alpha (αCGRP), tyrosine hydroxylase (TH), and vasoactive intestinal peptide (VIP). PHP.S-tdTf was injected retro-orbitally at 1 x 10^12^ GC/mouse 3 weeks before. Confocal microscopic images in the upper and middle rows were acquired from a flattened proximal colon wall at the level of the mucosal crypts, and images in the lower row were in trans-vertical sections. TH, αCGRP, and VIP immunofluorescence were found in PHP.S-tdTf transduced nerve fibers (yellow). Scale: 50 μm same for upper and middle rows and 50 μm same for the lower row.

PHP.S-tdTf was used at a dose of 1 × 10^12^ GC/mouse *via* retro-orbital injection to obtain the maximal labeling of nerve fibers as possible. Compared with other segments, the proximal colon contained the most abundant PHP.S-tdTf-transduced nerve fibers, and the labeling decreased from the transverse to the distal colon (Figure 10). In the proximal colon, dense tdTomato fluorescent nerve fibers were located in the submucosal and myenteric plexuses, the interganglionic strands, along the circular muscles and in the mucosa (Figure 10, Supplementary Figure 15). Similar to the AAV9 labeling, the longitudinal interganglionic strands in the myenteric plexus were prominent, while the circumferential strands were not well-formed (Figure 10, Supplementary Figure 15). The circumferential nerve fibers were dense in the mesenteric margin (Figure 11). Compared to the main zone, the myenteric plexuses in both margins contained less and smaller ganglia or just a couple of scattered neurons as shown by combined immunostaining (Figure 11 and Supplementary Figure 16). The longitudinally running nerve fibers were sparsely and lightly labeled in the layer under the serosa (data not shown). At the level of mucosal crypts, labeled fibers were connected in a shape like honeycomb (Figure 12). In the distal colon, some longitudinal interganglionic strands were labeled, but a few nerve fibers were found in the myenteric ganglia (Figure 10 dC in the lower row).

**Figure 13:**
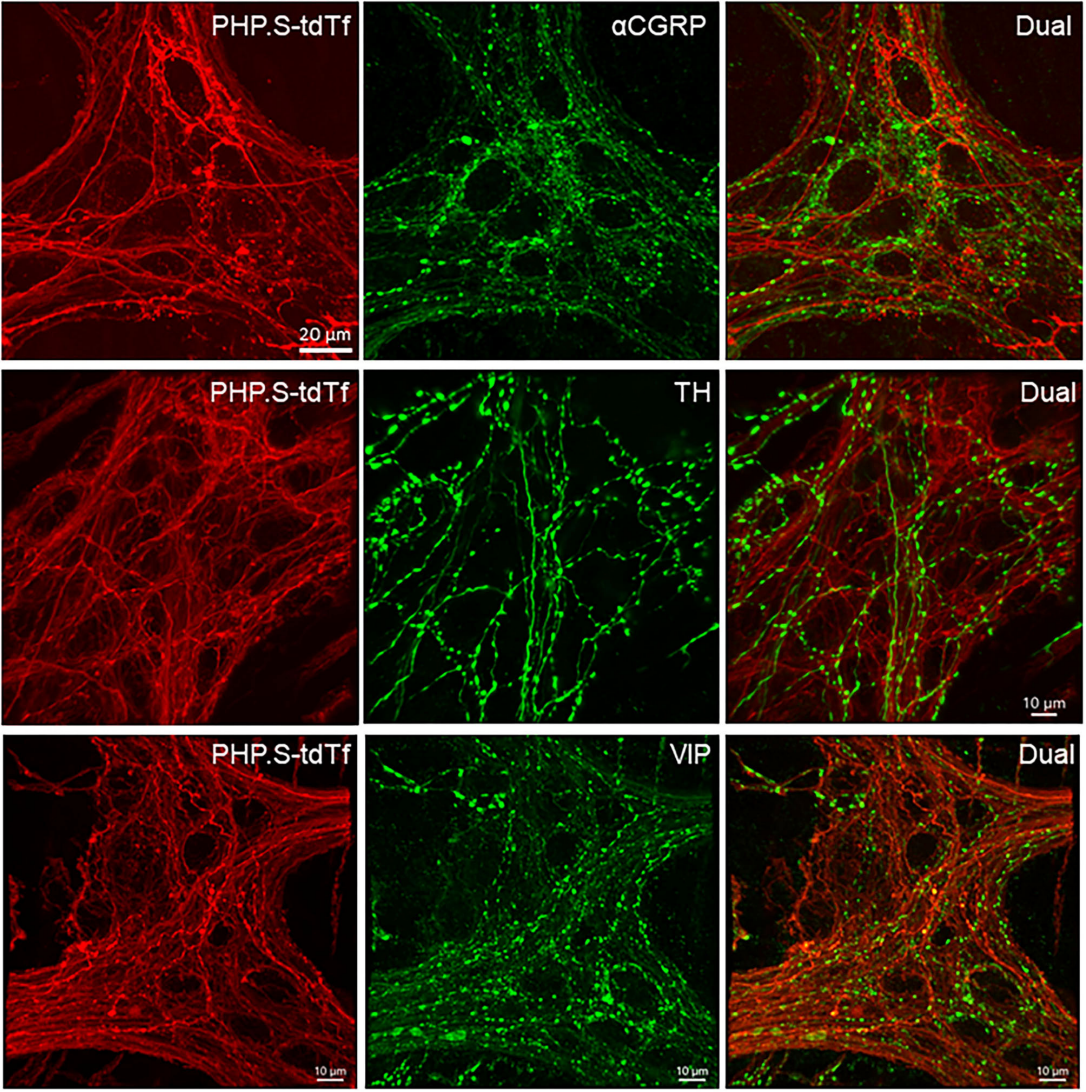
Photomicrographs at high magnification of PHP.S-tdTf transduction in the myenteric plexus of the mouse proximal colon combined with immunofluorescence of calcitonin gene related peptide-alpha (α-CGRP), tyrosine hydroxylase (TH), and vasoactive intestinal peptide (VIP). Few co-localizations of AAV-tdTf with αCGRP, TH-ir, or VIP-ir were found. Scale bar 20 μm, same for all images.

In the flattened proximal colon with the whole thickness treated with passive CLARITY or 2%TPBS, combined with immunohistochemistry for nerve fiber markers, αCGRP-, TH- and VIP-labeling was observed in the mucosa, submucosa, and muscular layers. However, in the myenteric ganglia, few immunofluorescences co-existed with PHP.S-tdTf in the main zones where nerve fibers contained dense and intense tdTomato fluorescence (Figure 13). Enteric neurons of the main zones bearing dense PHP.S-tdTf labeling showed little immunoreactivity of the neuronal markers, PGP9.5 and nNOS (Supplementary Figure 17), which can be observed in the margins (Figure 11). The labeling in the submucosal plexus improved in the mucosa removed samples (Supplementary Figure 18). In the mucosa of both the flat colonic wall and transverse sections, double labeling with immunofluorescence of α-CGRP, TH or VIP was shown in the nerve fibers (Figure 12, lower panels).

GFAP-ir glial cells were close to but not colocalized with PHP.S-tdTf expression in the submucosal and myenteric ganglia (Supplementary Figure 19).

**Figure 14:**
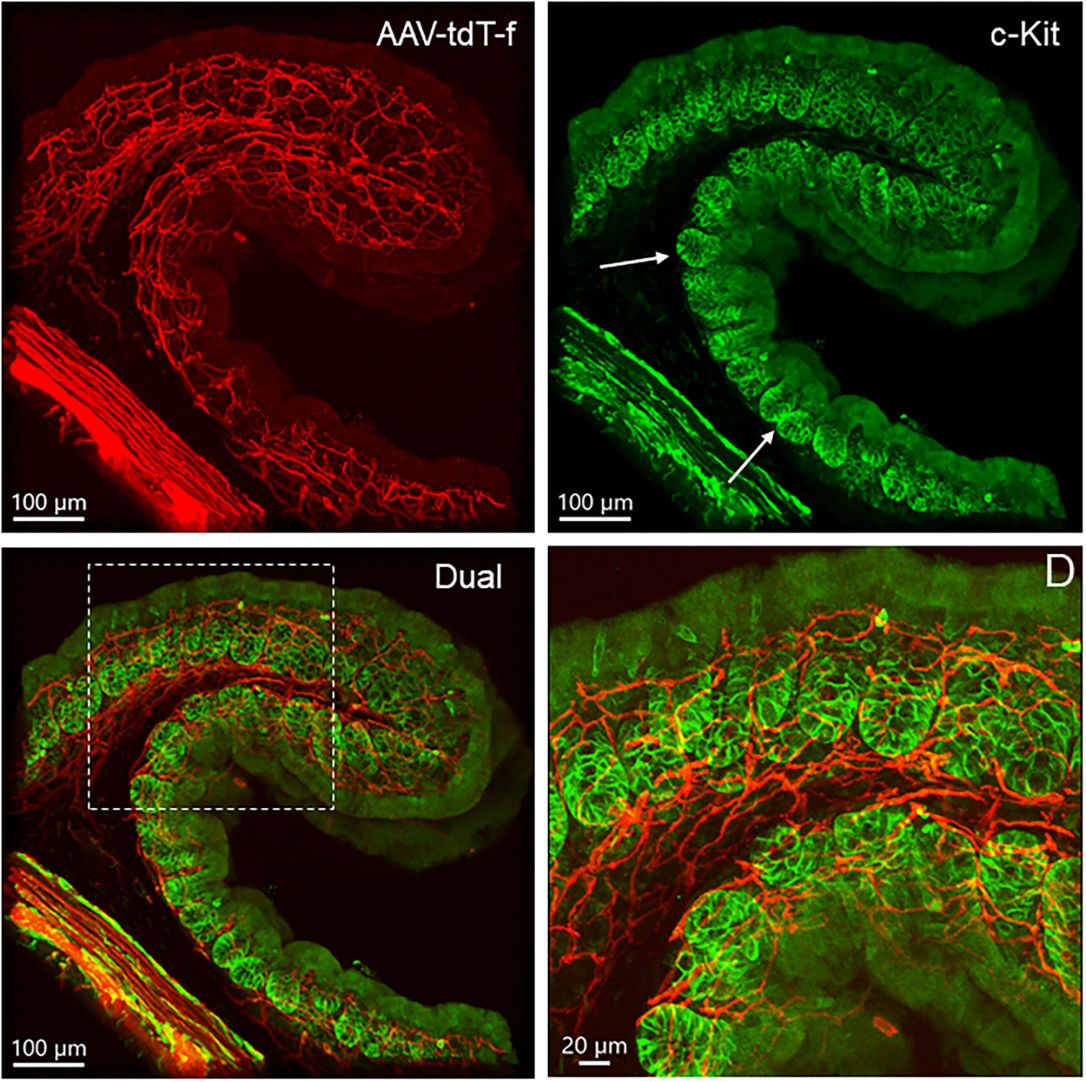
Dual labeling of PHP.S-tdTf (red) and c-Kit (green) show locations of c-Kit-immunoreactive cells in relationship with nerve fibers in transverse sections of the mouse proximal colon. Cells in the mucosa crypt base contained c-Kit immunoreactivity as indicated by arrows, and the area in the dash line is magnified in D.

ICC with c-Kit immunofluorescence in the proximal colon were located closely to PHP.S-tdTf transduced nerves in the longitudinal and circular muscle layers, and nested around myenteric ganglia (Supplementary Figure 20). The mucosal c-Kit-ir without process were found in the crypts (Figure 14).

Distribution and morphologies of Iba1-ir macrophages were similar in PHP.S-tdTf transduced mouse colon as in AAV9 (data not shown). In the myenteric plexus, many Iba1-ir cells were located in the spaces among the ganglia, inside the ganglia, along nerve fibers, and in the submucosa (Supplementary Figure 21).

### AAV-Transduction in the Other Parts of the Gastrointestinal Tract

The systemic injection of AAV9 (Addgene) transduced enteric neurons and nerve fibers also in the stomach and small intestine with regional selectivity (Supplementary Figure 22). The lower esophagus did not show neuronal labeling in the enteric plexuses, a few myenteric ganglia appeared in the gastric corpus, while the myenteric ganglia were brightly labeled the gastric antrum near the pylori. In the small intestine, myenteric ganglia were transduced more prominently in the ileum than the duodenum and jejunum (Supplementary Figure 22), and in the submucosa ganglia, the transduction occurred more in the upper segments compared with the ileum (Supplementary Figure 23). The gastric antrum and small intestine contained many AAV-labeled cells; however, their morphologies were not identical to those ICC found in the proximal colon (Supplementary Figure 24). The cells were not polymorphic as those in the proximal colon, and most of them were multipolar forming dense clusters in the duodenum and jejunum. Immunofluorescent labeling displayed that the non-neuronal cells in the small intestine were also c-Kit positive (Supplementary Figure 25). The AAV9-transduced cells in the esophagus were smaller and had shorter processes compared with those in the small intestine (Supplementary Figure 24).

Nerve fibers labeled with PHP.S-tdTf fluorescence were also shown in the other parts of GI (Supplementary Figures 26, 27) with few in the esophagus. In the gastric corpus, weak tdTomato fluorescence was found in the enteric plexus with a few thick nerve endings of which many are located in the circular muscles. By contrast, the myenteric plexus in the antrum was more densely labeled compared to the gastric corpus (Supplementary Figure 27). The ileal myenteric plexus was denser than that in the duodenum and jejunum, since the ganglia contained more neurons compared to the oral segments.

**Figure 15:**
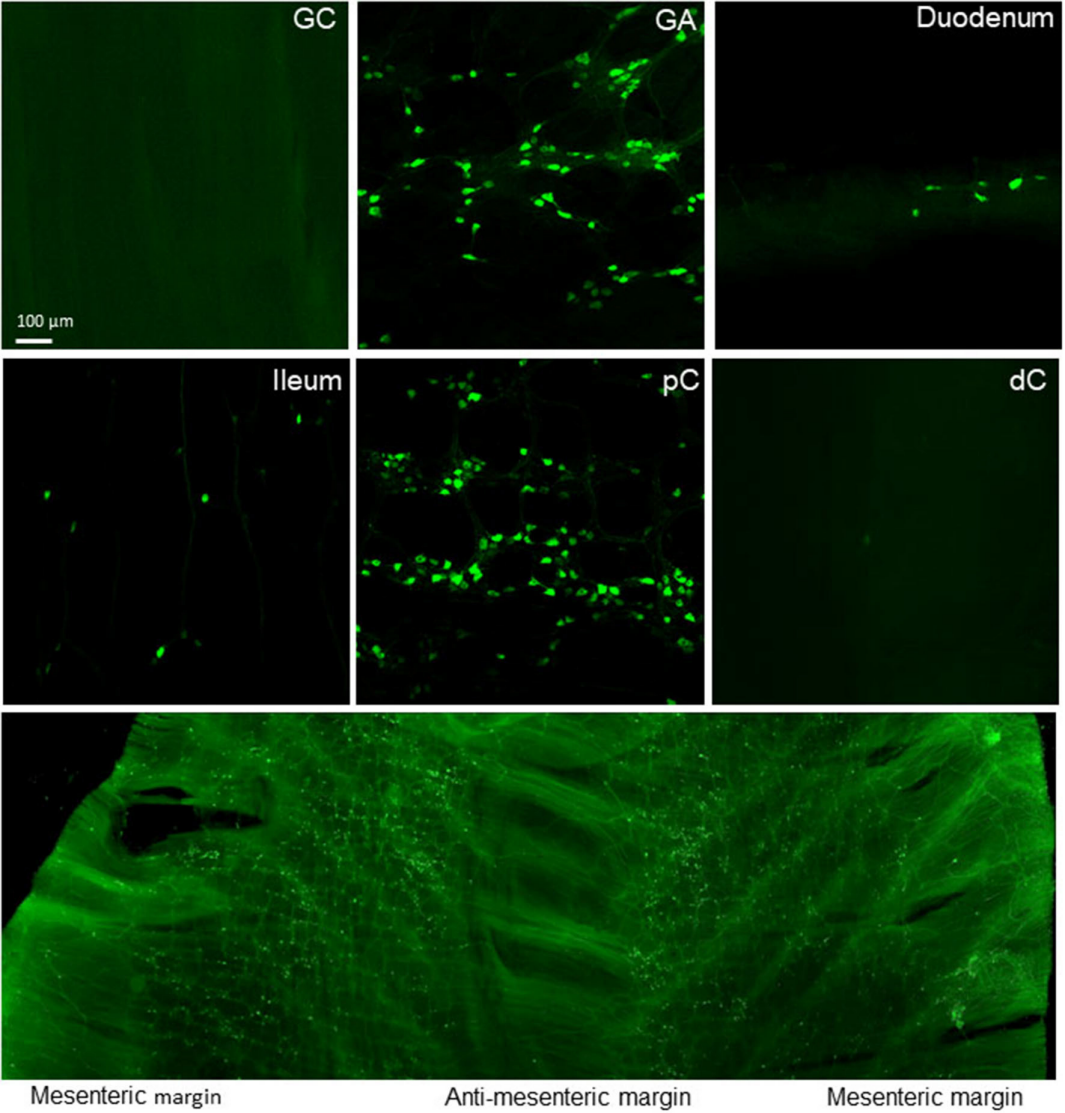
GI mapping in nNOS-Cre mice. AAV-PHP.S-CAG-DIO-EYFP was retro-orbitally injected at injected at 3.3 x 10^11^ GC/mouse 3 weeks before. The lower panel show unevenly distributed iv AAV-transduced nNOS neurons in the proximal colon.

Beside AAV9 from Addgene, the other variants of AAV9 and AAV-PHP tested (Table 1) showed similar distribution pattern of AAV-transduced cells in the GI tract in wide type mice (Supplementary Figure 28) and nNOS- and ChAT-Cre mice (Figure 15 and Supplementary Figure 29). Particularly, only a few in the distal colon and gastric corpus and uneven distribution in the myenteric ganglia of the proximal colon.

### AAV-Transduction in the Sensory and Autonomic Ganglia

The nodose and dorsal root ganglia at the levels L1 and L6 innervating the colon contained many AAV-transduced neurons, so did the pelvic ganglia. Only a few neurons were transduced in the celiac-superior mesenteric ganglion (Supplementary Figure 30).

### Technical Notes

We found that neurons and cells were not well-identified by immunofluorescent labeling in the main zone of the whole thickness of the proximal colon wall using passive CLARITY, including antibodies for ChAT, nNOS, pan-neuronal markers (HuC/D, NeuN and PGP9.5), GFAP and c-Kit (example in Supplementary Figure 17). Treatment with 2% Triton-PBS to the colon whole wall improved a little, showing some patches of immunoreactive cells (data not shown). The immunofluorescence was bright in the submucosal plexus of samples with mucosa scratched off and mucosal-submucosal whole mount preparations (Supplementary Figures 18, 19). However, circular muscle bundles remained on the myenteric plexus-longitudinal muscles layer could prevent the antibodies from penetration in whole mount preparations (Supplementary Figure 31).

Occasionally, some AAV-transduced neurons in the colon showed uncommon morphology (Supplementary Figure 32). Whether they were normal or artifacts could not be ascertained.

## Discussion

Systemic administration of AAV9 vectors in adult C57BL/6J mice resulted in segmental difference of transduction in the adult mouse colon. The proximal colon contained abundant AAV-transduced neurons and nerve fibers while the distal colon showed almost no neurons but scattered small bundles of nerve fibers and terminals. The AAV9-transduced neurons were unevenly distributed in the myenteric plexus. Labeling by AAV9 engineered variants and in ChAT-Cre and nNOS-Cre mice showed a similar distribution pattern, indicating that this segmental difference in the colon is not related to a specific AAV vector or mouse strain. In addition, it is not specific to the colon since other parts of GI tract such as the stomach showed the striking difference in the transduction between the antrum and corpus. In the proximal colon, AAV9-transduced cells in the myenteric plexus did not show sex differences and encompassed neurons with immunofluorescence for ChAT, nNOS, and calbindin, and in the submucosal plexus VIP. Another unexpected finding in the proximal colon was the occurrence a few scattered c-Kit-ir cells containing AAV9 among dense ICC networks, which suggests that some ICC respond to systemic AAV and may play a role in communication among systemic signals and cells in the colon.

### AAV Transduced Neurons and Nerve Fibers

Both AAV9 and AAV-PHP.S serotypes transduced neuronal structures in the adult mouse colon with distinct segmental (proximal vs. distal) and regional differences in myenteric ganglia, and similar labeling in both male and female adult mice. The colonic proximal and distal segments have different mucosal morphologies, and density and characteristics of enteric neurons linked to different functions including the facilitation of the absorption of excess fluid and electrolytes, mixing, storage of liquid feces, and fermentation of food residues, taking place in the proximal segment. There are distinct motor pattern of activity and motor function in the proximal and distal colon (Zygulska et al., 2018; Mogilevski et al., 2019; Schiller et al., 2021; Nestor-Kalinoski et al., 2022). The complex function of the proximal colon is apparent by higher neuronal density and more intricate circuitry in the myenteric plexus vs. the distal colon (Smith and Koh, 2017; Li et al., 2019). The segmental differences in the colon were also demonstrated by electrophysiological activity and neurochemical profiles (Takahashi and Owyang, 1998; Sibaev et al., 2003). The proportion of nitrergic neurons was found to be slightly higher in the myenteric plexus of the distal as compared to the proximal colon, while the cholinergic neurons showed no difference in mice (Li et al., 2019). Calcium imaging also indicated that more myenteric neurons responded to focal stimulation in the proximal than the distal colon (Li et al., 2019). However, the knowledge to date provides no explanation for such a striking disparity in systemic AAV transduction between the proximal and distal colon of adult mice, as well as the similar difference found between the gastric corpus and antrum. The extrinsic innervations may not play a role, although their origins are different in the proximal and distal colon, since the gastric corpus and esophagus which are richly innervated by the vagal nerves compared to the proximal colon (Berthoud et al., 1990; Powley et al., 2019), had poor transduction in the enteric nervous system. It is unlikely to be related to the tropism of the vectors tested because all the vectors showed similar pattern. Also, the segmental difference in the efficiency of AAV transduction is independent of the promotors and fluorescent proteins since the AAV9 and engineered variants used had different constructs.

In contrast, previous reports showed that in neonatal and 21 days old mice with AAV8 and AAV9 injected iv at similar doses labeled neurons from the stomach to distal colon, and the distribution in the colon had no significant oral-aboral difference mapped in 9 segments of the colon, 1 cm each (Gombash et al., 2014). There was a higher AAV9 transduction in the HuC/D-ir colonic myenteric neurons of neonatal (~44%) and juvenile (~47%) PVB mice (no difference between proximal and distal colon) (Gombash et al., 2014), compared to 30% in the proximal colon of C57BL/6 mice (present study). Also, in the neonatal guinea pigs, AAV8- and AAV9-transduced neurons did not show differences in oral-aboral distribution in the colon, and from the esophagus to the colon (Gombash et al., 2017). About 22% HuC/D-labeled myenteric neurons contained AAV9 expression in the distal colon of young mice (Buckinx et al., 2016), whereas almost none in the adult mice was found in our study. In the brain and spinal cord, iv AAV9 transduced neurons in newborn and young animals, while in adult mice, there was limited neuronal but robust transduction in astrocytes (Foust et al., 2009). The blood-brain-barrier was considered the factor underlying the difference (Foust et al., 2009; Bevan et al., 2011; Samaranch et al., 2012). However, it might not be one of the mechanisms involved in the different AAV9 transduction in young and adult mice, because the difference in the AAV transduction between the proximal and distal colon does not support that a “blood-enteric neuronal barrier” plays a role. The elucidation of underlying mechanisms involved in a such striking segmental differences between the adult and young mice, require further investigations.

In myenteric ganglia, the uneven distribution of AAV9-transduced neurons is indicative of distinct responsiveness of some neurons to circulating viral vectors. It also suggests that the myenteric ganglia could be differently grouped in a regional dependent manner in the plexus. However, there is no evidence that there was a selectivity of AAV transduction by morphological subtypes of the enteric neurons in the mouse colon, because the majority of AAV9 transduced neurons in various shapes and sizes did not show the fluorescent signal in their processes. Among the AAV9-transduced myenteric neurons, one type was distinct from the others since they had a large soma, a long axon and with or without irregular-shaped dendrites, which could belong to subclasses of Dogiel I neurons (Brehmer et al., 1999). In groups by immunolabeling, AAV9+ChAT double labeled neurons had the highest rate by 50%, AAV9+nNOS 28%, and AAV9+Calbindin 31%, which indicates a prominence of cholinergic neurons transduced by AAV9.

The distributions of AAV-transduced nerve fibers in the mesenteric and antimesenteric margins of the proximal colon were mainly in circular nerve fibers and bundles and none or sparsely in small myenteric ganglia. The combined immunolabeling showed scattered single or a couple of neurons containing neuronal markers and neurotransmitters including ChAT, nNOS, and calbindin. Likewise, a previous study delineated the lack of longitudinal muscles, and less myenteric neurons in the two margins of the mouse proximal colon and named them as hypoganglionic regions (Sibaev et al., 2003). Therefore, the mouse colonic margins are not equivalent to the human colonic taenia comprising a strong band of longitudinal muscles (Hanani et al., 2012).

Systemic AAV9 transduced nerve fibers in the mouse colon might include both the extrinsic and intrinsic nerves. This is supported by the observations that circumferential nerve fibers were dense in the mesenteric margin which could be extrinsic ones running into or from the enteric plexus and circular muscle layers and/or intrinsic axons of myenteric neurons projecting longitudinally and circumferentially cross the myenteric ganglia. In addition, indirect evidence of iv injected AAV9 transduced neurons in the sensory and autonomic ganglia could be the sources of the extrinsic nerves to the colon.

There were the longitudinal interganglionic projections which were prominent. Some of the myenteric neuronal axons project a long distance as reported in the mouse colon by iv injection of AAV2/9 (Li et al., 2019) or AAV-PHP.S (Chan et al., 2017), in the rat colon by intracolonic injection of AAV9 (Benskey et al., 2015) and in the small intestine by DiI tracing in guinea pigs (Brookes et al., 1995).

### Relationship to Non-neuronal Cells

Neurons crosstalk with non-neuronal cells such as glia (Alvarez-Maubecin et al., 2000; Iruzubieta et al., 2020), ICCs (Vannucchi, 1999; Iruzubieta et al., 2020), and immune cells (Muller et al., 2020; Jacobson et al., 2021). The AAV-labeling has advantage to elucidate innervation to non-neuronal cells.

Glial cells labeled with GFAP immunoreactivity are densely distributed in the ganglia and closely around the AAV9-labeled neurons and nerve fibers. Some AAVs can target glial cells (Foust et al., 2009), however, the absence of double labeling with GFAP excluded that AAV9 can transduce enteric glial cells in the adult mouse colon. Likewise, in other reports GFAP- and S100b-containing cells in the colon did not have AAV9 transduction (Gombash et al., 2014; Buckinx et al., 2016). We did not use S100b to label the enteric glia taking into the consideration that S100b antibody labels other types of cells, such as certain dendritic cells, lymphocytes, Schwann cells, Langerhans cells, melanocytes, and etc. (Sugimura et al., 1989; Uchida and Endo, 1989; Donato et al., 2013; Zeng et al., 2019; Su et al., 2021).

The AAV9 vector with a pan-promotor transduced a few non-neuronal cells in the proximal colon, and the majority of them were c-Kit-ir. ICC are well-established to play a role as pacemaker cells in the colonic motility and contain c-Kit (Vannucchi, 1999; Pasternak et al., 2016; Iino et al., 2020). The c-Kit-ir cells in the mouse proximal colon were widespread and abundant as previously reported (Ward et al., 1997; Vanderwinden et al., 2000). However, AAV9/c-Kit ICC were sparse and embedded in a dense c-Kit-ir ICC network among myenteric plexus or along nerves in muscle layers. The AAV9/c-Kit ICC could be a part of ICC network that was identified playing a role in GI transit (Takaki, 2003; Blair et al., 2012; Sanders et al., 2014; Kishi et al., 2020). We also visualized the c-Kit-ir cells at the base of mucosal crypts which have been rarely reported before. They might be progenitor cells (Xue et al., 2018) because c-Kit is also considered as a stem cell factor (Yavuz et al., 2002). Some AAV9-transduced cells with the same morphologies were c-Kit negative. They were not immunostained by Iba1 and GFAP, suggesting they may not be macrophage or glia. Whether they belong to any other cell group, such as platelet-derived growth factor receptor alpha (PDGFRα)-positive cells (Blair et al., 2014) will need to be identified.

It is documented that ICC in different intestinal layers have distinct morphologies and functions (Vannucchi, 1999; Al-Shboul, 2013; Pasternak et al., 2016), and their levels decreased with aging, inflammatory diseases, and constipation (Huizinga and Chen, 2014). Some ICC are located between autonomic nerve endings and smooth muscular cells to form connections with extrinsic neurons (Vannucchi, 1999; Blair et al., 2012; Al-Shboul, 2013; Huizinga and Chen, 2014) and surround enteric ganglia and nerve tracts in human myenteric plexus as visualized by electromicroscopy and 3D reconstruction (Iruzubieta et al., 2020). The evidence of some ICC contact nerves, macrophages and smooth muscle cells (Schneider et al., 2019; Iruzubieta et al., 2020) supports that there is a subclass of ICC playing a role in communication among different types of cells.

Quantitative comparison of the volume of macrophages labeled by Iba1 did not show difference in the proximal colon between naïve and AAV9-tranduced mice, indicating no inflammatory response to AAV9 as reported before (Gombash et al., 2014; Benskey et al., 2015). However, the close proximity of Iba1-ir macrophages to AAV9-transduced neurons and engulfed nerve fibers suggest that some immune responses could occur.

### Technical Considerations

The PHP.S-tdTf vector did not label all nerves in the colon, although farnesylation of fluorescent protein enhances membrane binding and the AAV vector has a neuronal promoter. This statement is supported by that PHP.S-tdTf did not transduce all varicose fibers, since immunofluorescent labeling of nerve fibers by αCGRP and TH mostly do not co-localized with the tdTomato signals in the myenteric ganglia.

AAVs are useful vectors for therapeutic purpose by delivering genes and drug and for functional studies (Haggerty et al., 2020). Although they were used in some studies as neuronal tracers (Wouterlood et al., 2014; Rao and Wang, 2020; Luchicchi et al., 2021) and local injection in the mouse brain revealed Golgi-like detail (Watakabe et al., 2014; Lin et al., 2018), our data demonstrated that systemically administered AAVs do not have the capacity to define fine morphological profiling of enteric nervous system. In addition, many enteric neurons did not contain AAV in their processes and AAV transduction weakened immunolabeling neurons and nerve fibers, which is an obstacle to characterize the neurochemistry.

However, the use of AAV-labeled neural structures combined with immunolabeling allowed us to reveal organization patterns, and segmental and regional differences of the mouse enteric plexuses. For instance, the myenteric ganglia in the mesenteric and antimesenteric margins are much less and smaller than in the main zone. The circumferential nerve fibers often do not form the interganglionic strands as the longitudinal ones, since the myenteric ganglia are often located closely in chains along the circumferential direction. More importantly, the data can trigger investigations on why and how the proximal colon responds preferentially to the systemic AAV in the adult mice and some myenteric ganglia contained many transfected neurons while others do not.

## Conclusion

The results demonstrate the segmental and regional differences of neurons and nerve fibers transduced by systemic delivery of AAV9 and its modified types in the adult mouse colon, contrasting with previous reports using delivery in neonatal and juvenile mice. The segmental difference with a sharp decrease in transduction in the distal colon compared to proximal colon was also observed in other portions of the GI tract, such as the prominent transduction in the gastric antrum vs. the corpus. In the proximal colon, the transduction was higher in cholinergic than inhibitory (nNOS) or sensory (calbindin) myenteric neurons, while there was no transduction in enteric glia. We also found a small population of c-Kit positive ICC were transfected by AAV9. Their locations in the ICC network and along the nerves may indicate an important role, which warrants further investigations. These data are valuable for gene therapy and functional studies, and may lead to investigation on the distinct characteristics of neurons in the different GI portions. In addition, these findings point to new studies to delineate underlying mechanisms involved in such distinct GI segmental transduction in adult mice. Systemic applications of AAV vectors have limitation for neuronal tracing in the GI, as the labeling of neurons did not delineate morphological details and networks of the enteric nervous system, and did not differentiate extrinsic and intrinsic innervations.

## Data Availability Statement

The raw data supporting the conclusions of this article will be made available by the authors, without undue reservation.

## Ethics Statement

The animal study was reviewed and approved by Animal Research Committee at Veterans Affairs Greater Los Angeles Healthcare System.

## Author Contributions

LW and YT initiated the studies and contributed to the conception, study design, and writing the manuscript. LW conducted the experiments, acquired and segmented the images, analyzed the data, and prepared the figures. CC and SR contributed the AAV-PHP.S vectors production. CC edited the manuscript critically. P-QY participated in data interpretation and edited the manuscript. All authors contributed to the article and approved the submitted version.

## Funding

This work was supported by NIH SPARC award OT2OD24899, Department of Veterans Affair Rehabilitation Research and Development grant 1RX001685, Veterans Administration Senior Research Career Scientist Award, P30 NIHDDK-41301 CURE: Digestive Diseases Research Core Center Grant.

## Conflict of Interest

The authors declare that the research was conducted in the absence of any commercial or financial relationships that could be construed as a potential conflict of interest.

## Publisher's Note

All claims expressed in this article are solely those of the authors and do not necessarily represent those of their affiliated organizations, or those of the publisher, the editors and the reviewers. Any product that may be evaluated in this article, or claim that may be made by its manufacturer, is not guaranteed or endorsed by the publisher.
