# Supplementary material for: Transduction of Systemically Administered Adeno-Associated Virus in the Colonic Enteric Nervous System and c-Kit Cells of Adult Mice
Source: Front Neuroanat. 2022 Jun 6;16:884280. doi: 10.3389/fnana.2022.884280 (PMC9207206; doi:10.3389/fnana.2022.884280)
Supplement: Supplementary file 1 [file Presentation_1.pdf]

Supplementary Figures

Transduction of systemically administered adeno-associated virus in the colonic enteric nervous system and c-Kit cells of adult mice

Lixin Wang, Pu-Qing Yuan, Collin Challis, Sripriya Ravindra Kumar, and Yvette Taché

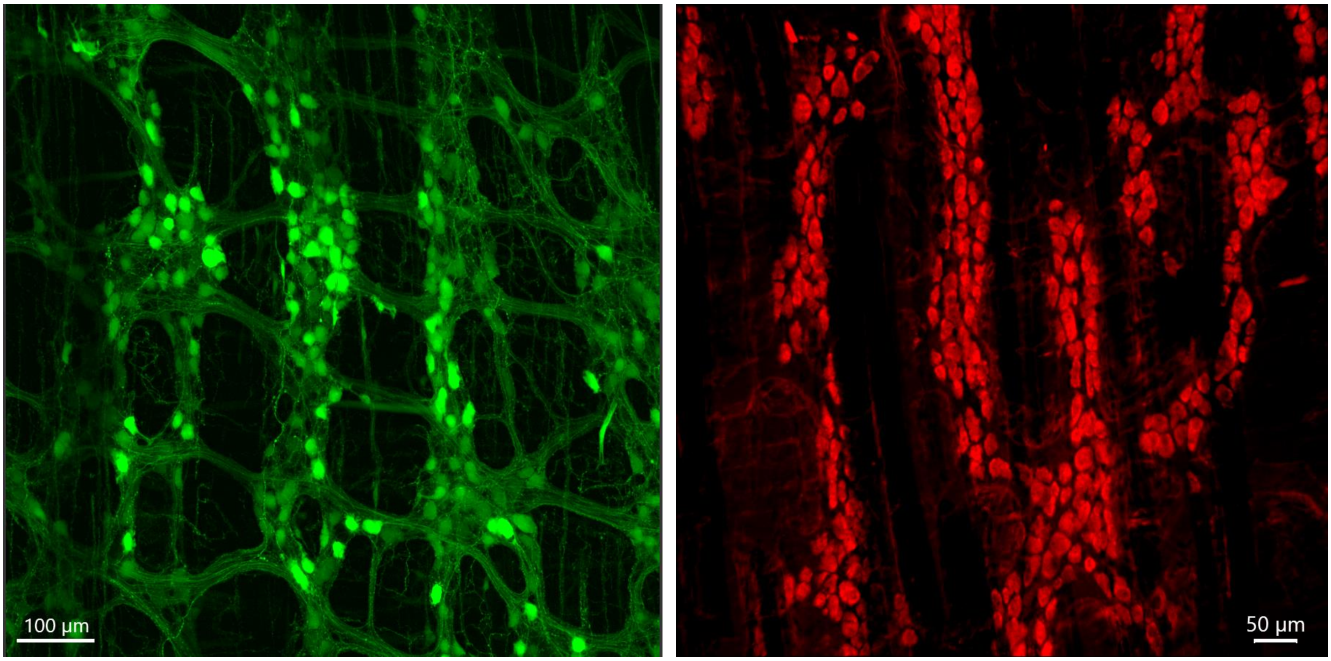

Supplementary Figure 1. Some myenteric ganglia merged circumferentially without obvious interganglionic strands in the mouse proximal colon. Left: AAV9 transduced neurons and nerve fibers. Right: HuC/D pan-neuronal immunoreactivity.

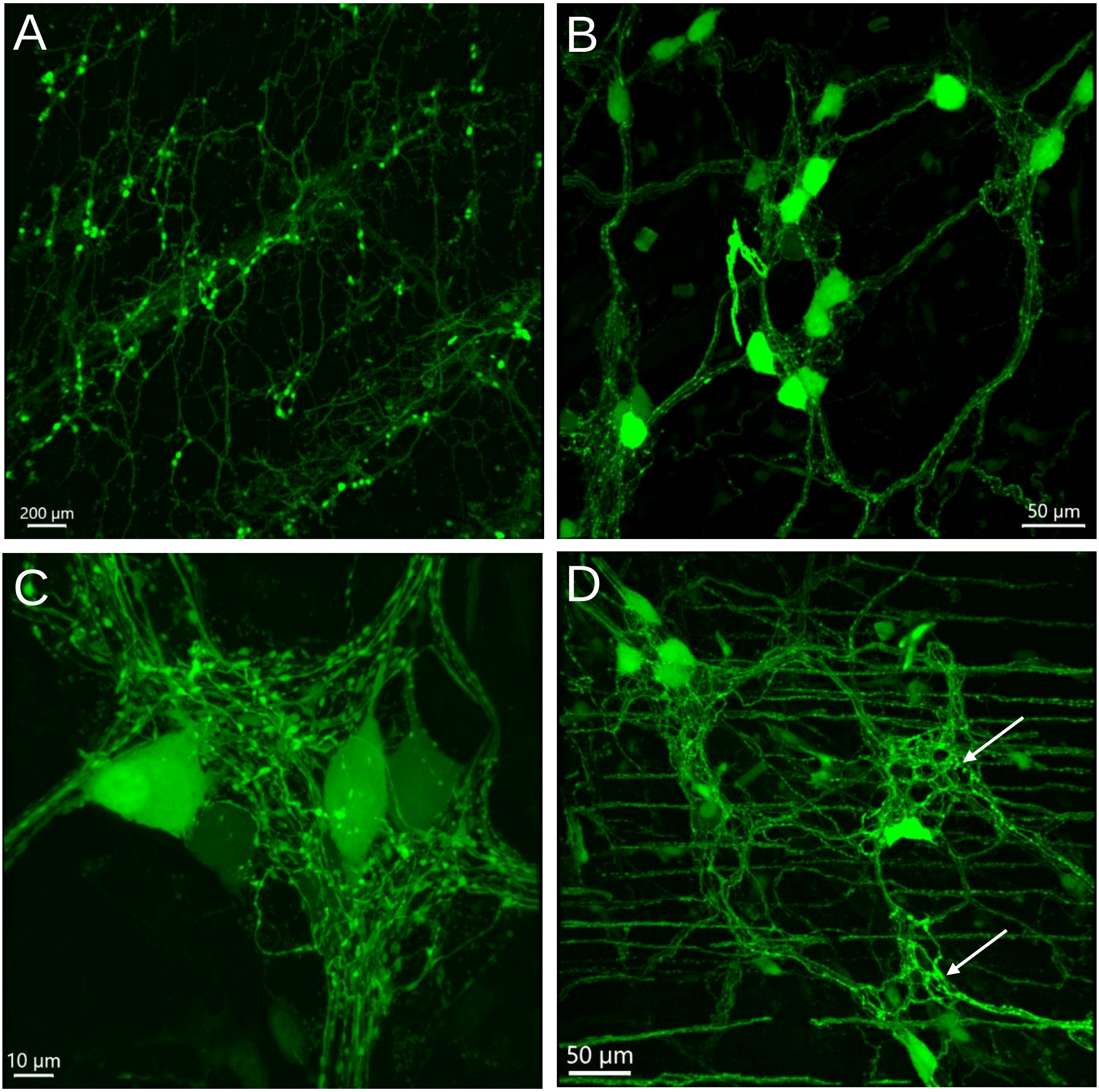

Supplementary Figure 2. AAV9 transduction in the submucosal plexus of the mouse proximal colon. **A.** many neurons were distributed along the mucosal folds. **B.** a part of the submucosal plexus in an area near antimesenteric margin between the ends of mucosal folds. **C.** a submucosal ganglion contained many nerve fibers and endings. **D.** intraganglionic varicose nerve fibers. Scale as indicated in each image.

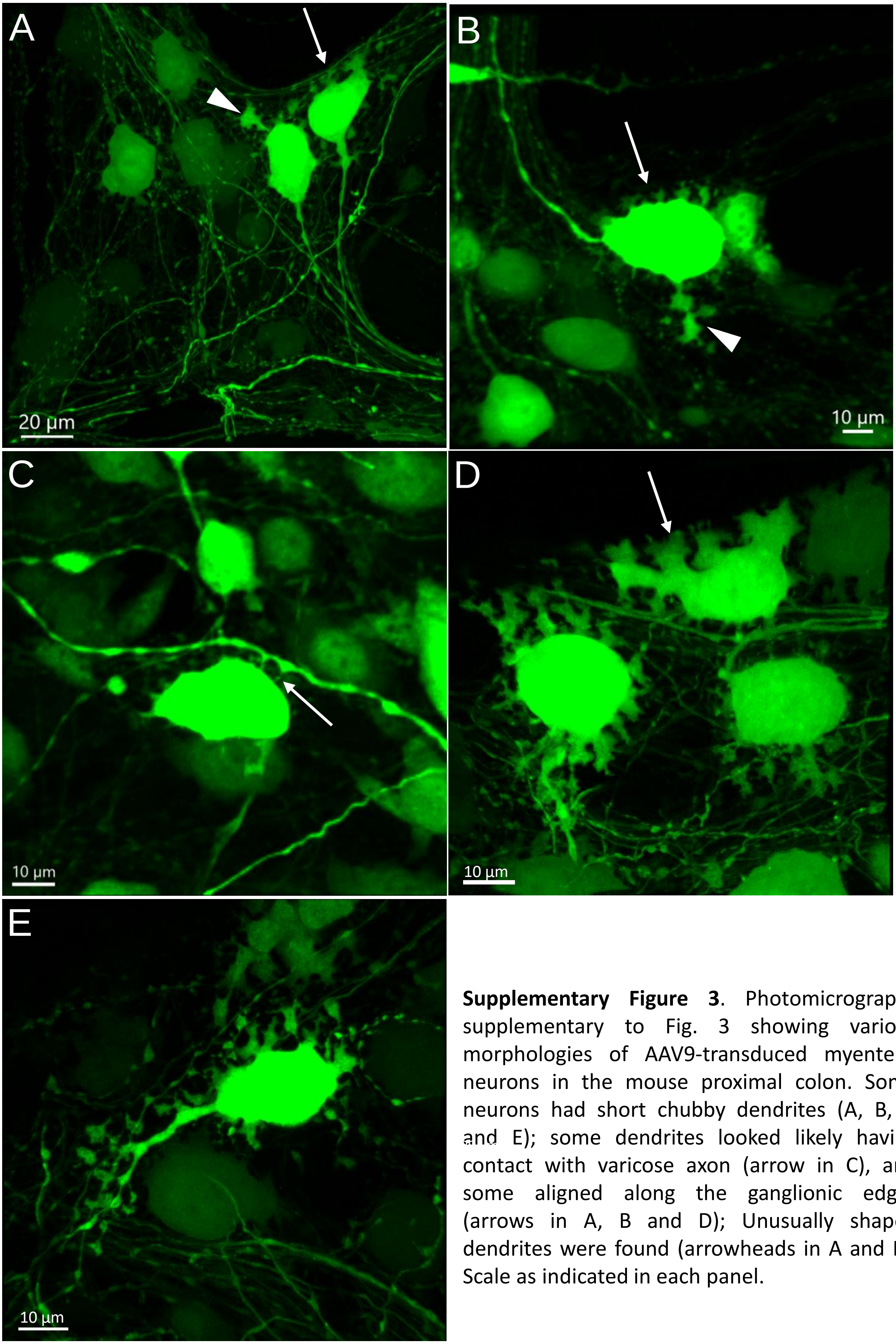

Supplementary Figure 3. Photomicrographs supplementary to Fig. 3 showing various morphologies of AAV9-transduced myenteric neurons in the mouse proximal colon. Some neurons had short chubby dendrites (A, B, D and E); some dendrites looked likely having contact with varicose axon (arrow in C), and some aligned along the ganglionic edges (arrows in A, B and D); Unusually shaped dendrites were found (arrowheads in A and B). Scale as indicated in each panel.

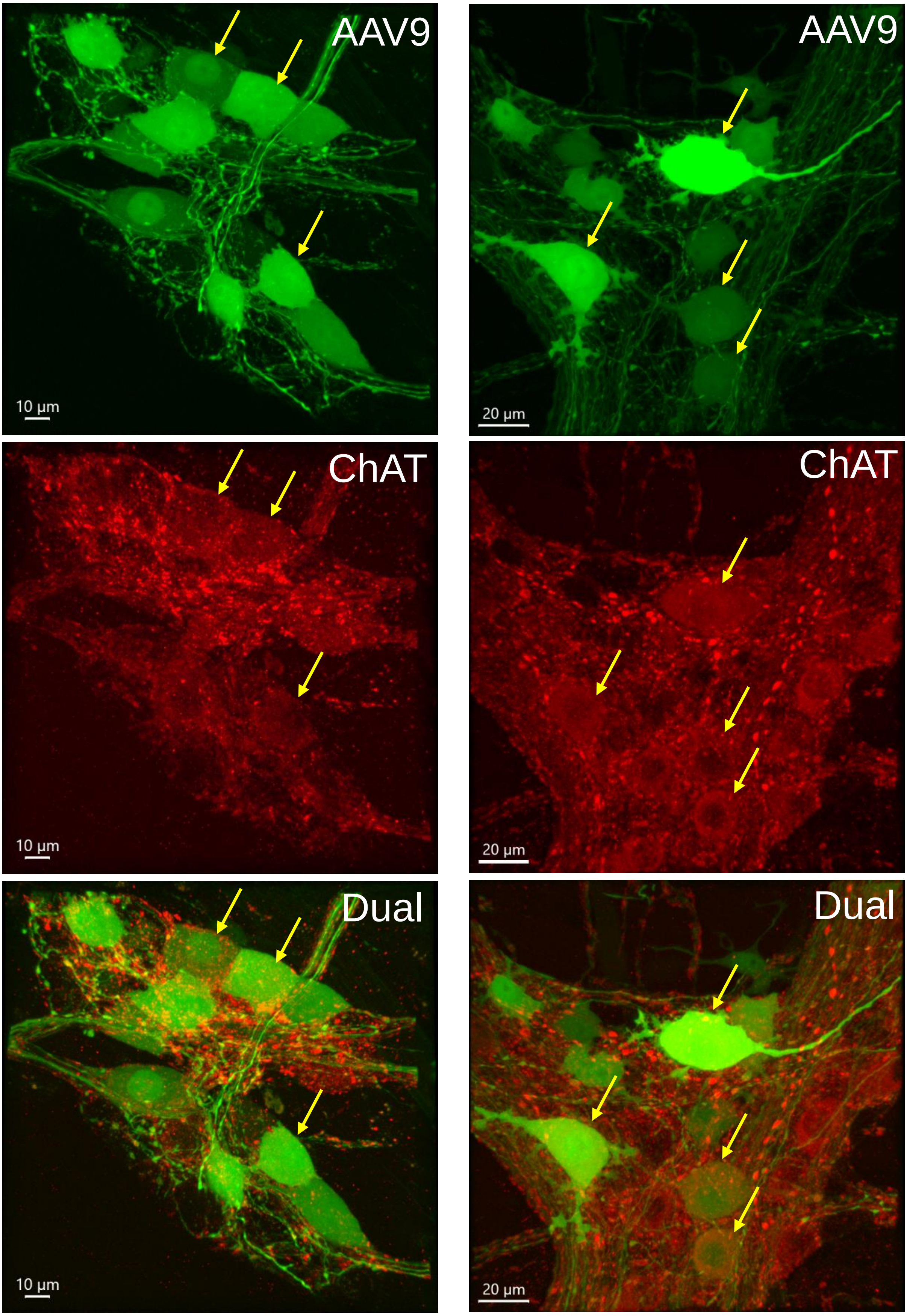

Supplementary Figure 4. Higher magnification of AAV9 transduction combined with immunofluorescence of ChAT in the submucosal plexus (left panels) and the myenteric plexus (right panels) of the mouse proximal colon. The arrows mark some dual labeled neurons.

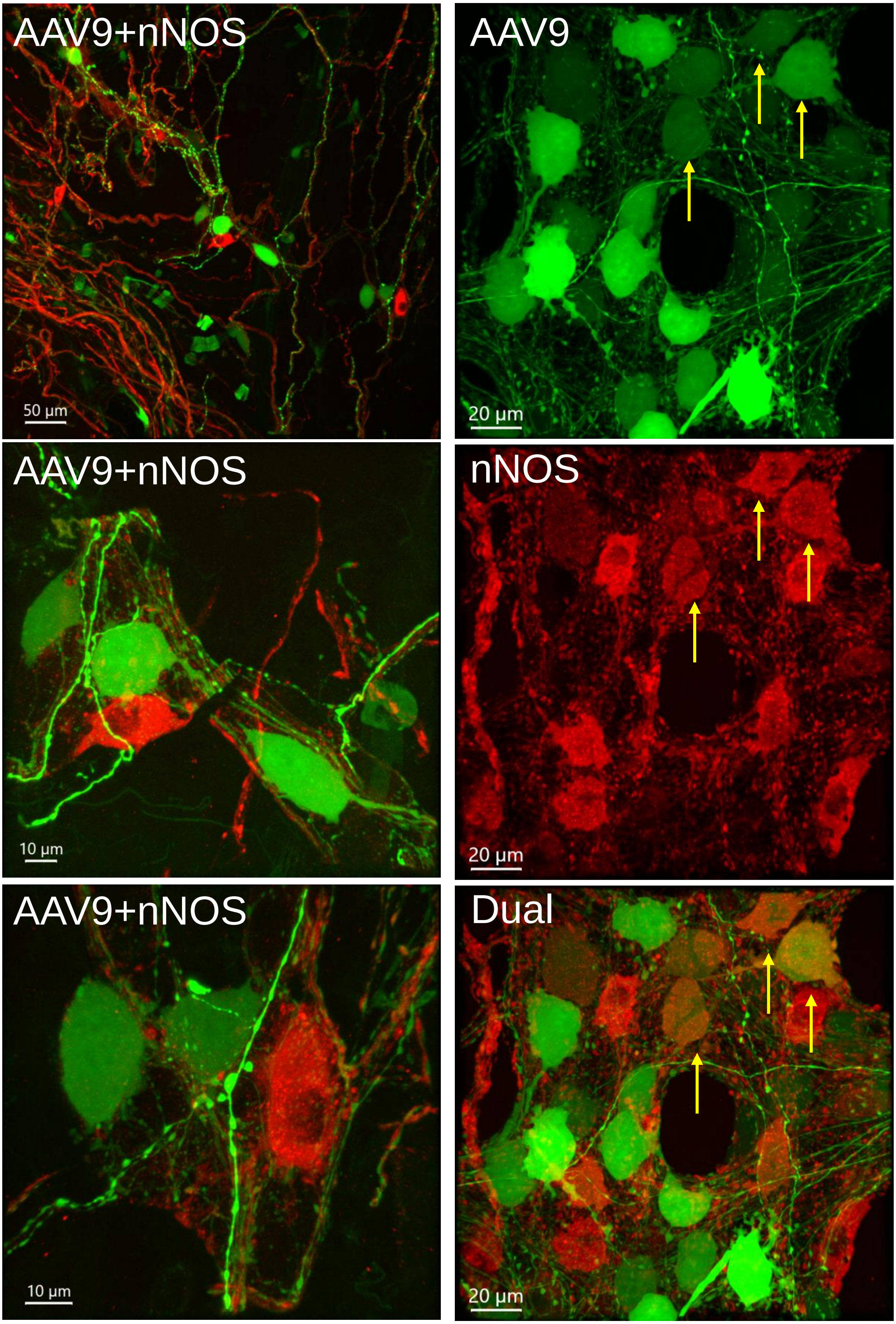

Supplementary Figure 5. Photomicrographs of AAV9 labeling combined with immunofluorescence of nNOS in the submucosal plexus (left panels) and the myenteric plexus (right panels) of the mouse proximal colon. The dual labeled neurons appeared in the myenteric plexus, and rarely found in the submucosal plexus.

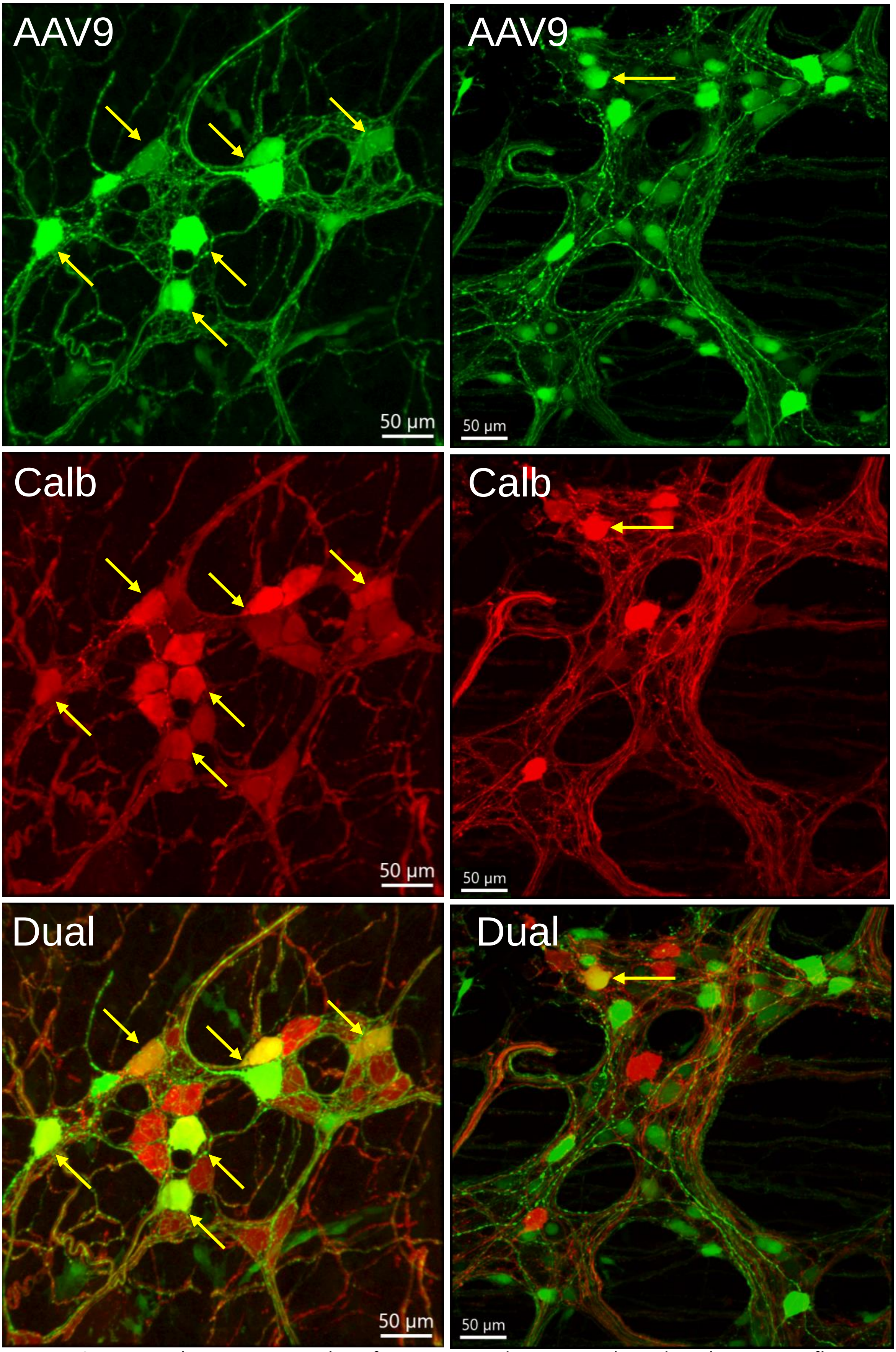

Supplementary Figure 6. Photomicrographs of AAV9 transdution combined with immunofluorescence of calbindin (Calb) in the submucosal (left panels) and myenteric plexus (right panels) of the mouse proximal colon. Many neurons in the submucosal plexus were calb-ir and so were the majority of AAV9-transduced neurons (arrows). In the myenteric plexus, much less calb-ir and double-labeled neurons (arrow).

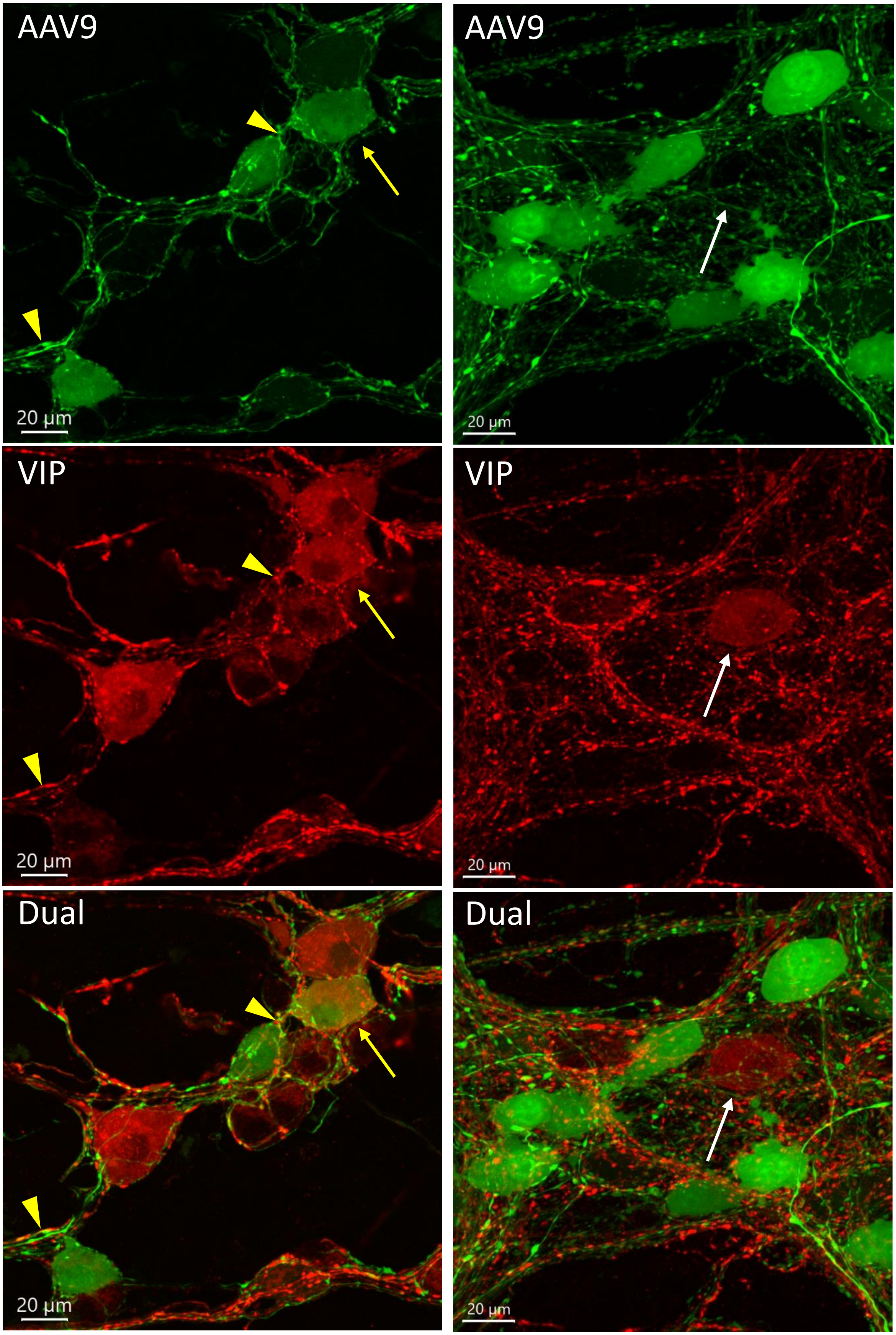

Supplementary Figure 7. AAV9 transduction combined with VIP immunofluorescence in the submucosal plexus (left panels) and myenteric plexus (right panels) of the mouse proximal colon. In the submucosal plexus, many neurons were VIP-ir, and some double labeled neurons (arrow) and nerve endings (arrowheads). In the myenteric plexus, abundant VIP-ir nerve fibers and endings, but few VIP-ir neurons.

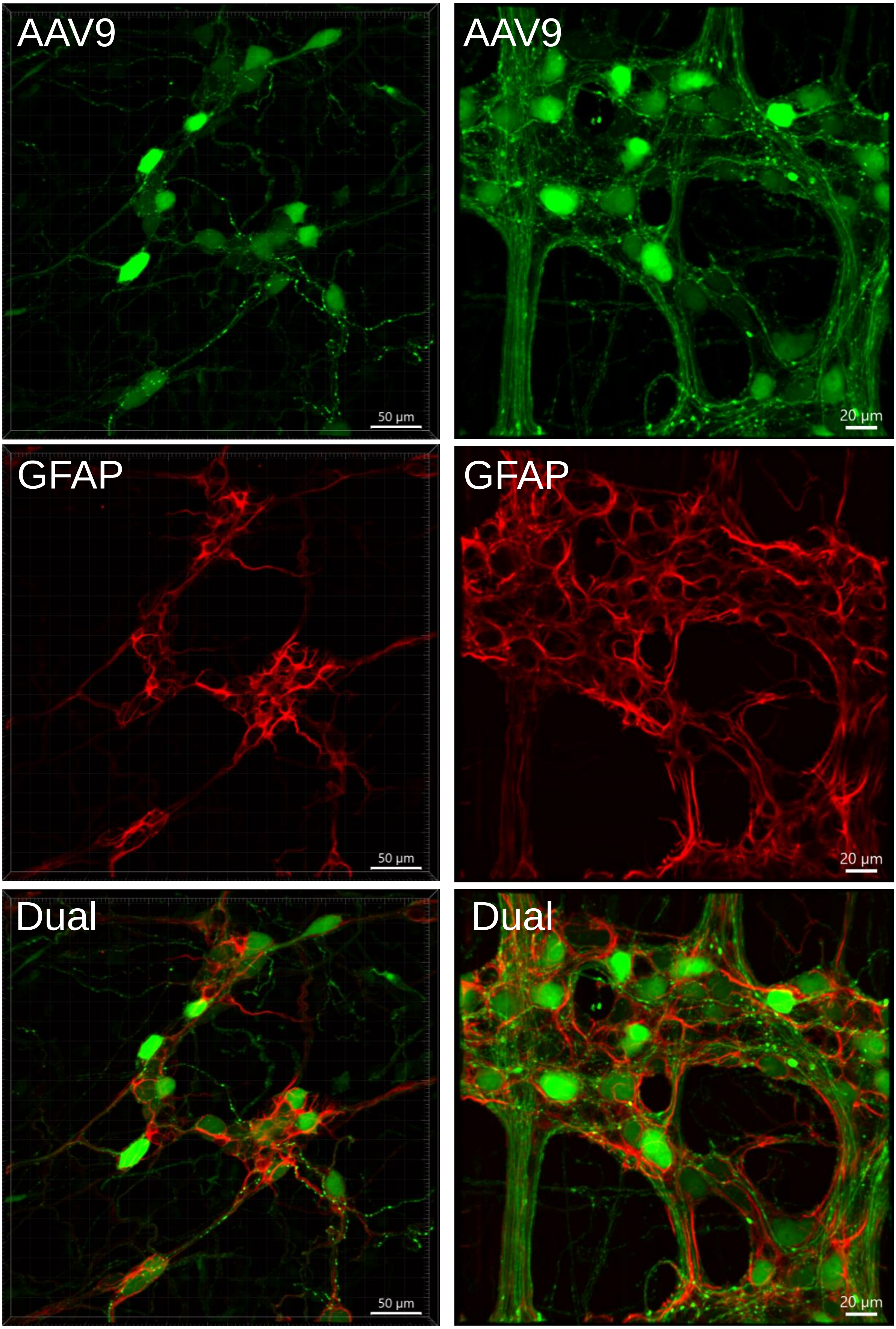

Supplementary Figure 8. AAV9-tranduced cells were not double-labeled by GFAP immunoreactivity in both submucosal (left panels) and myenteric plexus (right panels) of mouse proximal colon.

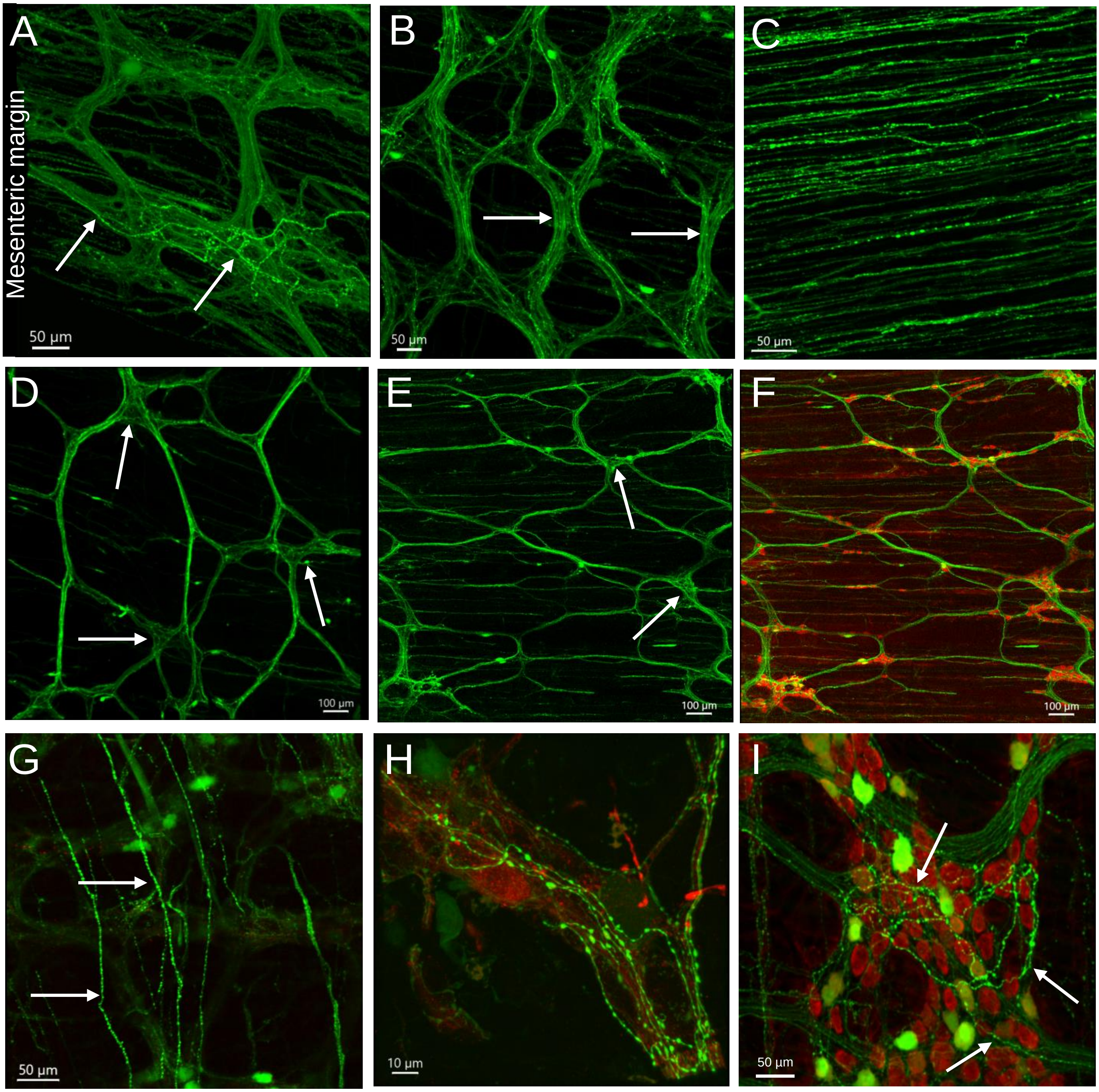

Supplementary Figure 9. Examples of AAV9-transduced nerve fibers in the mouse proximal colon. The longitudinal axis of all samples in the photomicrographs is placed along the vertical direction. **A.** One nerve fiber from the mesenteric margin terminated in a ganglion nearby (arrows); **B.** The longitudinal interganglionic strands as indicated by arrows; **C.** Dense circumferential nerve fibers in the circular muscular layer. **D.** Never fibers and myenteric ganglia in the mesenteric margin. **E.** Nerve fibers in the anti-mesenteric margin. Arrows in **D** and **E** indicate small ganglia in both margins. **F.** AAV9 labeling combined with HuC/D immunofluorescence showing the scattered neurons in the anti-mesenteric margin. **G.** AAV9-transduced longitudinal nerves in the longitudinal muscular layer. **H.** Nerve endings with big varicosities inside one myenteric ganglion with nNOS-ir. **I.** Nerve endings with big varicosities inside one myenteric ganglion immunostained with HuC/D to show the neurons.

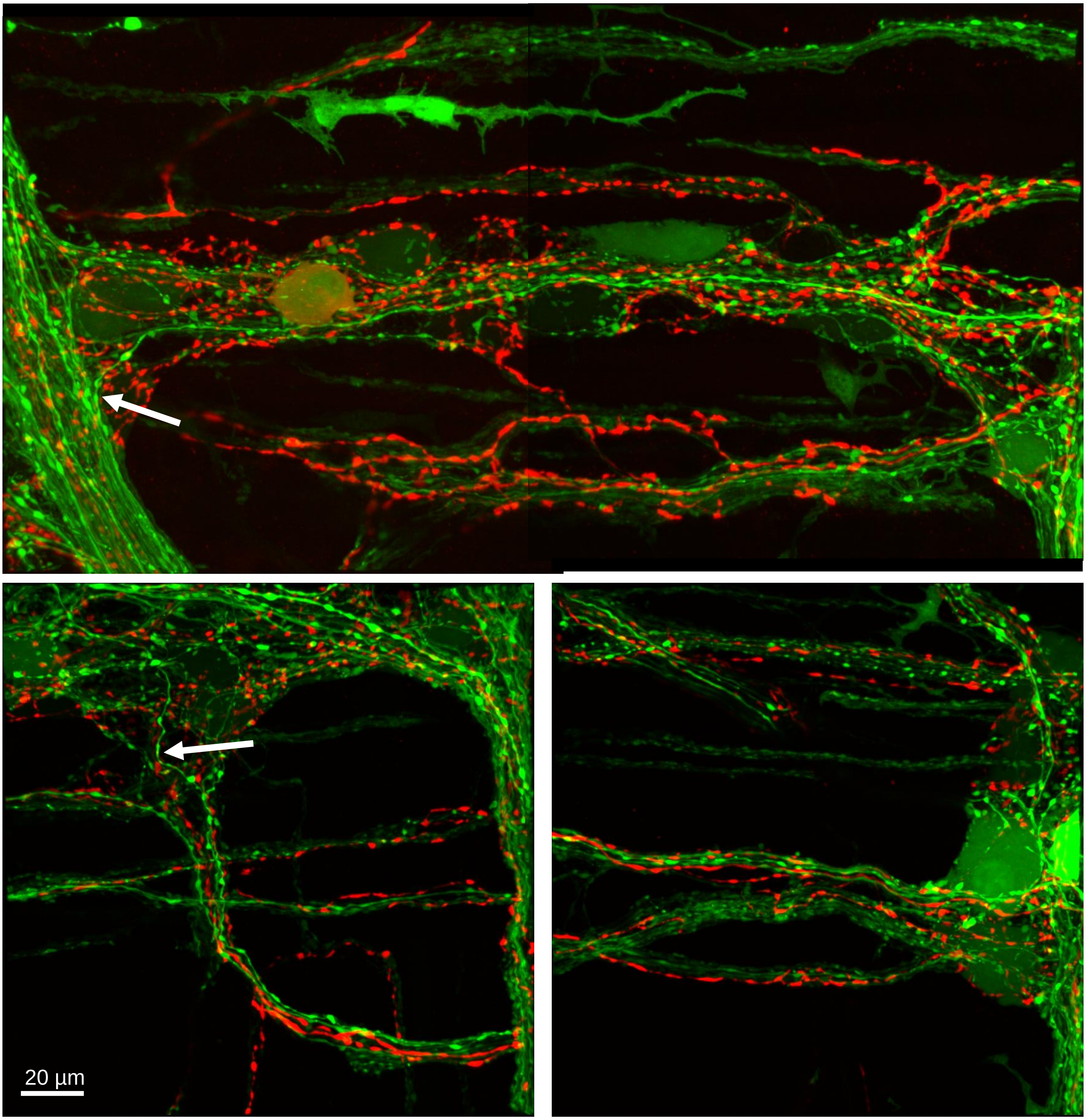

Supplementary Figure 10. Small circumferential interganglionic strands mixed with nerve fibers labeled by AAV9 transduction (green) and TH immunofluorescence (red) in the mouse proximal colon. Many small nerve bundles run circumferentially, connecting myenteric ganglia or joining the longitudinal inter-ganglionic strands (arrows).

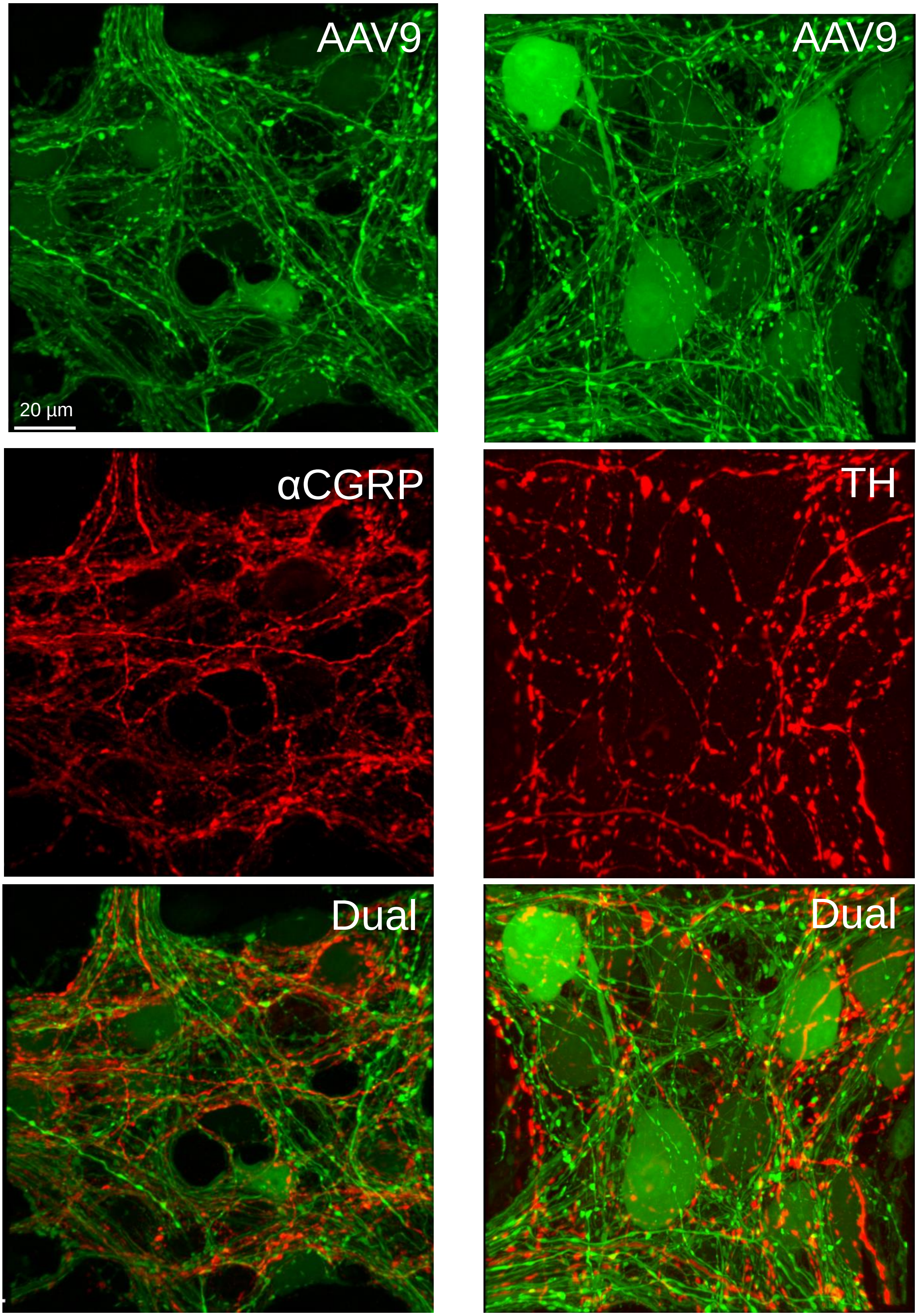

Supplementary Figure 11. High magnification of AAV9 transduction in the myenteric plexus whole mount preparations of the mouse proximal colon combined with immunofluorescence of calcitonin gene related peptide-alpha (αCGRP) and tyrosine hydroxylase (TH). Almost no co-localization of AAV9 and αCGRP, or AAV9 and TH are found. Scale: 20 µm same for all images.

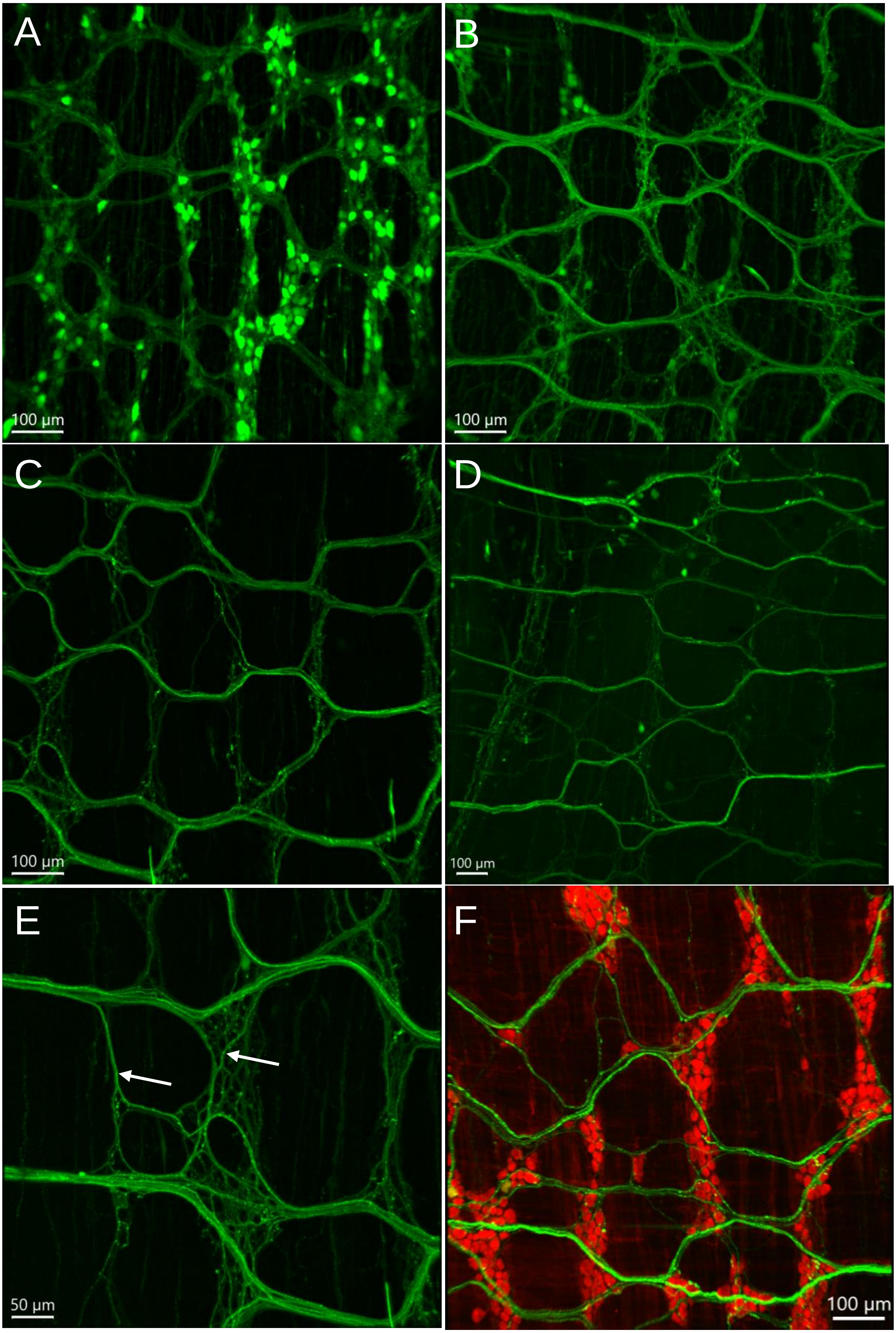

Supplementary Figure 12. Differences of AAV9 transduction in the mouse transverse (tC) and distal colon (dC). **A-C.** Contrast changes of AAV labeling in the tC from oral to middle and aboral parts; **D.** dC. The longitudinal interganglionic strands were prominent in the tC (**B, C**) than dC (**D**). **E.** a magnified image of the myenteric plexus in the caudal tC showing AAV9-transduced nerve fibers and terminals, and longitudinal fibers crossing (arrows). **F.** a sample of dC combined with NeuN immuno-staining, almost no AAV9 labeled neurons.

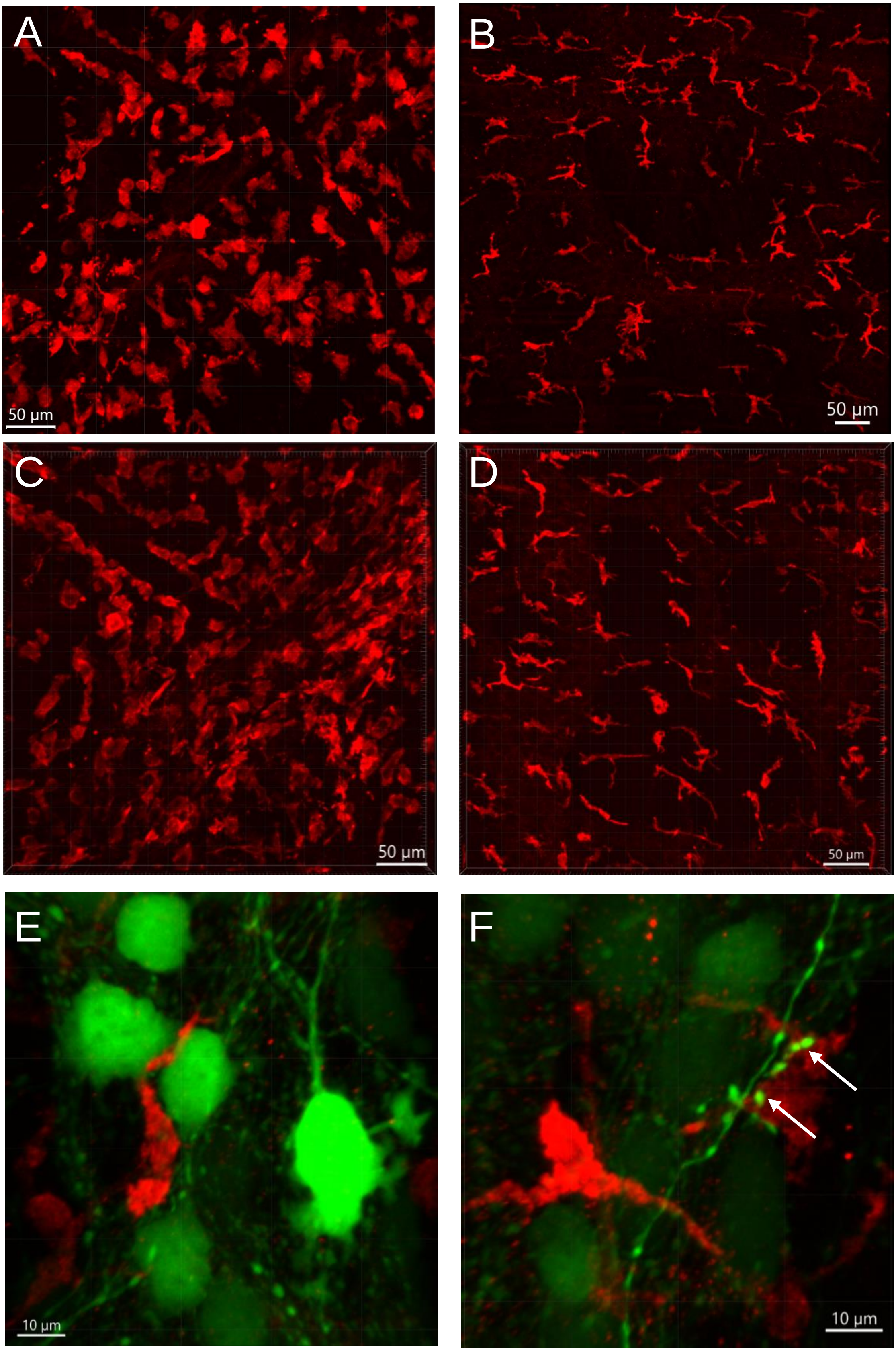

Supplementary Figure 13. Iba1-immunofluorescent macrophages in the submucosal (A, C) and between muscle layers (B, D) in the mouse proximal colon, similar morphology and distributions in naïve (A, B) or AAV9 iv injected mice (C-F). The morphologies are different in the submucosal and muscle layers. Iba1 cells are found closely adjacent AAV9-transduced neurons (E) and fibers (F). Arrows in F indicate an Iba1 cell embedded varicosities with AAV9.

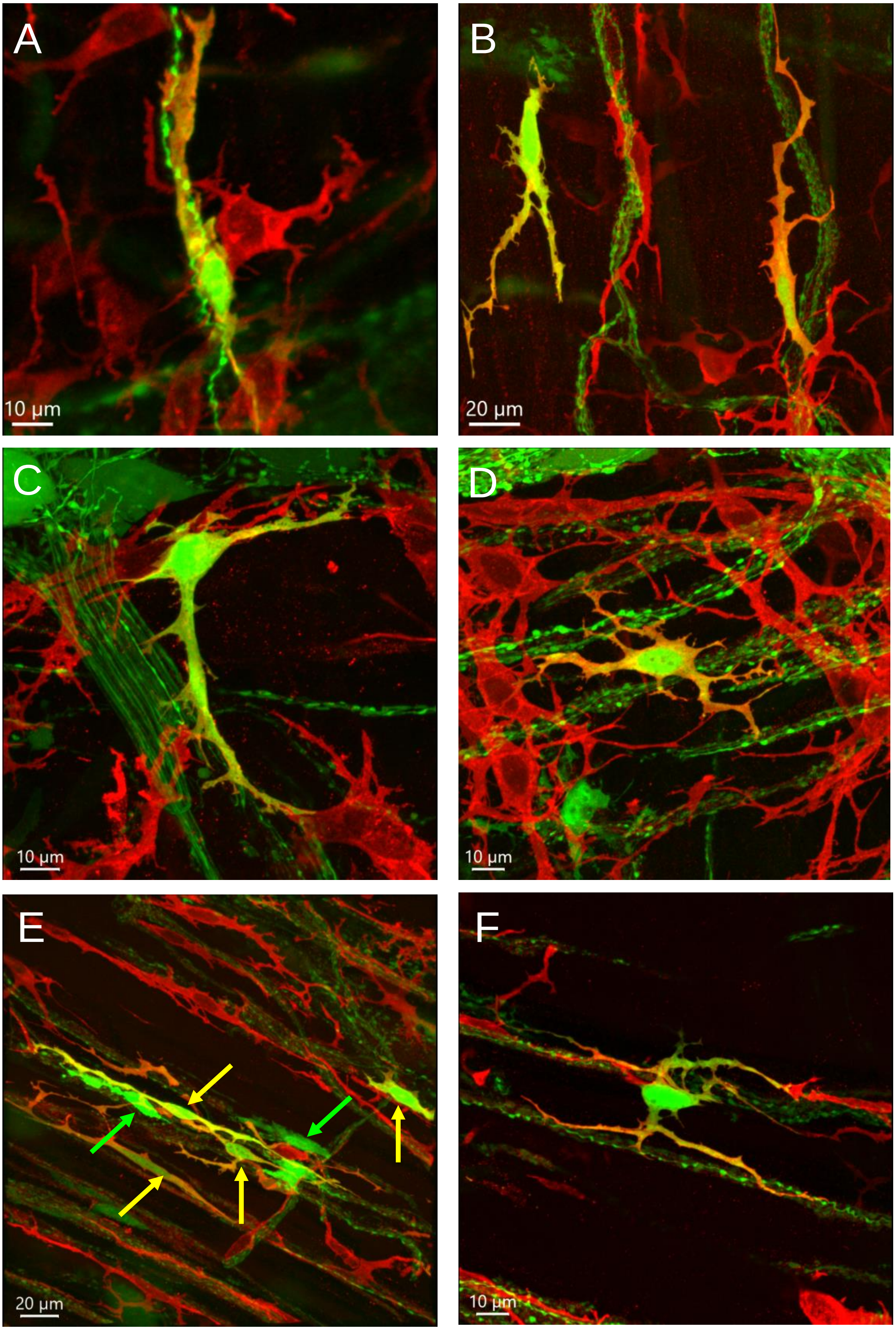

Supplementary Figure 14. Photomicrographs of c-Kit-ir (red) cells and transduction by iv AAV9 (green). Dual labeling was observed in c-Kit-ir cells (yellow). The c-Kit/AAV9 cells are polymorphic, and found in the longitudinal muscle layer (**A, B**), close to the myenteric plexus (**C, D**) and in the circular muscle layer (**E, F**). A group of AAV9-transduced cells were occasionally found with (yellow arrows) or without (green arrows) c-Kit immunoreactivity (**E**); one cell span its processes in two parallel nerve bundles (**F**).

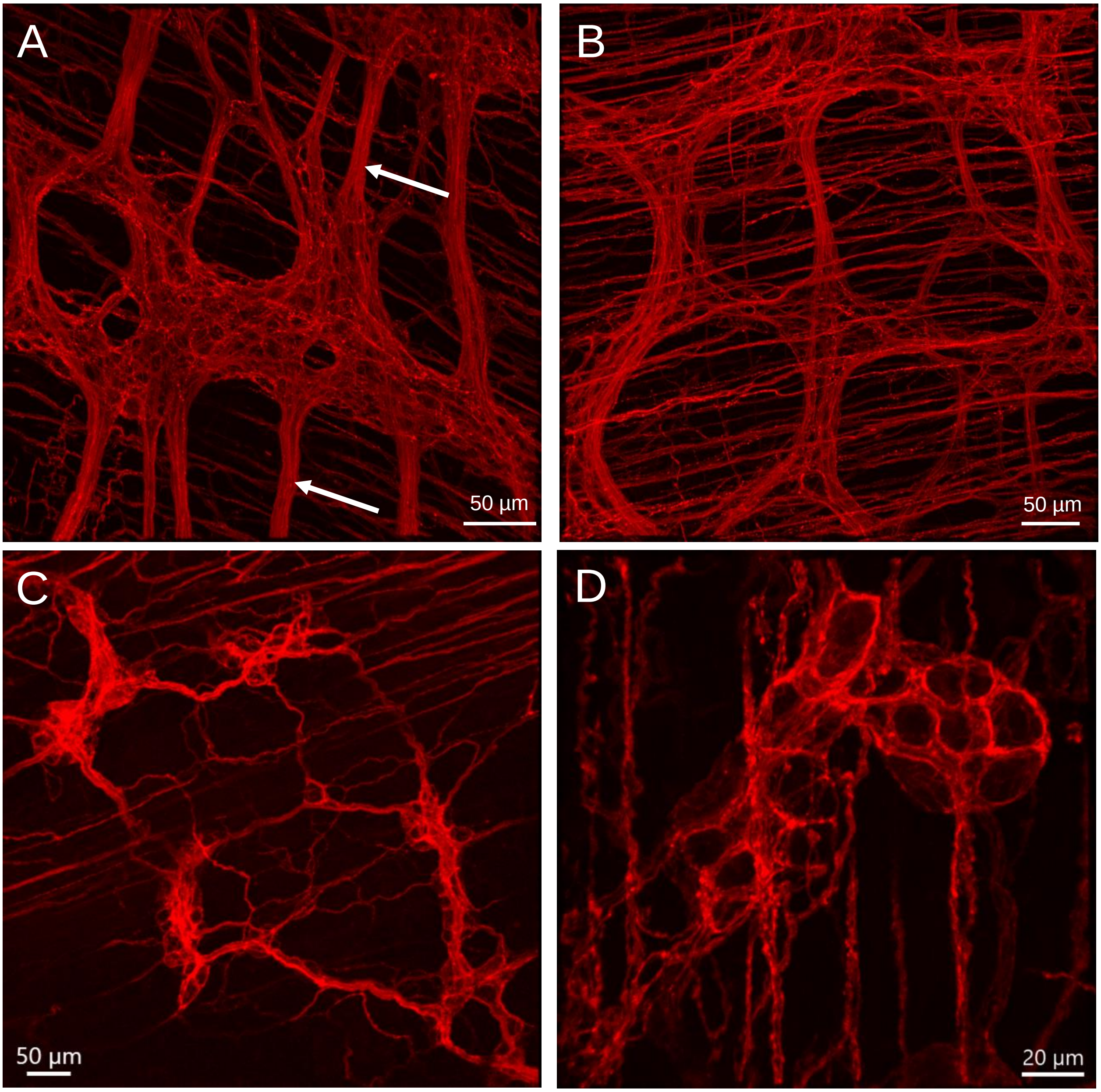

Supplementary Figure 15. PHP.S-tdTf transduced nerve fibers in the mouse proximal colon. **A.** thick longitudinal intergnaglionic strands in the myenteric plexus as indicated by arrows; **B.** dense circumferential nerve fibers in the circular muscle layer and nerve fibers in the myenteric plexus; **C** and **D**. PHP.S-tdTf-labeled nerve fibers in the submucosal plexus.

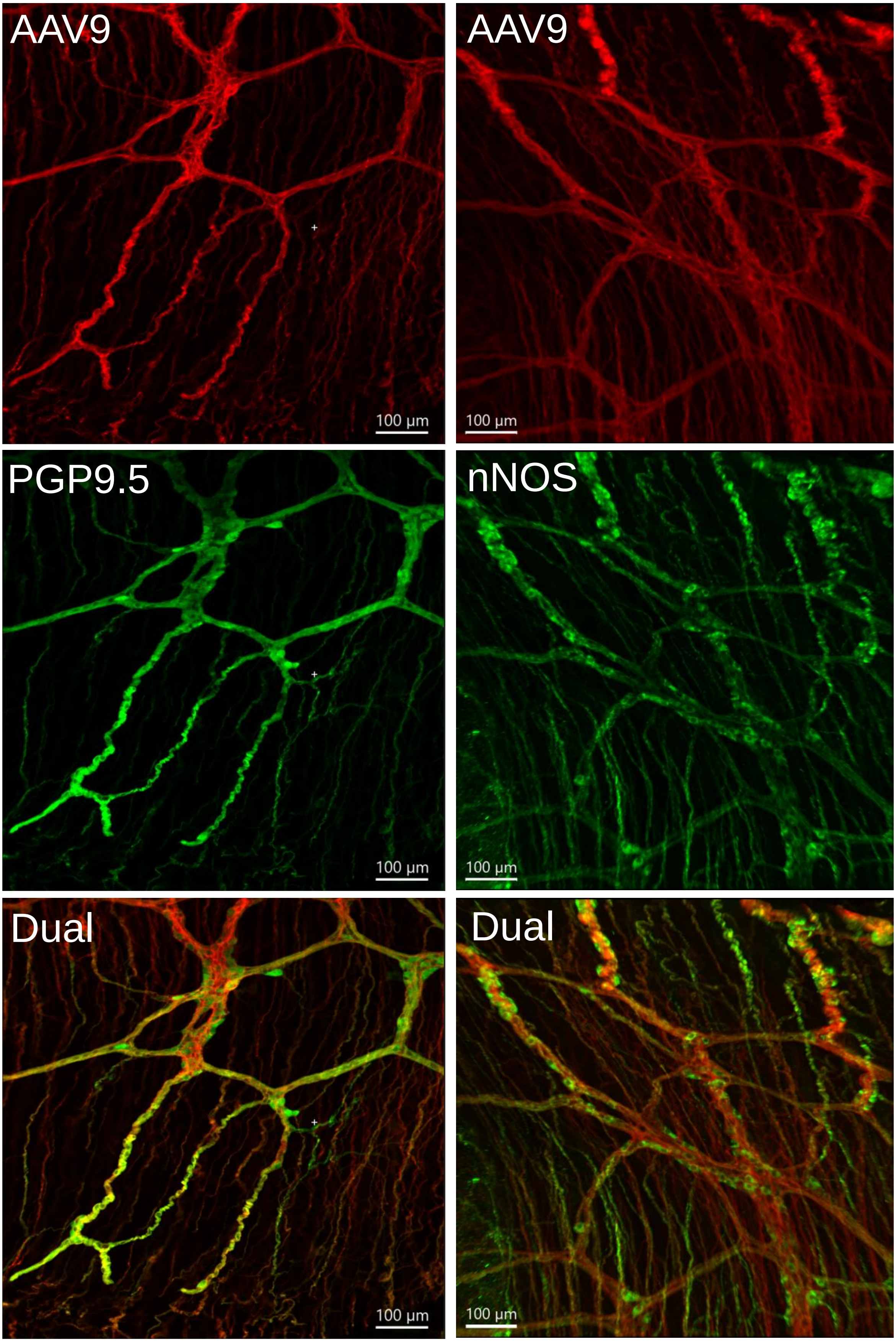

Supplementary Figure 16. Photomicrographs of PHP.S-tdTf transduced nerve fibers in the mesenteric margin of mouse proximal colon, double labeled with immunofluorescence of PGP9.5 (left) and nNOS (right).

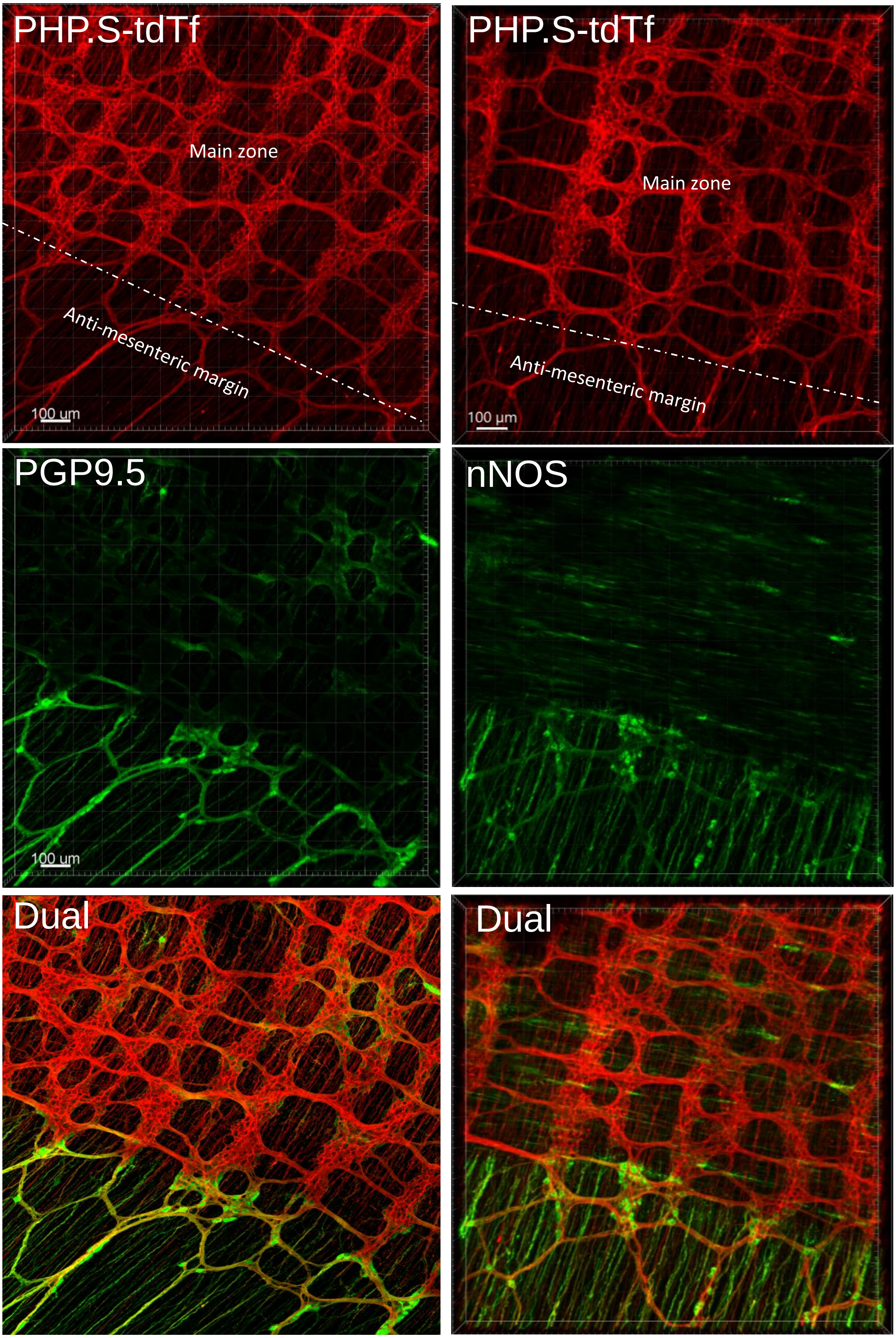

Supplementary Figure 17. PHP.S-tdTf transduction in the myenteric plexus of the mouse proximal colon combined with immunofluorescence of PGP9.5 and nNOS. The whole colonic wall was treated with 2% Triton X-100 PBS. PGP9.5-ir and nNOS-ir were poorly immunostained in the main zone, but good in the antimesenteric margin where contains scattered neurons. Scale bar 100 µm same for all images.

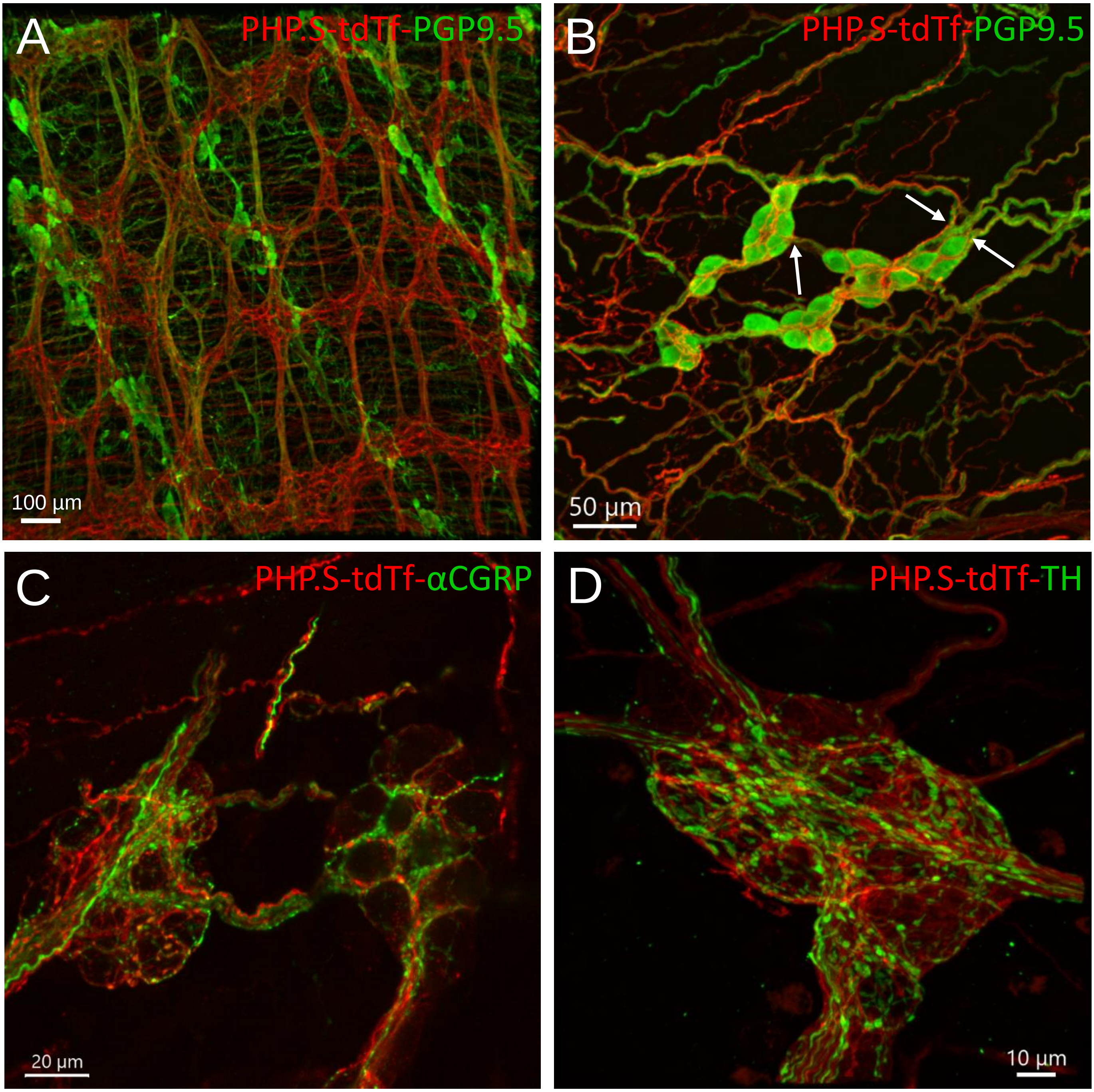

Supplementary Figure 18. Photomicrographs of double labeling of PHP.S-tdTf combined with immunofluorescence of PGP9.5, αCGRP or TH in the submucosal plexus of the mouse proximal colon. The mucosa were removed and the colon wall samples were pretreated with 2% TPBS. **A.** an image with double labeling of PHP.S-tdTf and PGP9.5 at low magnification shows the submucosal ganglia in chains located along the colonic mucosa folds, while the myenteric plexus with dense PHP.S-tdTf-transduced nerve fibers contained almost no immunolabeled neurons. **B.** PHP.S-tdTf-transduced nerves entered and connected the submucosal ganglia (arrows) and branched among the neurons with PGP9.5 immunoreactivity. **C** and **D**. Images at high magnifications show almost no co-existence of PHP.S-tdTf with αCGRP or TH in the nerve endings.

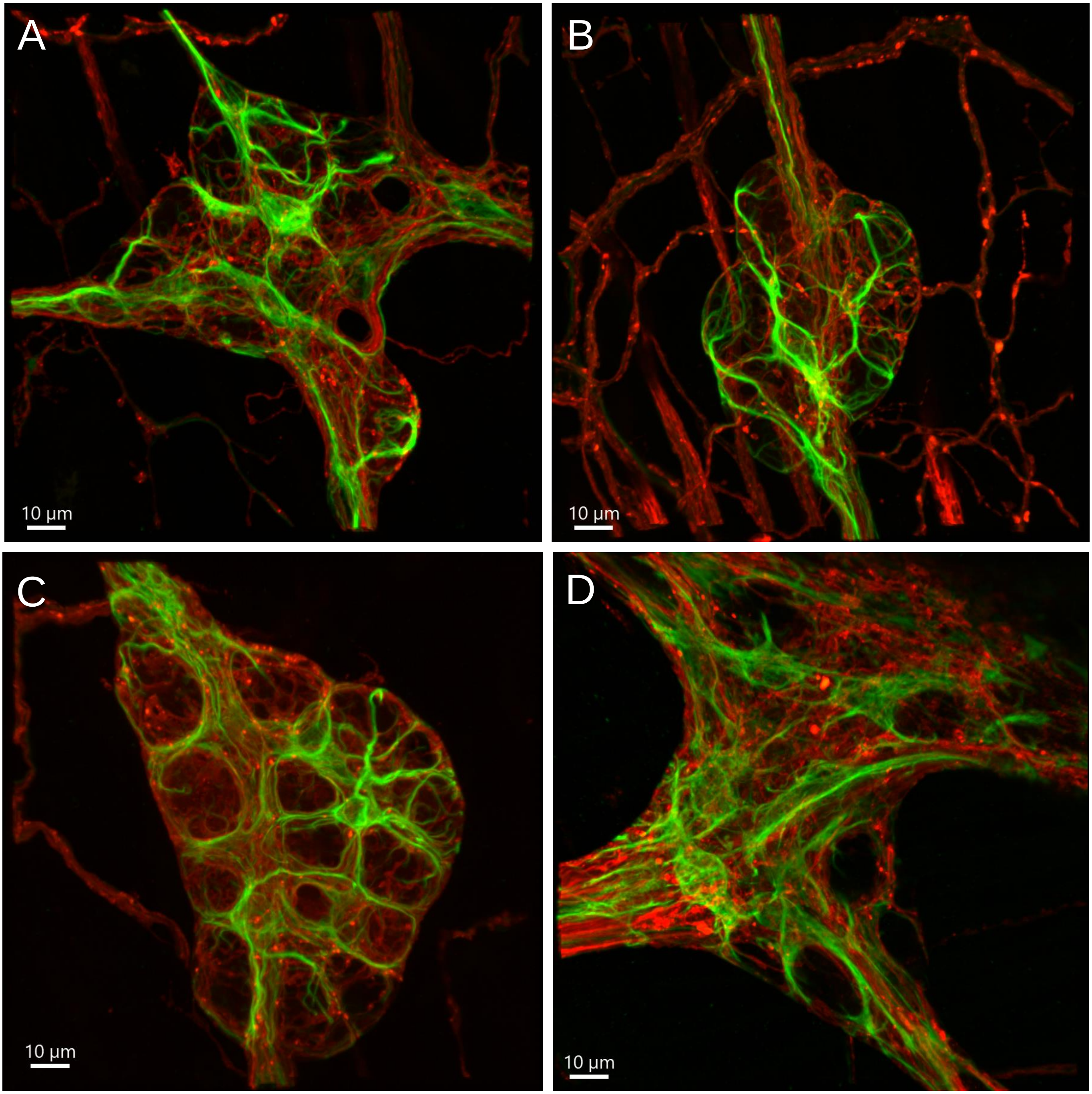

Supplementary Figure 19. Dual labeling by PHP.S-tdTf (red) and GFAP (green) shows locations of glial cells in relationship with nerve fibers in the submucosal (A-C) and myenteric (D) plexuses.

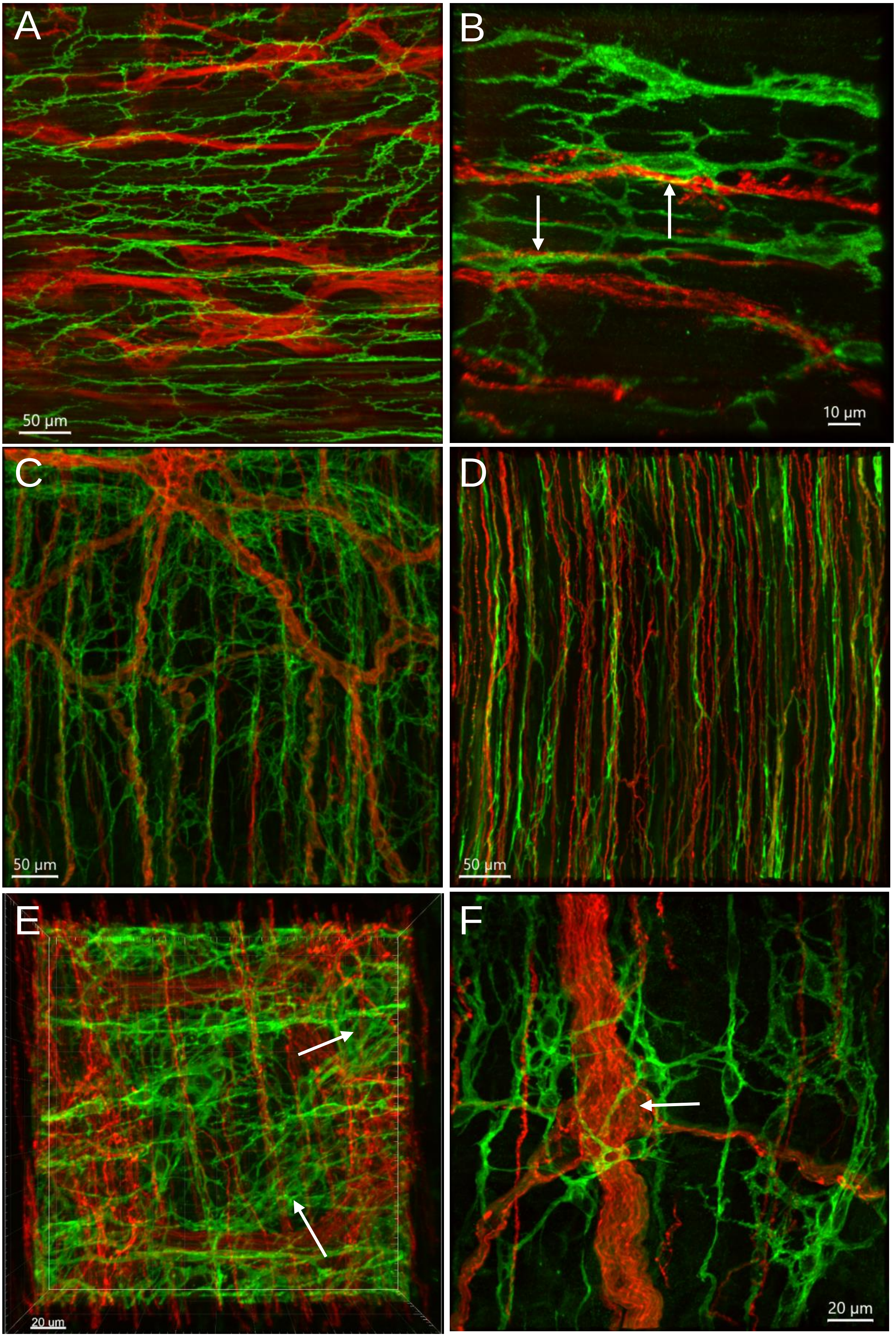

Supplementary Figure 20. Dual labeling of PHP.S-tdTf (red) and c-Kit-ir (green) in the mouse proximal colon. **A.** c-Kit-ir cells distributed in the longitudinal muscle layer; **B.** adjacent to the PHP-tdTf-transduced longitudinal nerves; **C.** in the edge of antimesenteric margin; **D.** along the PHP.S-tdTf-labeled circumferential nerves; **E.** in the spaces in the myenteric plexus (arrows); and **F.** nested around a small myenteric ganglion (arrow) near the mesenteric margin.

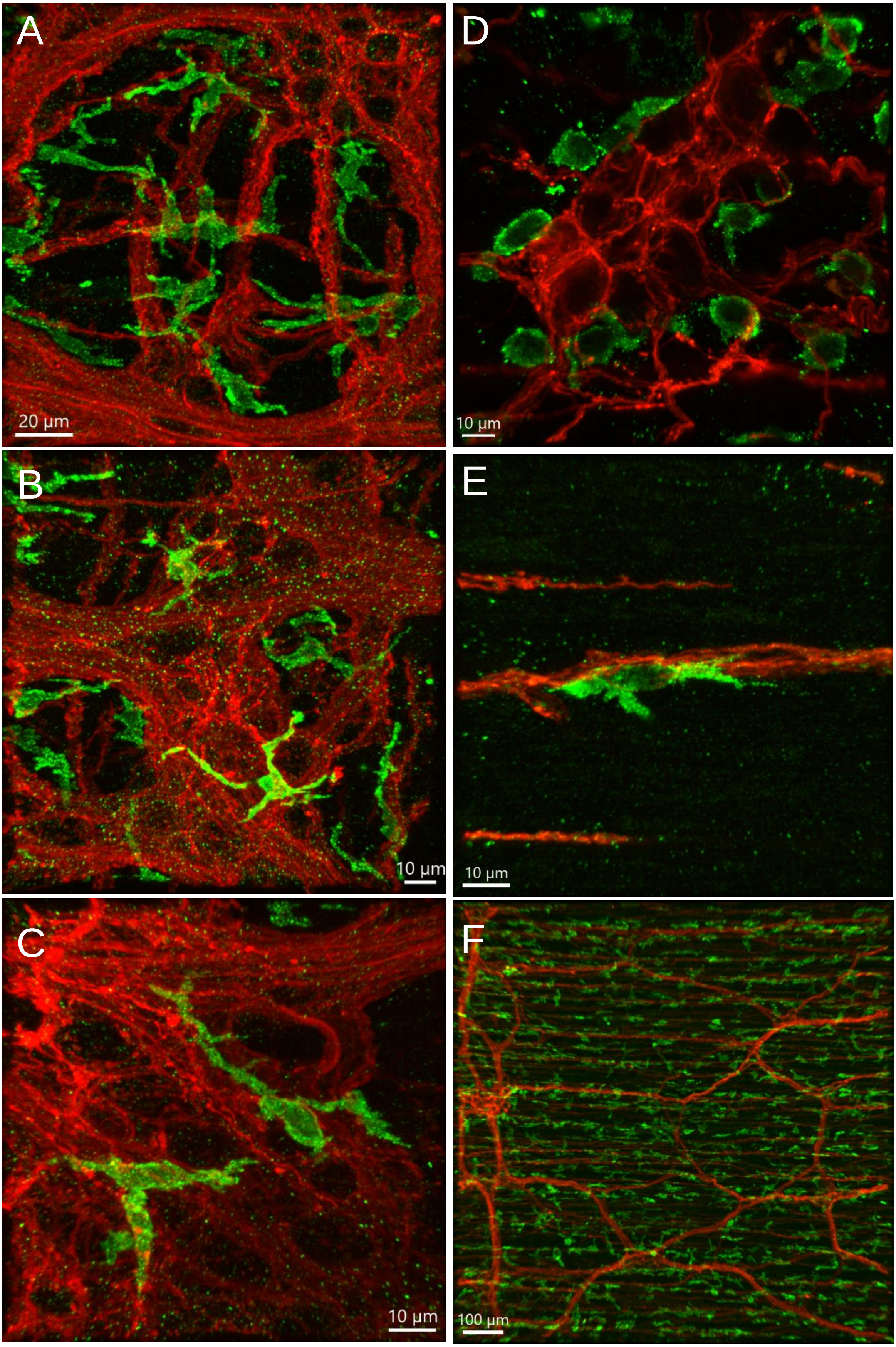

Supplementary Figure 21. Dual labeling of PHP.S-tdTf (red) and Iba1 (green) show relationship of macrophages to nerve fibers in the mouse proximal colon. Iba1-ir macrophages near (**A**) or in the myenteric ganglia (**B, C**). Macrophages close or in a submucosal ganglion (**D**). An Iba1-ir cell contacted circumferential nerves (**E**). Iba1-ir macrophages located in the anti-mesenteric margin (**F**).

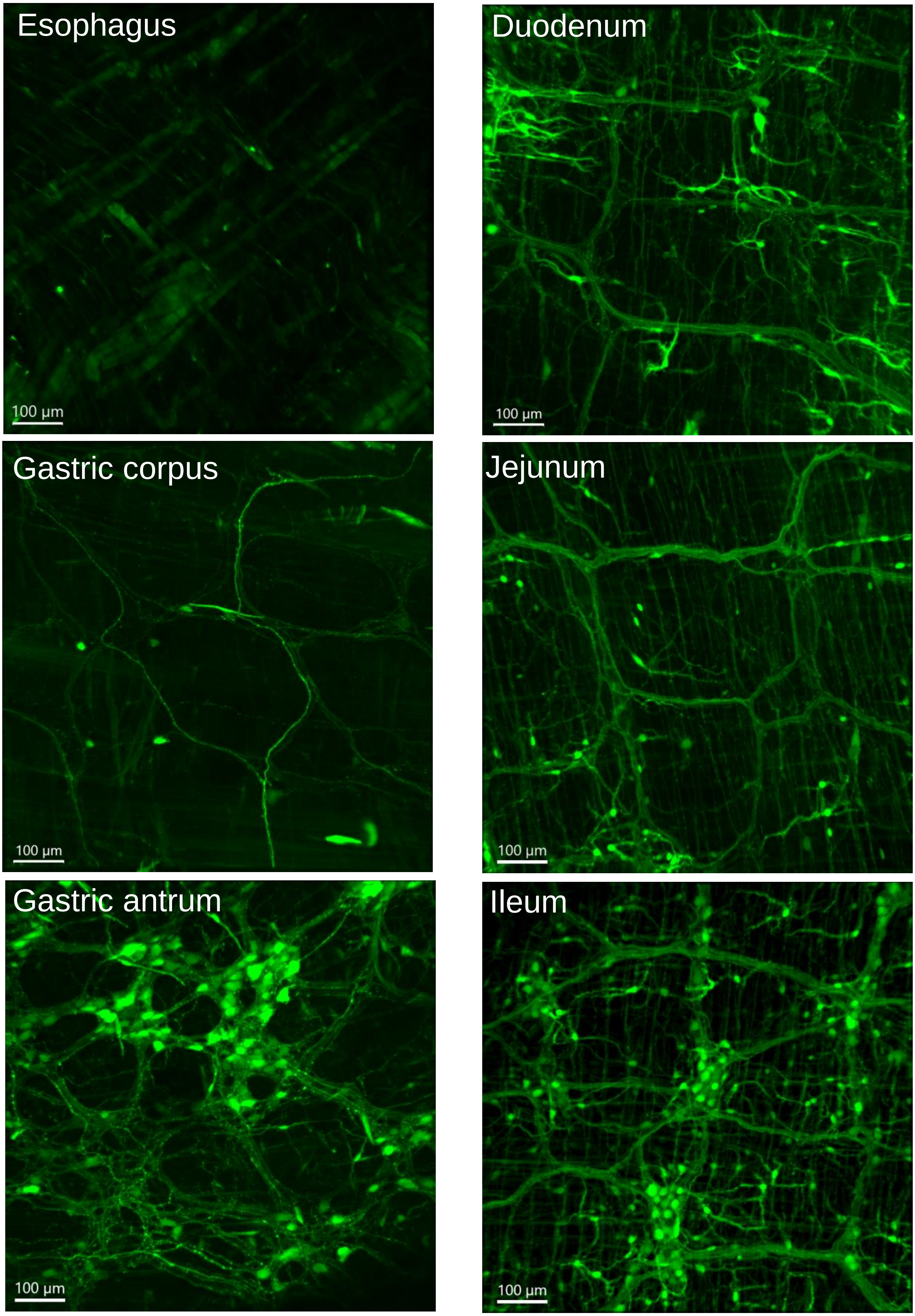

Supplementary Figure 22. Representative photomicrographs of AAV9 transduction in the mouse gastrointestinal tract from the esophagus to ileum. AAV9-CAG-GFP was retro-orbitally injected 1x10^{12} GC/mouse 3 weeks before. The preparations included layers of muscles and serosa by removal of the mucosa and scratching off part of the submucosa. Myenteric neurons were transduced more in the gastric antrum and ileum.

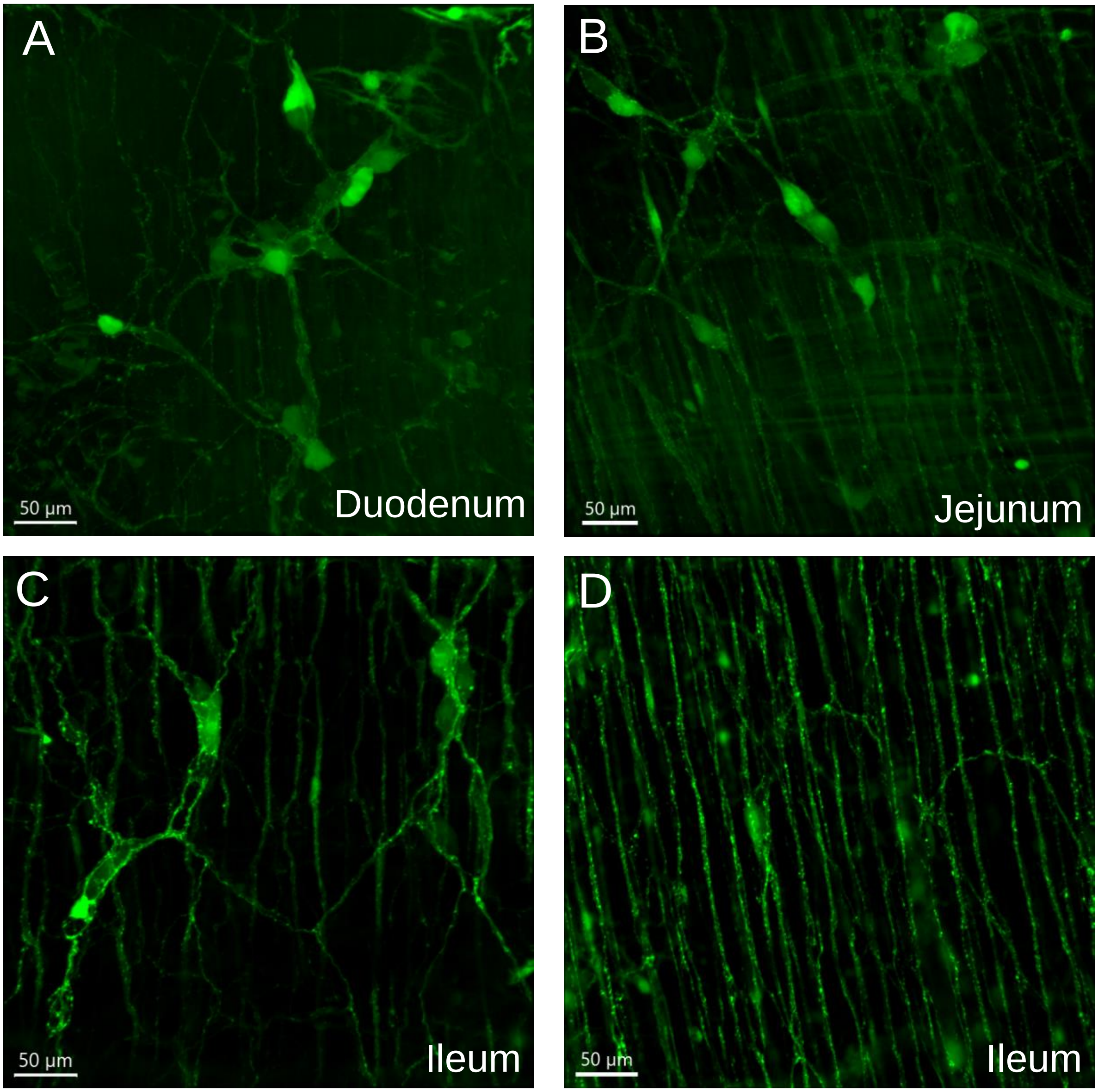

Supplementary Figure 23. Representative photomicrographs of AAV9 transduction in the submucosal plexus (A-C) and circular muscle layer (D) of the mouse small intestine. AAV9-CAG-GFP was retro-orbitally injected $1x10^{12}$ GC/mouse 3 weeks before.

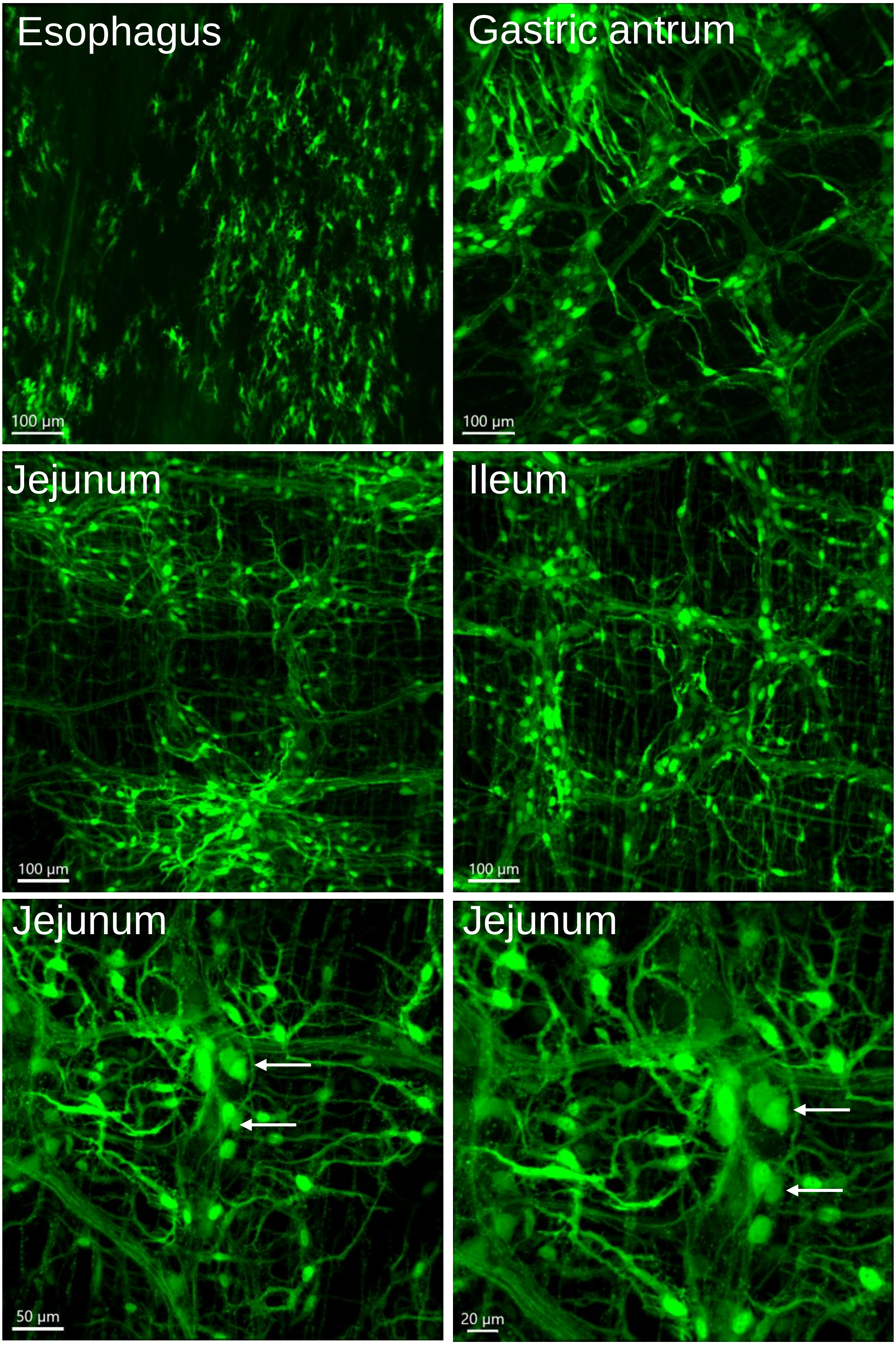

Supplementary Figure 24. Representative photomicrographs of AAV9 transduction in a male mouse gastrointestinal tract from the esophagus to ileum. AAV9-CAG-GFP was retro-orbitally injected $1x10^{12}$ GC/mouse 3 weeks before. The preparations included layers of submucosa, muscles and serosa by removal of mucosa. Many non-neuronal cells with processes mixed with neurons in the enteric ganglia (indicated by arrows in magnifications). Scales indicated in each image.

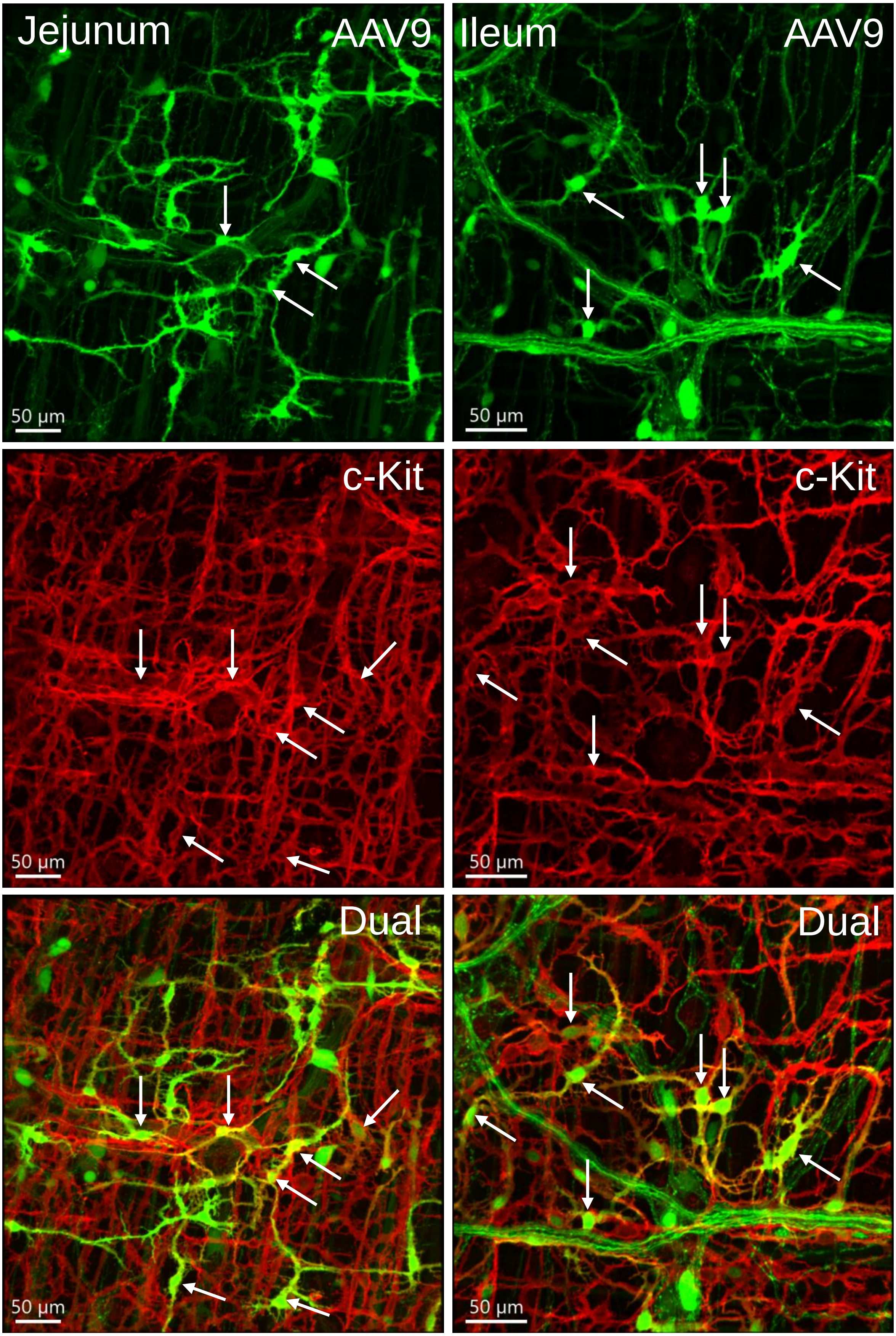

Supplementary Figure 25. Photomicrographs of c-Kit-ir (red) cells and transduction by iv AAV9 (green) in the mouse jejunum and ileum. Dual labeling often observed in c-Kit-ir cells (arrows)

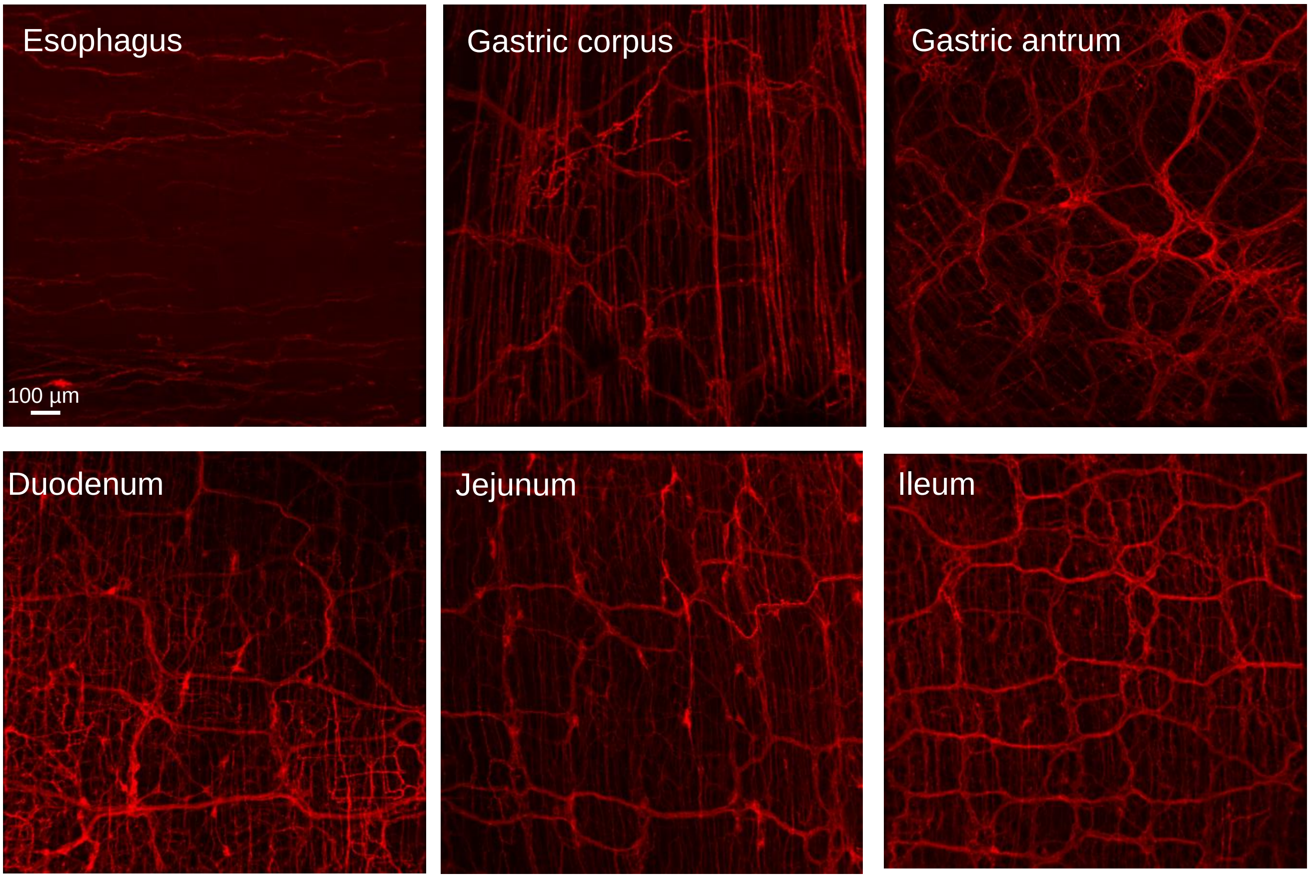

Supplementary Figure 26. Photomicrographs of the transduction by AAV-PHP.S-hSyn1-tdTomato farnesylated (PHP.S-tdTf) in a male mouse gastrointestinal tract (colon shown in Fig, 10). PHP.S-tdTf ($1x10^{12}$ GC/mouse) was retro-orbitally injected 3 weeks before. Scale: 100 µm same for all panels.

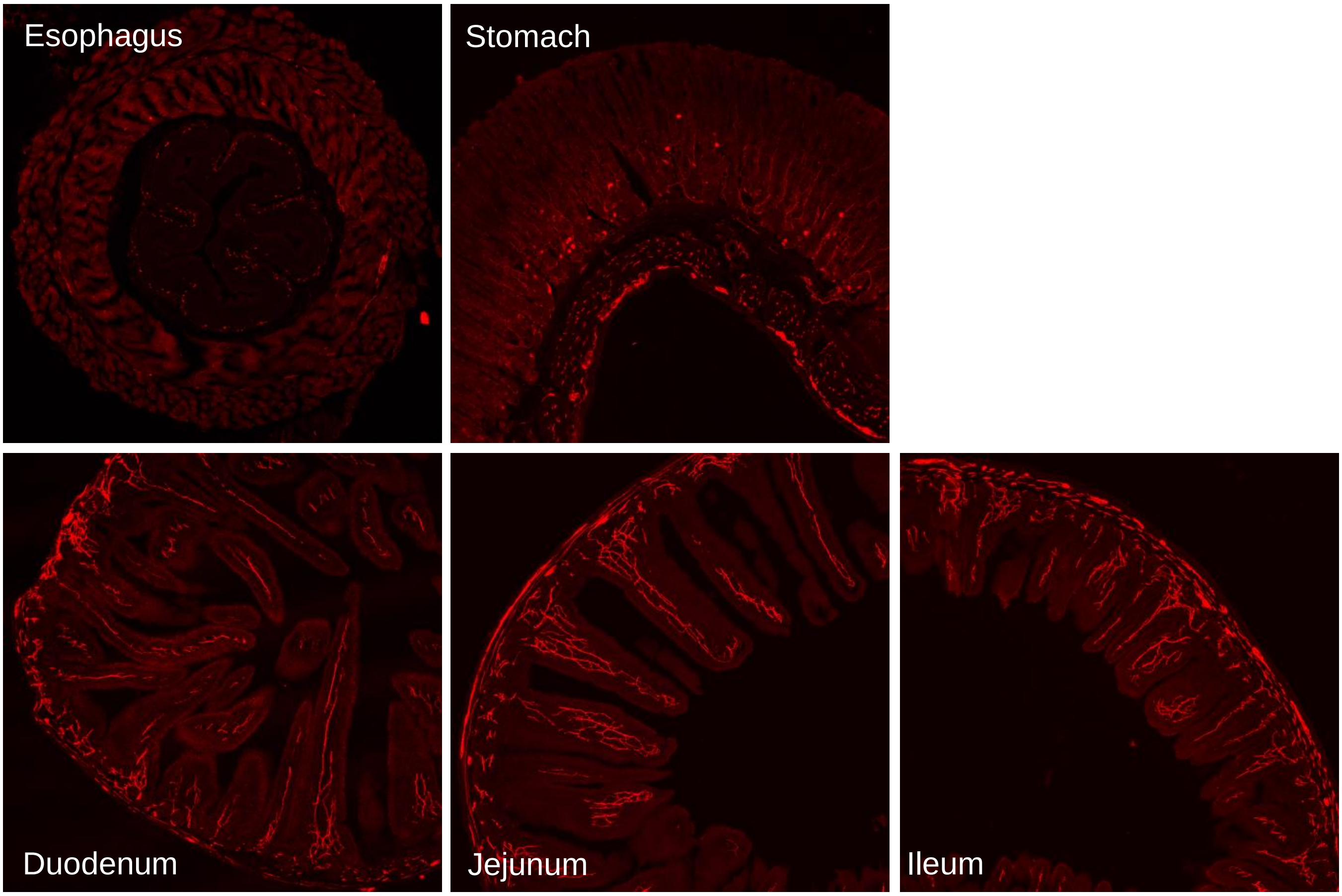

Supplementary Figure 27. Photomicrographs of the transduction by AAV-PHP.S-hSyn1-tdTomato farnesylated (PHP.S-tdTf) in a female mouse gastrointestinal tract (colon not included). PHP.S-tdTf ($1x10^{12}$ GC/mouse) was retro-orbitally injected 3 weeks before. Small intestine mucosa had more systemic AAV transduced nerves.

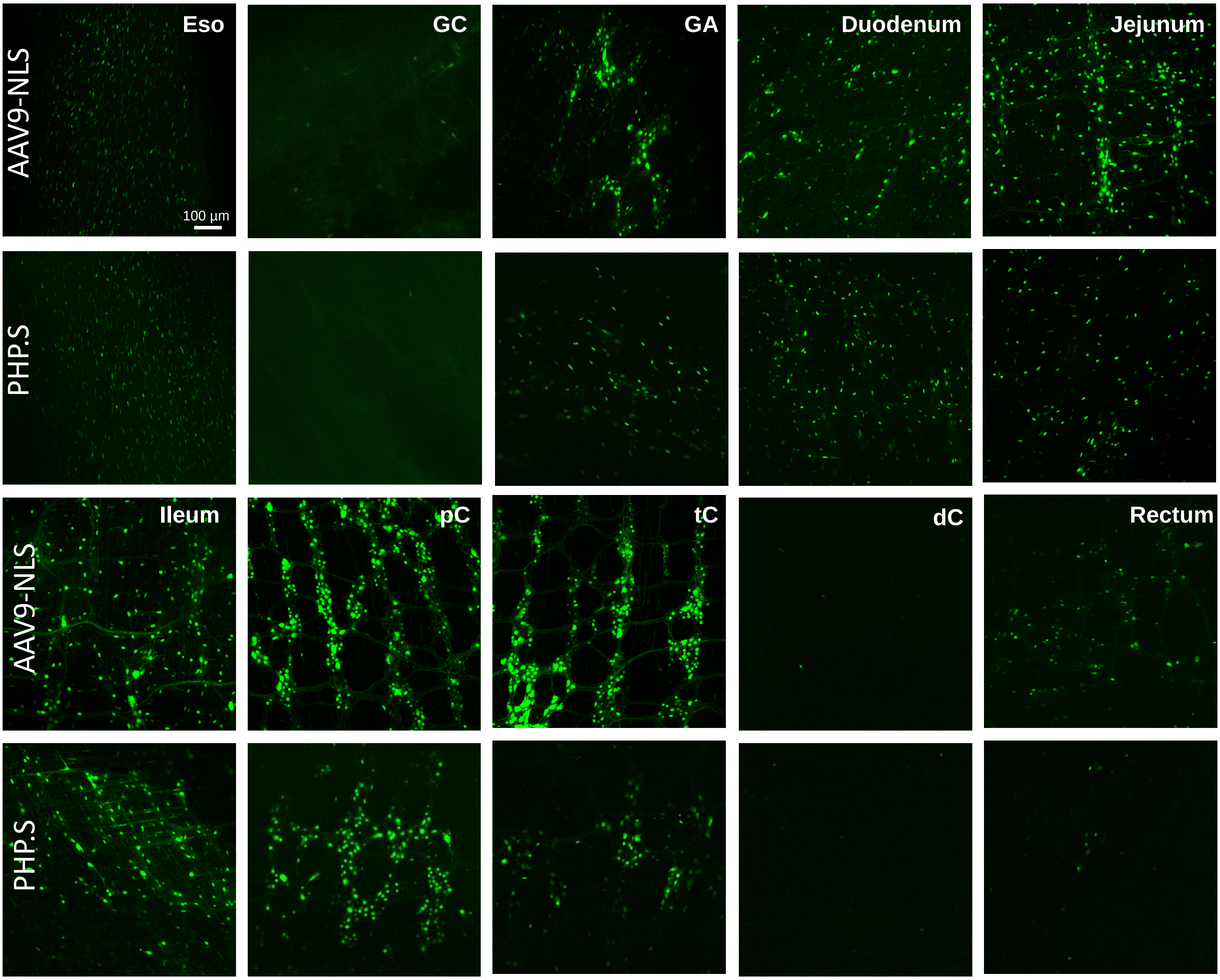

Supplementary Figure 28. Gastrointestinal mapping of transduction by AAV9-icap-CAG-NLS-eGFP (AAV9-NLS) and PHP.S-CAG-NLS-eGFP (PHPS) at3.3x10^{11} GC/mouse injected into the retro-orbital venous sinus of mice 3 week before. All panels same scale 100 µm as in the top-left image.

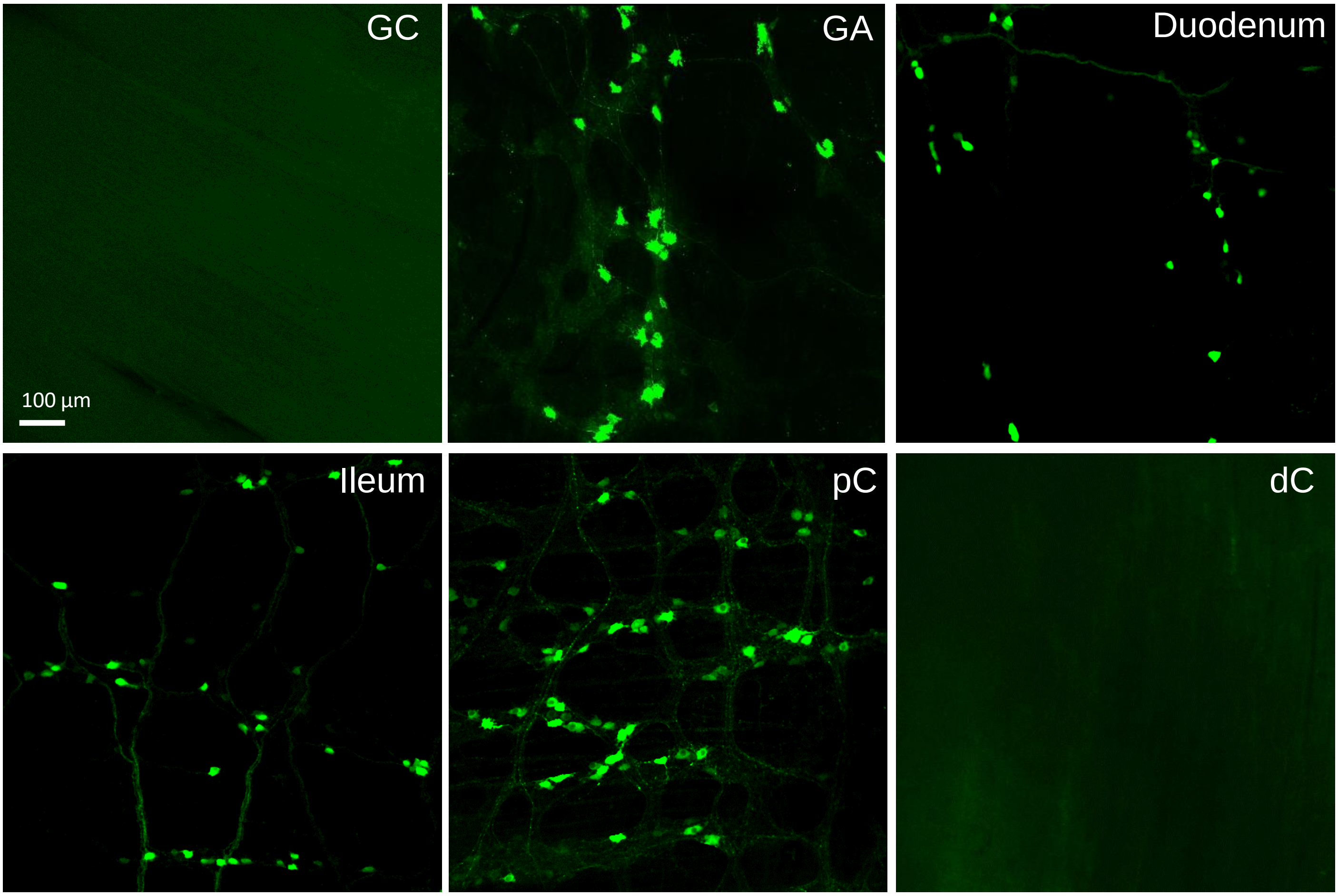

Supplementary Figure 29. GI mapping in ChAT-Cre mice. AAV-PHP.S-CAG-DIO-EYFP was retro-orbitally injected at injected 3.3×10^{11} GC/mouse 3 weeks before. All panels same scale 100 µm as in the top-left image.

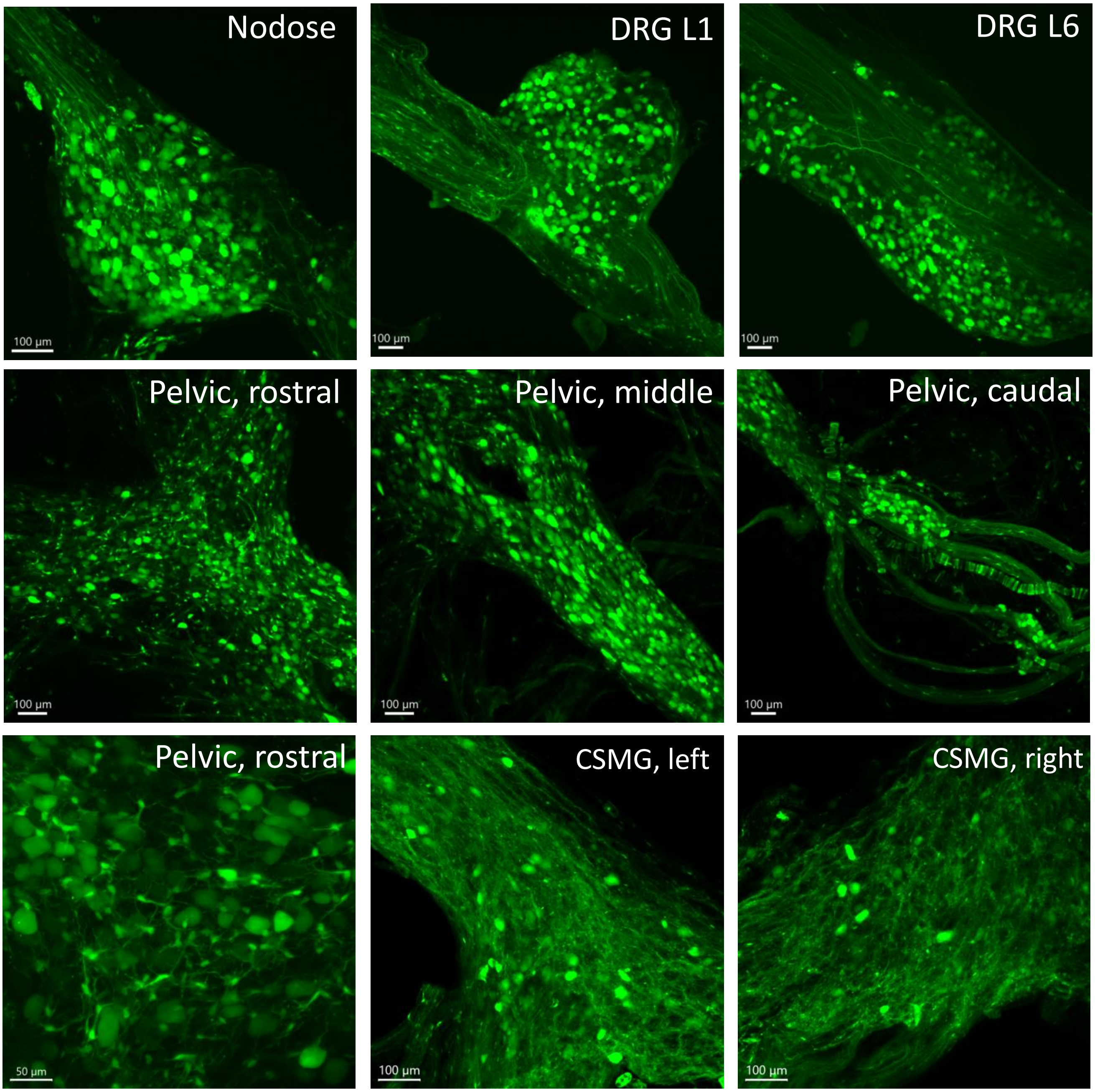

Supplementary Figure 30. AAV9-transduced neurons in the sensory and autonomic ganglia. AAV9-CAG-GFP was retro-orbitally injected 1x10[12] GC/mouse 3 weeks before. CSMG: celiac-superior mesenteric ganglion. DRG: dorsal root ganglion.

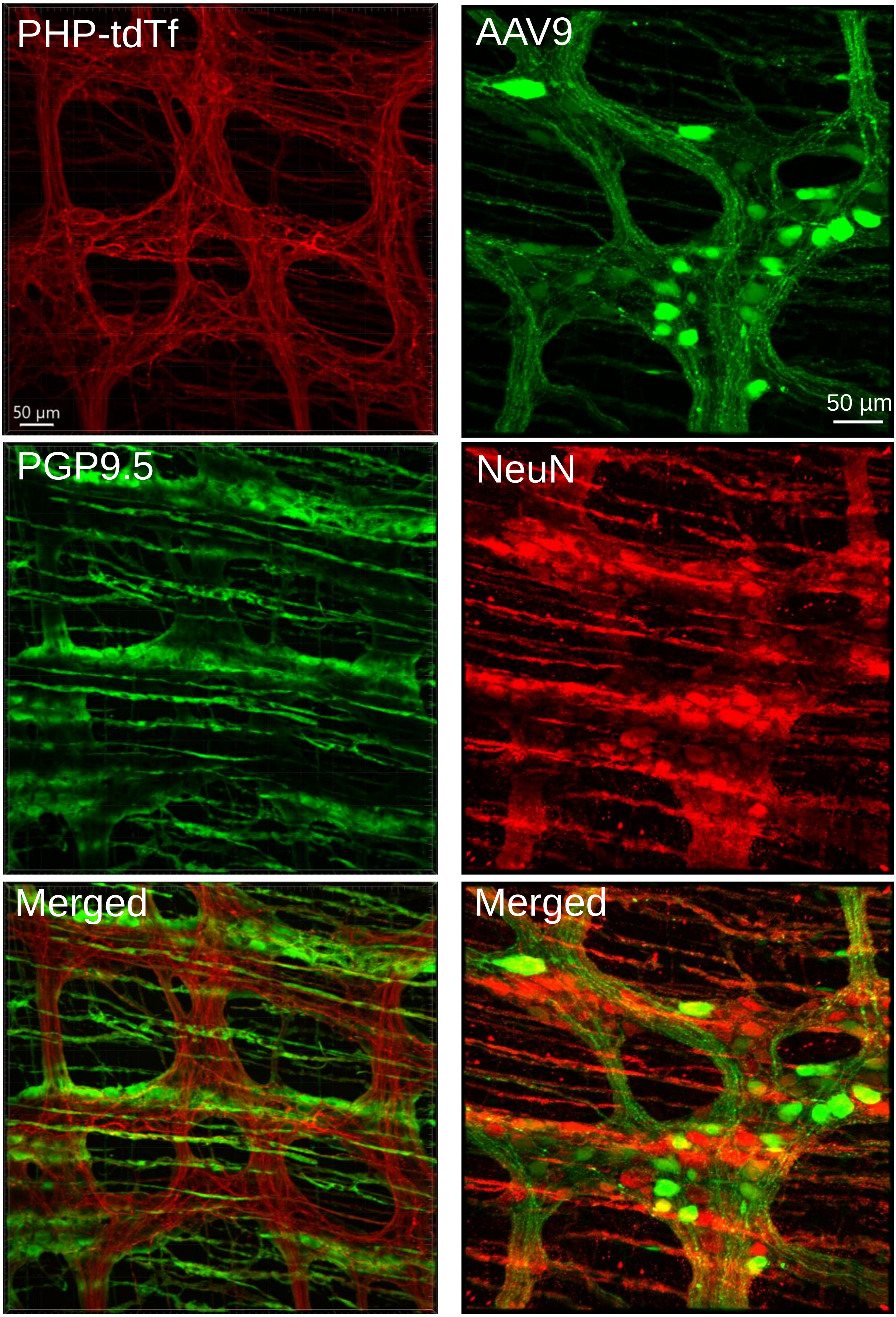

Supplementary Figure 31. Examples of smooth muscle strips remained on the myenteric plexus-longitudinal muscles in the whole mount preparations resulted strips without immunostaining of neuronal antibodies.

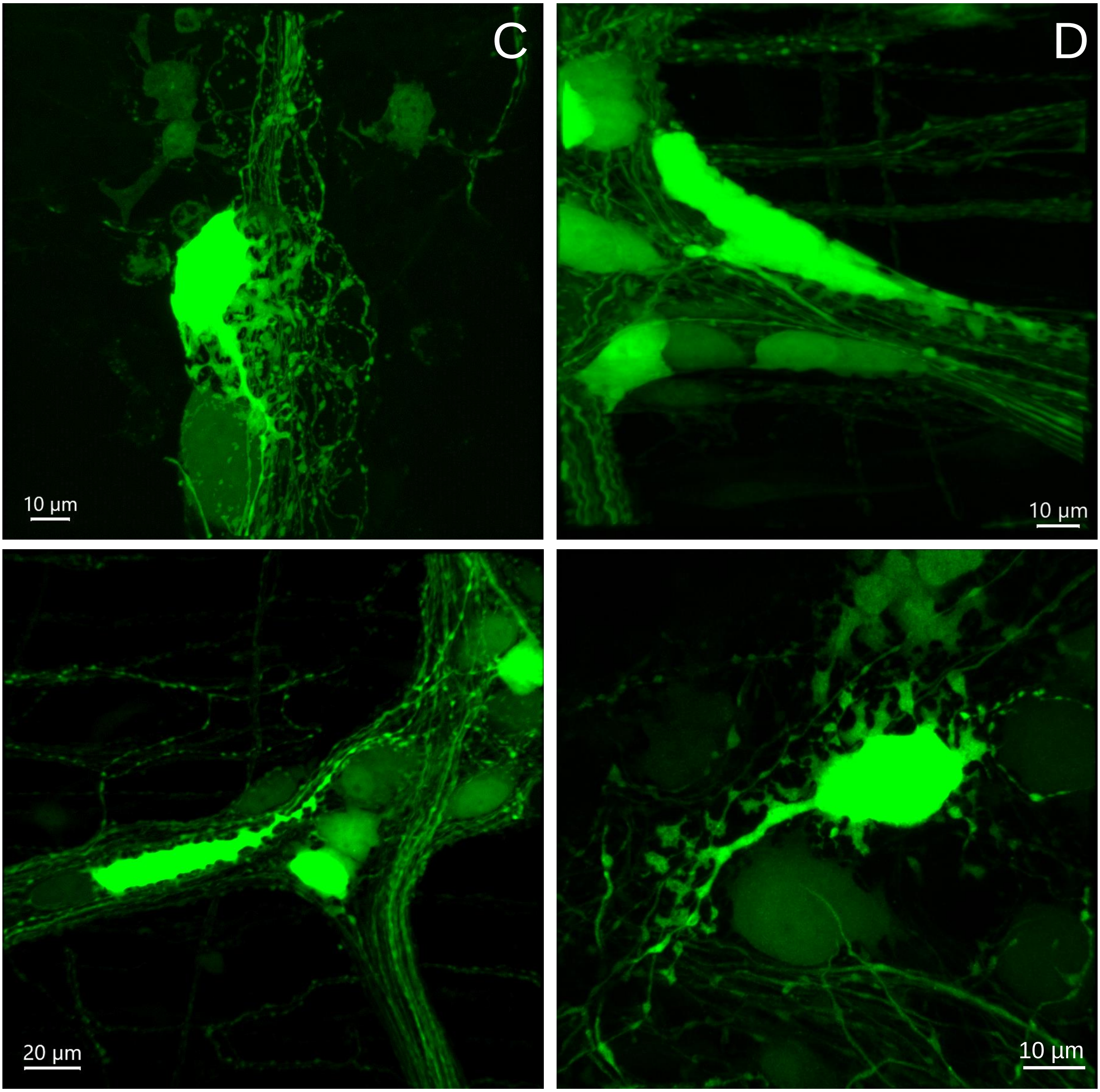

Supplementary Figure 32. Morphology of AAV9-tranduced neurons in the mouse proximal colon. **A.** a group of 5 neurons in a submucosal ganglion were closely adjacent to one the other via the somas. **B.** two neurons in a myenteric ganglion were closely adjacent to each other by the somas. **C** and **D**. examples of neurons showed unusual morphology in the submucosal (C) and myenteric plexus (D).
